# Monitoring the Environmental Degradation of Biodegradable Mulch Films Using Py-GC-MS and a Multitechnique Analytical Approach

**DOI:** 10.1021/acs.est.6c11149

**Published:** 2026-10-21

**Authors:** Francesca De Falco, Tommaso Nacci, Fannie Burgevin, Winnie Courtene-Jones, Allicia Fullarton, Rachel L. Coppock, Ilaria Degano, Francesca Modugno, Richard C. Thompson

**Affiliations:** † School of Biological and Marine Sciences, 6633 University of Plymouth, Drake Circus, Plymouth PL4 8AA, U.K.; ‡ Department of Earth Sciences, University of Bayreuth, Universitätsstraße 30, Bayreuth 95447, Germany; § Department of Chemistry and Industrial Chemistry, 154932 University of Pisa, via G. Moruzzi 13, Pisa 56124, Italy; ∥ Institute for Sustainability and Department of Chemistry, 1555 University of Bath, Bath BA2 7AY, U.K.; ⊥ School of Ocean Science, Bangor University, Menai Bridge LL59 5AB, U.K.; # Marine Ecology & Society, Plymouth Marine Laboratory, Prospect Place, Plymouth PL1 3DH, U.K.

**Keywords:** Field tests, SEM, SEC, FTIR, NMR, chemical leaching, pyrolysis markers, alternative plastics

## Abstract

Biodegradable mulch films have been developed for use in agriculture to reduce costs incurred during the retrieval of conventional mulch films and to prevent plastic pollution of soil caused by their incomplete removal. However, there are knowledge gaps on their degradation rate and potential environmental impact of any residuals. This study investigated the degradation of three commercially available mulch films, one made of polyethylene and two labeled as “biodegradable”. These products were exposed to four conditions in the open environment. After 24 months in soil, air, and a combination of air pre-exposure for 3 months followed by soil burial, all samples retained their overall film structure and were still recoverable, although different levels of physical and chemical changes indicated ongoing degradation. In seawater, no residual fragments of the biodegradable mulch films remained after 3 months of exposure. Pyrolysis-gas chromatography-mass spectrometry detected leaching of a plasticizer from one of the biodegradable films in all environments and other degradation features. The outcome of this study highlights the potential for environmental accumulation of mulch films labeled as “biodegradable” and the need for the development of international environmental testing standards and associated labeling to guide users on appropriate use and disposal of mulch films.

## Graphical abstract

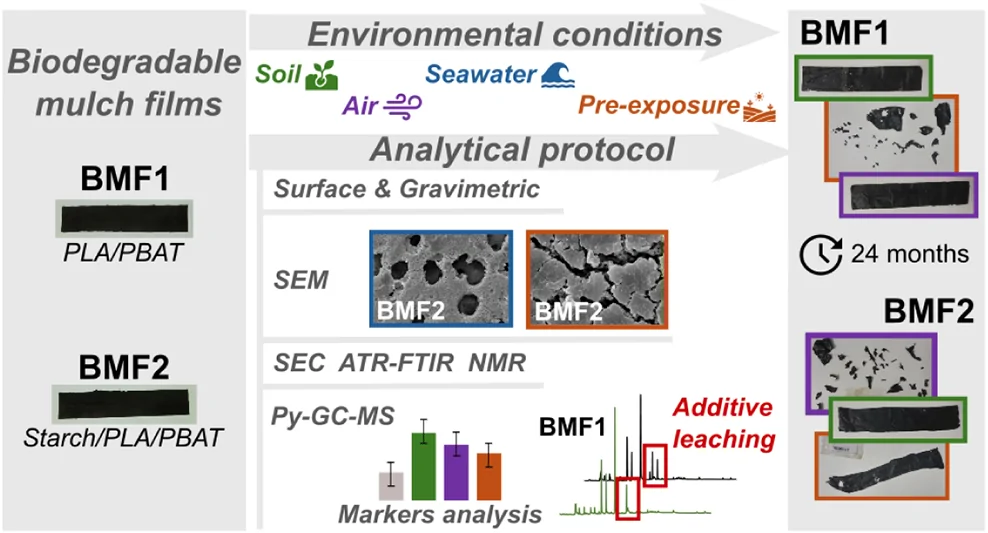

## 1 Introduction

The use of plastic materials in agricultural practices has increased rapidly reaching >12.5 million tonnes yr^–1^,^1^ a quantity representing almost 3% of the 430.9 million tonnes of plastic produced globally in 2024.^2^ Applications include tree shelters, pesticide containers, and mulch films. The latter can substantially increase agricultural productivity through weed inhibition, reduction in access for pests and hence a reduction in the use of pesticides, reduction in nutrient loss, improved soil temperature control, and early germination.^3,4^ Plastic mulch films are commonly made from poly­(ethylene) (PE) whose retrieval, recycling, or landfilling is challenging, due to fragmentation and contamination with soil, leading to plastic remaining in the environment.^3,5^ In addition, the degradation and fragmentation of mulch films generate microplastics (MPs), which accumulate in soil and affect soil physical properties, soil biota, and plant growth.^6–9^ Biodegradable polymers are increasingly promoted for use in mulch films as sustainable alternatives to conventional PE mulch films and as a potential means of reducing plastic accumulation in soil.^4,5^ Indeed, it has been suggested that these alternative plastic materials may bring benefits in applications where plastic products are difficult/ not possible to retrieve from the environment at their end of life, providing that the biodegradable plastic product degrades within reasonable time scales and without generating harmful MPs or chemical leachates.^10^ Among the biodegradable polymers most used for mulching applications, poly­(butylene adipate-*co*-butylene terephthalate) (PBAT) is commonly used and often blended with poly­(lactic acid) (PLA) and/or thermoplastic starch.^11,12^ In the global production of bioplastics (i.e., biobased and/or biodegradable plastics), PLA, PBAT, and starch-containing polymer compounds account for 47.4% of the 2.47 million tonnes produced in 2024.^13^ Potential environmental impacts have been studied^14,15^ and, for instance, PBAT mulch films incorporated into soil caused a decrease in microbial biomass and soil pH, similar to PE films,^16^ and plastic additives are reported in soils where biodegradable mulch films were used.^17^ While there are standards relating to the biodegradation of mulch films, such as EN 17033 ^18^, ASTM D6400 ^19^ and the ecolabel “OK Biodegradable SOIL” (TÜV Austria),^20^ these are based on controlled-condition laboratory tests using respirometry to assess biodegradation^18^ rather than tests of performance in the open environment. The transferability of laboratory tests to biodegradation in the open environment is limited, partly because real-world receiving environments vary considerably in terms of environmental and soil conditions (e.g., temperature, humidity, microbial communities, etc.).^5,21^ Biodegradation is a system property, resulting from the interplay among the specific material properties and the abiotic and biotic conditions of the receiving environment.^10,22–24^ Some studies investigated the biodegradation of commercially available mulch films under field conditions by burying them in soil, reporting incomplete degradation over the investigated periods (ranging from 7 to 36 months) and the formation of macro- and microplastics.^25–29^ However, because of differences in film composition, exposure conditions, experimental design, and analytical approaches used to assess degradation, direct comparison among studies is not possible. In addition to soil exposure, biodegradable mulch films left in the environment may be transported to other ecosystems and subjected to different environmental conditions. Recent studies have shown that agricultural plastics can travel over 100 km from cultivation areas to offshore environments, with riverbeds acting as temporary sinks during dry periods and releasing plastic debris to marine ecosystems after rainfall events.^30^ Mulch films have also been identified as a significant source of microplastics in riverside and estuarine sediments.^31^.

In this scenario, there are still knowledge gaps regarding biodegradable mulch films degradation rate and extent under field-relevant environmental conditions, and about the relevance of the existing international standards.^5,10,32^ To this aim, the present study provides a comprehensive investigation of the environmental degradation of commercial mulch films, labeled as “biodegradable”, under different field conditions, combining multiple analytical techniques to capture both physical and chemical changes..

We monitored the degradation process of two types of mulch films labeled as “biodegradable” and one conventional PE film (as negative control) in four environmental exposure conditions for up to 24 months: buried in soil, exposed to air, submerged in seawater, and a combination of air pre-exposure followed by soil burial. The analytical protocol applied in this study to examine changes in the characteristics of these materials encompassed both superficial and bulk techniques. In particular, we also used Pyrolysis coupled to Gas Chromatography and Mass Spectrometry (Py-GC-MS) to investigate changes at the molecular level, enabling the detection of variations in polymer-specific markers as well as the release or alteration of organic additives.

## 2 Materials and Methods

### 2.1 Materials

Three types of commercially available agricultural mulch films were considered within these experiments: 2 labeled as being “biodegradable” (BMF1, BMF2) and 1 conventional mulch film (CMF) made of PE as negative control (Table 1). The composition of the two mulch films BMF1 and BMF2 has already been characterized in previous work.^33^

**Table 1:** Set of Mulch Film Samples Analysed

Product code	Film thickness [mm]	Color	Product composition^a^	Standard compliance^a^
**CMF**	0.020	Black	PE	-
**BMF1**	0.010	Black	Blend of PLA and PBAT	EN 13432
**BMF2**	0.015	Black	Mater-Bi	EN 13432; EN 17033; ASTM 6400; OK Biodegradable Soil

a As declared by the manufacturer.

### 2.2 Field Deployment

Samples were prepared by cutting the mulch films into strips measuring 3 × 15 cm. Triplicate samples of each type of mulch film were prepared for each of the 5 sampling time points considered over the course of the experiment (1, 3, 6, 12, and 24 months after initial deployment). Samples were tested under four different environmental conditions (further details below).

Each sample was contained within a PE mesh pouch (0.8 mm mesh aperture) measuring 5 × 20 cm. The edges of each pouch were closed using a heat sealer, and samples were labeled with a laminated tag. Samples were deployed between the 1st and 3rd of September 2021.

The weight (4 d.p.) and surface area (image analysis using ImageJ) of all samples were recorded prior to deployment.

#### 2.2.1 Soil Exposure Conditions

An agricultural field margin (50.53535° N; 4.27868° W) (Figures 1a and S1a in the Supporting Information, SI), which had been uncultivated for >5 years, was used as the soil exposure test site. To ensure contact between the soil and the mulch film sample contained in the mesh pouch, soil (100 g w.w.; taken from 10 cm depth from the site) was added to each pouch ensuring both sides of the samples (coded as Soil) were in direct contact with the soil, prior to fully sealing the pouch. Then, the pouches were buried at a depth of approximately 10 cm (Figure S1b in the SI). Two HOBO pendent temperature loggers were buried at the same depth as the samples (Figure S2 in the SI). Information on soil characteristics is provided in the SI (Table S1).

**Figure 1:**
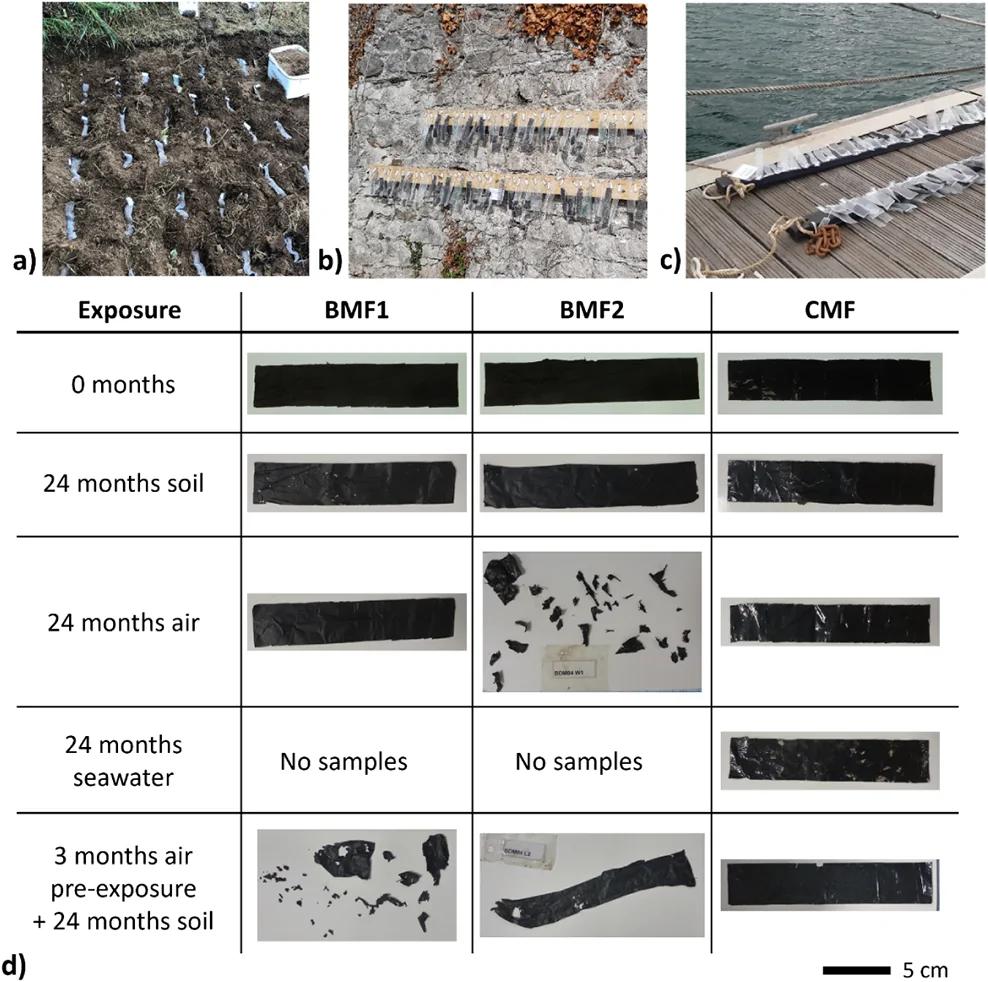
Pictures of the samples in mesh pouches: a) prior to soil burial at a depth of 10 cm; b) fixed to the south-facing wall undergoing air exposure; c) fixed to the beam deployed in the seawater environment at a depth of 1.5 m; and d) representative images of samples following 24 month exposure to the 4 different environmental conditions, compared to the reference samples (not exposed to environmental conditions).

#### 2.2.2 Air Exposure Conditions

Samples (coded as Air) contained within mesh pouches were affixed to wooden beams located on a south-facing wall at the University of Plymouth’s Skardon Garden (50.37748° N; 4.13686 °W) (Figures 1b and S3 in the SI) and exposed to environmental conditions including UV, rain, wind, etc. External temperatures and weather conditions were retrieved from the local weather station (Figure S4 in the SI).

#### 2.2.3 Seawater Exposure Conditions

Samples (coded as Marine) placed in the marine environment were submerged on a beam at Queen Anne’s Battery Marina (50.36587 °N; 4.13164 °W) at a depth of approximately 1.5 m (Figures 1c and S5 in the SI). Weights were connected to each side of the beam to maintain depth. Two HOBO pendent temperature loggers were attached to the beams (Figure S6 in the SI). The salinity of the seawater measured in December 2021 was 29 ppt. Based on initial observations (Section 3.1), two additional seawater exposure experiments were performed to better understand the behavior of the mulch films when submerged in seawater (Table S2 in the SI). The first experiment (coded as Seawater1) replicated the original experiment, with a deployment in the same month 1 year later (late summer) with triplicate films sampled at shorter intervals to trace early stages of degradation (after 1, 3, 4, and 5 weeks). The second set of experiments (coded as Seawater2) was performed deploying the samples during a different season time (late winter), with sampling after 1, 2, 3, 4, and 5 weeks (see the SI for further details).

#### 2.2.4 Air Pre-exposure + Soil Exposure

Samples (coded as Pre-exposure) in mesh pouches were exposed to air conditions on the surface of the soil for 3 months, before they were buried at a depth of 10 cm in the agricultural field margin. During the exposure on the soil surface, the area was weeded ∼monthly, to ensure that vegetation did not restrict light reaching the samples. Samples were prepared as described above for the soil exposure (soil was added to each pouch to ensure contact with both sides of the mulch film sample) and were deployed on the soil surface on 3rd September 2021 and buried in the soil on 6th December 2021.

### 2.3 Sample Collection

Samples were collected on each date and placed into individual zip-lock bags for transport to the laboratory. Here, they were removed from the pouches, washed with deionized water, and dried at 40 °C. Then, the samples were visually inspected for holes or disintegration, weighed (4 d.p.), and photographed to derive the surface area, calculated using the software ImageJ (release 1.43u). Further details on sample collection are reported in the SI (paragraph “2. Sample collection”).

### 2.4 Characterization Techniques

Soil, air, marine, and pre-exposure mulch film samples collected after 1, 3, 6, and 12 months of exposure underwent characterization analyses (below) to study the degradation process, while the samples collected after 24 months were analyzed by visual inspection and gravimetric analysis only.

A range of analytical techniques were used to provide specific information on the samples: morphological analysis of the surface of the samples was performed by Scanning Electron Microscopy (SEM); Size Exclusion Chromatography (SEC) was performed to monitor changes in the molar mass distribution of the polymers; Attenuated Total Reflectance Fourier Transform Infrared Spectroscopy (ATR-FTIR) and Nuclear Magnetic Resonance Spectroscopy (NMR) were performed to detect changes in the functional groups and in the polymer phases, respectively. Moreover, Py-GC-MS analyses were performed to investigate the degradation mechanisms at molecular level. Further details on the characterization techniques are provided in the SI. The interpretation of the Py-GC-MS pyrolysis profiles and the identification of the pyrolysis products were based on previous work.^33^ First, the Py-GC-MS chromatograms were qualitatively compared to highlight changes in the profile, such as the disappearance of specific peaks after exposure or the appearance of new peaks attributable to degradation products. Second, we evaluated the changes in the abundances—estimated through integration and normalization of peak areas—of the characteristic pyrolysis markers of the main polymer components of the mulch films, PLA and PBAT.^33^ In Py-GC-MS, the chromatographic peak area is proportional to the concentration of the corresponding molecule in the pyrolyzate, and the analysis of polymer-specific pyrolysis products can be associated with distinct polymeric components in the sample.^34^ Thus, their abundance can be used under controlled conditions (consistent matrix composition and thus pyrolysis yield) to semiquantitatively estimate changes in the relative abundance of the corresponding polymeric fractions in the analyzed sample. However, in the absence of calibration curves, these variations can only be interpreted in a comparative manner, reflecting relative rather than absolute changes in polymer content.^34,35^ Moreover, changes in the relative abundance of pyrolysis products from the same polymer can in turn provide insights on its chemical alteration or degradation.^35–37^

The chosen PLA pyrolysis markers are the lactide isomers 3,6-dimethyl-1,4-dioxane-2,5-dione in the *meso*- and *DL*-forms, generated through intramolecular transesterification during the pyrolysis process. These are the most specific pyrolysis products of PLA. PBAT markers include products formed by the pyrolysis of the adipic and terephthalic moieties (i.e., 3-butenyl ester of benzoic acid, di­(3-butenyl) esters of adipic and terephthalic acids) and the lactone 1,6-dioxacyclododecane-7,12-dione produced through the cyclization of adipic acid and 1,4-butanediol. In this case, the two most abundant pyrolysis products were chosen along with two minor products, which are specific for this copolymer and that we detected in commercial mulch films.^33^ In the case of BMF2, the starch markers pyruvic aldehyde and levoglucosan were also considered, as they are known molecular markers of cellulosic materials.^38^ The areas of the peaks associated with the above-described main PLA, PBAT, and starch pyrolysis products were integrated in the Total Ion Current (TIC) Py-GC-MS chromatograms, normalized by the sample weight and by the area of the peak associated with the internal standard (anthracene-d10), and compared to evaluate changes related to alteration during exposure. The normalization by the internal standard is necessary to take into account the variabilities in the instrumental response among different analysis batches.

### 2.5 Statistics

Statistical analysis of the weight/surface loss data and of the normalized peak areas of the pyrolysis products, all acquired in triplicates, was carried out by using IBM SPSS Statistics software to evaluate significant differences. Data normality and homogeneity of variance were assessed using the Shapiro–Wilk and Levene’s tests, respectively. Differences among groups were evaluated using independent-samples *t*-tests or one-way analysis of variance (ANOVA) followed by a Student–Newman–Keuls (SNK) post hoc test; when homogeneity of variance was not met, Welch’s ANOVA with Games-Howell post hoc testing was applied. For weight and surface loss data, ANOVA was performed only on post-exposure time points (0 months was not included), as the initial condition corresponds to 0% loss by definition and therefore has no experimental variance. The initial condition was used as a theoretical baseline for one-sample *t*-tests against 0%, with Holm–Bonferroni correction applied when multiple time points were tested within the same exposure condition. Statistical significance was set at *p* < 0.05 or p_adj_ < 0.05. More details are reported in the SI.

## 3 Results and Discussion

### 3.1 Visual Inspection, Weight Loss, and Surface Area Analyses

After 24 months of environmental exposure, the majority of the samples remained structurally intact, with some cracks and holes visible. However, advanced fragmentation was observed for BMF2 exposed to air and BMF1 pre-exposed (see Figure 1). Exposure to seawater resulted in rapid loss of material: no material or fragments could be detected in the pouches containing BMF1 and BMF2 samples after 3 months, meaning that data are only available after 1 month of exposure. The underlying cause of this loss of material remains uncertain: it is unclear whether the samples underwent complete biodegradation or whether biological interactions fragmented the plastics. Further experiments (Seawater1 and Seawater2) were performed to specifically investigate the behavior of BMF1 and BMF2 under marine conditions and are discussed below.

The recorded data on weight and surface loss are plotted in Figures S7–S9 in the SI and underwent statistical analysis. For both BMF1 and BMF2, one-sample *t*-tests followed by Holm–Bonferroni correction showed that weight loss values did not differ significantly from the theoretical baseline of 0% (unexposed condition) for most time points, including after 24 months, in soil, air, and pre-exposure conditions (p_adj_> 0.05, see Tables S3–S4 in the SI). ANOVA revealed significant differences among time points only for BMF1 weight loss after soil exposure (ANOVA: F­(4,10) = 5.34, p = 0.01, Table S3a in the SI), BMF1 surface loss after air exposure (ANOVA: F­(4,10) = 4.54, p = 0.02, Table S3b in the SI), and BMF1 surface loss under pre-exposure conditions (Welch ANOVA: F­(5,5.32) = 9.62, p = 0.01, Table S3d in the SI). However, the corresponding post hoc tests did not reveal a consistent time-dependent trend. For seawater exposure, one-sample *t*-tests showed a significant difference from the theoretical baseline only for BMF1 surface loss after 1 month (t(2) = 1.69, p = 0.01, Table S3c in the SI). For CMF, one-sample *t*-tests followed by Holm–Bonferroni correction showed that weight and surface loss values did not differ significantly from the theoretical baseline for most time points under soil, seawater, and pre-exposure conditions (p_adj_> 0.05, see Table S5 in the SI). A significant difference was detected after 24 months of air exposure but only for surface loss (p_adj_ = 0.02, Table S5b in the SI), not for weight loss (p_adj_ = 0.08, Table S5b in the SI). Additional ANOVA on CMF data detected significant differences among the time points of the pre-exposure samples for both weight (Welch ANOVA: F­(5,5.19) = 9.71, p = 0.01) and surface (ANOVA: F­(5,12) = 4.64, p = 0.01) loss, but post hoc tests did not detect relevant trends.

Although BMF2 is certified according to EN 17033, the persistence of the samples after 24 months in soil highlights a discrepancy between standardized laboratory biodegradation criteria and degradation behavior under the tested field conditions. As confirmed by the statistical analysis, for both BMF1 and BMF2, no environmentally meaningful loss of weight and surface was recorded after 24 months of exposure and with samples of both materials remaining visibly intact at the end of the exposure period (excluding seawater conditions). Napper et al.^39^ used a similar exposure approach, reporting that bags labeled as compostable were no longer present after 3 months in seawater, consistent with our results, while soil-buried samples showed no measurable surface area loss after 27 months and air-exposed samples fragmented after 9 months. However, as no chemical characterization was performed in their study, their results cannot be directly compared with ours. Similar outcomes have been reported in the literature for PBAT-based agricultural mulch films exposed for ∼2 years. Sintim et al.^29^ studied three different PBAT-based mulch films, exposing them in an agricultural field for 5 months before burying them in soil in two different locations in the US (Knoxville and Mount Vernon) for 3 years. After 2 years of exposure, their mulch film samples were still present and after 36 months of exposure reached degradation (calculated by the authors considering the surface area reduction) in the ranges 61–83% in Knoxville and 26–63% in Mount Vernon (US). In field experiments in Beijing (China), Qi et al.^27^ observed that after 26 months of soil burial of four PBAT-based mulch films, visible plastic fragments of size <5 mm remained in soil, raising a question about possible microplastic accumulation over time and mulch films usage. BMF2 is certified according to EN 17033, whose criteria are defined under controlled laboratory conditions using ISO 17556. This latter includes using a standardized soil (made of natural soil, mature compost, kaolinite clay, and industrial quartz sand) in the temperature range 20–28 °C and with a C:N ratio of at least 40:1. In our work and the works previously mentioned,^27,29,39^ these standard conditions were not met because the experimental approach was to test materials in real field conditions, representative of exposure conditions where such mulch films are actually used. To assess the reproducibility of the unexpectedly rapid degradation observed under seawater exposure and to further investigate its causes, two additional seawater experiments were performed. The first one was performed in the same seasonal period (September–October, average temperature 16.68 °C ± 0.13 S.D.) of the original deployment and confirmed our findings. The results are reported in the SI (Paragraph “6. Additional seawater exposure experiments” and Figures S10-11). After 4 weeks, BMF2 lost 18 ± 7% in weight (21 ± 15% in the original experiment), whereas BMF1 showed a faster degradation/fragmentation rate losing 89 ± 3% of its weight (25 ± 25% in the original deployment). The second seawater deployment was performed in a different seasonal period (February–March, average temperature 9.79 °C ± 0.09 S.D.) with weight losses <10% for both films. Possible reasons for the difference between the results of the two deployments could be seasonal variations in environmental conditions such as temperature (in the second redeployment, the average temperature was 6.89 °C ± 0.16 S.D. colder) and/or the presence of different organisms and microbial activities.^40^

### 3.2 SEM Analysis

The analyses showed that BMF1 and BMF2 had similar changes in surface morphology, depending more on the exposure conditions than on the material type. In both air and soil exposures, circular holes of nanodimensions were observed after 1 month. The morphology of the holes was similar between the two materials, but they were more homogeneously present on the surface of BMF2, which is made of Mater-Bi (starch/PLA/PBAT) (Figures 2 and S12). However, the presence of circular holes on the surface of Mater-Bi could be due to the degradation of the starch grains dispersed in the polymeric matrix.^25,26,41^ Nevertheless, since no starch was detected in BMF1, the presence of circular holes on its surface must be due to other mechanisms such as mechanical abrasion or microbiological activity. The presence of similar holes has also been reported in PBAT samples after 39 days of exposure in digested sludge (attributed to polymer chain breakage),^42^ in PLA samples buried in soil for 5 months,^43^ and in PLA/PBAT blends also buried in soil.^44^ In the present study, as the exposure time increased, the holes became more abundant, and after 12 months of exposure in soil, tears and rips became apparent on the surface of both BMF1 and BMF2 (Figure 2). In addition, longitudinal cracks were observed on the BMF2 samples exposed to air for 12 months. For the samples exposed for 1 month in seawater (Figures 2 and S13), both BMF1 and BMF2 appeared to be severely damaged with large circular holes and cracks present on their surfaces, especially for BMF2. BMF1 samples that underwent pre-exposure conditions showed, similar to air and soil samples, visible holes after 3 months of pre-exposure to air conditions (Figures 2 and S14). After 3 months of burial in soil, cracks started to appear on the surface of the film, which became more abundant over exposure time.^24,45,46^ Regarding BMF2 samples in pre-exposure conditions, again holes were observed after 3 months of pre-exposure to air and their morphology started to evolve in cracks after 1 month of soil burial, and the cracks increased in size across the soil exposure period (Figure 2). Compared with samples exposed only to soil or air, samples subjected to the combined air pre-exposure and soil burial exhibited more extensive surface deterioration. The formation of cracks (pre-exposure) may be due to the pre-exposure conditions (e.g., UV interaction) that weakened the material itself. In fact, surface cracks with a similar morphology were also observed by Convertino et al.^25^ in samples artificially UV-aged and then buried in the soil. Unexposed CMF samples presented some grooves on their surfaces, but no morphological changes were observed up to 12 months across all the exposure conditions, only the formation of some biofilm was observed on marine samples (Figure S15 in the SI).

**Figure 2:**
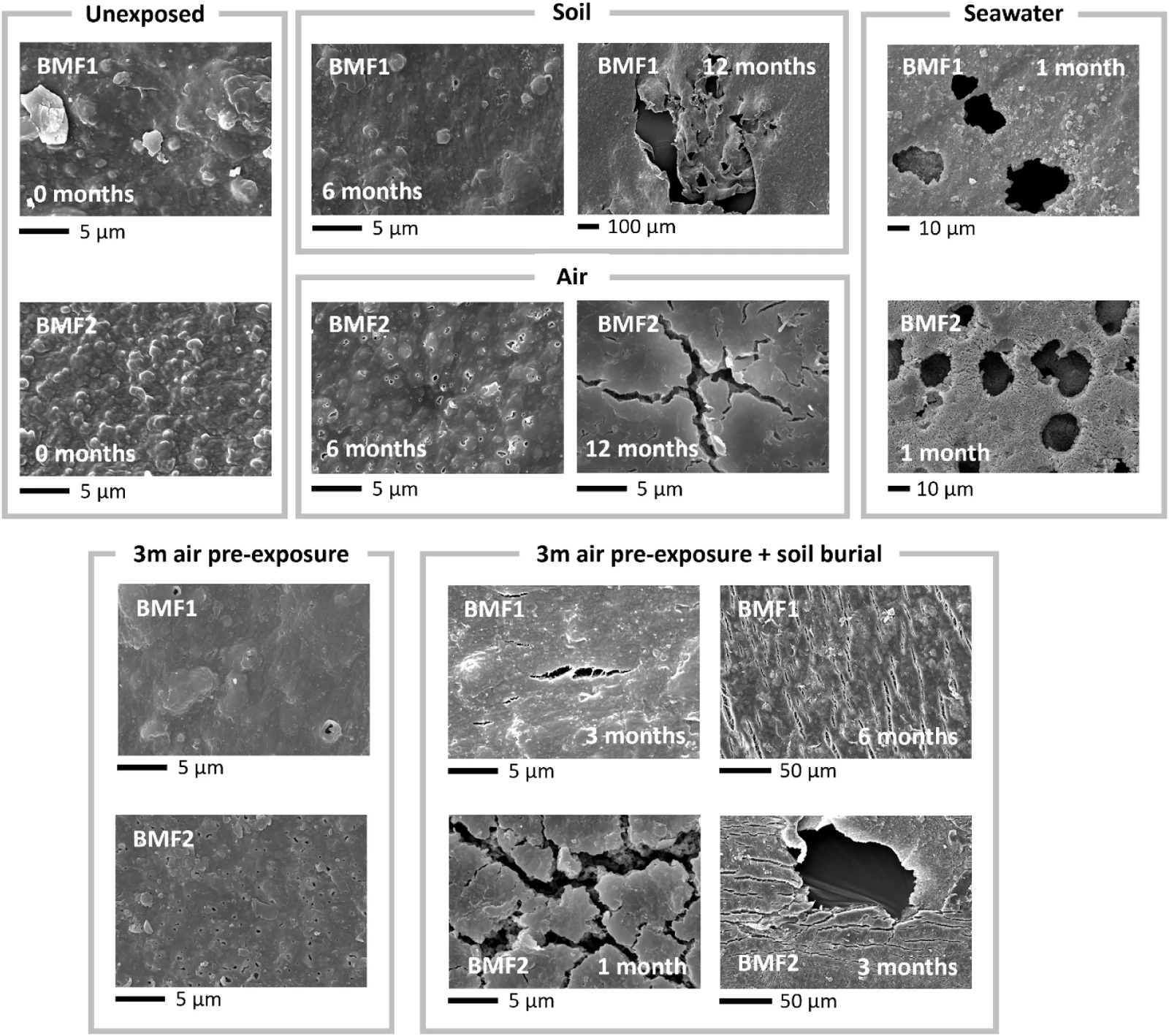
SEM micrographs of BMF1 and BMF2 samples unexposed (0 months), exposed to soil, air, and seawater for 1, 6, or 12 months, and pre-exposed to air for 3 months and then buried in soil (3 and 6 months for BMF1 and 1 and 3 months for BMF2).

### 3.3 SEC Analysis

Analysis of the molar mass enabled assessment of changes in chain length in the bulk sample. Changes in molar mass are presented in Figure S16. Results for BMF1 showed the most pronounced decrease for samples in the combined pre-exposure conditions, consisting of 3 months of air exposure followed by soil burial, with the average molar mass reaching 50 ± 4% of the initial value after 12 months of subsequent soil burial. However, because these samples underwent a longer total exposure time than samples exposed only to soil or air, this result cannot be attributed solely to an accelerating effect of pre-exposure. Rather, it indicates that the combined air–soil exposure conditions resulted in the lowest molar mass values among the conditions tested. A more limited change was observed for samples buried in soil without prior exposure in air reaching 78 ± 1% of the initial molar mass after 12 months, whereas samples exposed only to air reached 62 ± 3% after 12 months. The BMF1 samples exposed to seawater were analyzed after 1 month and showed a marked decrease in molar mass, reaching 55 ± 5% of the initial value, indicating rapid chain scission under seawater exposure conditions.

For BMF2, a decrease in molar mass was also observed in the combined air pre-exposure and soil burial scenario, reaching 48 ± 15% of the initial value after 3 months of air exposure followed by 12 months of soil burial. In contrast, samples buried directly in soil showed little or no decrease in molar mass over the same burial period. Under air exposure, BMF2 showed a slower and less pronounced decrease than BMF1, reaching 78 ± 12% of the initial molar mass after 12 months. Exposure to natural seawater for 1 month also caused a decrease in molar mass, although this was less pronounced than for BMF1, with BMF2 reaching 69 ± 11% of the initial value. Overall, BMF1 generally showed a larger decrease in molar mass than BMF2 under air and seawater exposure, while differences under soil burial were less pronounced. The higher variability observed for BMF2 may be related to the greater compositional complexity and heterogeneity of this material, which contains starch, PLA, and PBAT, as well as to spatially heterogeneous degradation within the film. The presence of starch and the multiphase structure of BMF2 may influence water uptake, microbial colonization, and the accessibility of the polyester fraction to hydrolysis and biodegradation, leading to a less uniform degradation pattern. Previous studies have reported decreases in molar mass during degradation of biodegradable mulch films and related materials. Anunciado et al.^47^ reported a consistent decrease in molar mass during the exposure of PBAT-based and Mater-Bi mulch films under standard aerobic laboratory conditions, while Liu et al.^48^ observed similar trends for PBAT-based films in a simulated marine environment. In contrast, Convertino et al.^25^ found no significant polymer chain cleavage in the residual polyester fraction of pristine or artificially aged starch/PLA/PBAT mulch films buried in soil. In our study, SEC results indicate that chain scission occurred under several exposure conditions, with particularly pronounced decreases after seawater exposure and in the combined air–soil exposure scenario. These results are consistent with the SEM observations, which showed morphological changes associated with degradation.

### 3.4 ATR-FTIR and NMR Analyses

The spectra of the unexposed BMF1 and BMF2 samples were similar (Figures S17–S20) and featured the characteristic absorption bands of PBAT as reported in Table S6.^49,50^ Specifically, the spectra exhibited signals corresponding to ester groups, including C=O (1760 cm^–1^) and C–O (1269, 1166, 1101, 871 cm^–1^) stretching modes that are typical of PBAT-based materials. The ATR-FTIR spectra (Figures S17–S20, Table S6) of all aged samples that were labeled as “biodegradable” did not show any changes in the functional groups that could indicate hydrolysis of the ester groups.^51,52^ Other works that have tested commercial PBAT-based mulch films in soil burial either found no distinct changes in the FTIR spectra up to 7 months^27^ or reported a decrease of peak intensities of C=O stretching and C–H_2_ bending suggesting that hydrolysis occurred over 36 months of soil burial.^29^ At the same time, another study that examined the deterioration of PLA mulch films in field conditions (buried in soil, exposed on the soil surface, and then buried) reported a decrease in absorbance for the band corresponding to the C=O stretching.^28^ In our results, the only sign of oxidation observed was the presence of a shoulder at 1760 cm^–1^ on the carbonyl absorption band. The oxidation appeared more pronounced in BMF1 than BMF2. No significant changes were observed in the spectra of the CMF samples (Figure S21), which presented the characteristic absorption bands of polyethylene, as reported in Table S6.^53^

NMR analyses were performed on a selection of samples to examine the relative changes after exposure of the two main phases of the polymeric blends composing BMF1 and BMF2, i.e., PLA and PBAT (paragraph “10. NMR analysis” and Table S7 in the SI). For BMF1, the PLA content in unexposed samples was calculated to be 10%, but this increased to 26–28% in samples collected after 12 months of exposure to soil, air, and pre-exposed samples. This could be caused by preferential degradation of the PBAT component of BMF1 samples. Sintim et al.^29^ found similar results with an increase in the percentage of PLA content in samples of a PLA/polyhydroxy alkanoate (PHA) mulch film exposed in soil and attributed this to a preferential degradation of PHA.

Analyses on BMF2 showed a PLA content of 10% in unexposed samples, which declined slightly to 8–9% after 12 months for samples exposed to air and those pre-exposed and to 12% for samples exposed to soil for 12 months. The less pronounced increase in the relative content of PLA in the BMF2 exposed samples compared to BMF1 may be associated with differences in the composition of the two materials, for example, the presence of starch in BMF2 and not in BMF1, which could influence the overall degradation behavior of the polymer matrix.

### 3.5 Py-GC-MS Analysis

The effects of exposure to different field conditions were also evaluated by comparing the Py-GC-MS chromatograms between exposed and unexposed mulch films. In terms of qualitative composition, the disappearance of peaks originally present in the unexposed samples indicated leaching or chemical alteration of the corresponding components. Conversely, if new Py-GC-MS peaks are present after environmental exposure, we can reasonably consider these compounds as pyrolysis products associated with species or altered portions of the polymers generated during the degradation of the films. The single-shot Py-GC-MS chromatograms at 600 °C of all the samples analyzed are reported in Figures S22–S30 in the SI, while the main pyrolysis products detected in the unexposed samples are listed in Tables S8, S9, and S10 in the SI. Table 2 reports a list of the compounds identified in the samples exposed to the different environments, including at which exposure time they start to appear in the Py-GC-MS chromatograms.

**Table 2:** List of Compounds Detected in the Py-GC-MS Chromatograms at 600 °C of BMF1 and BMF2 Exposed Samples at the Time Points They Start to Appear^a^

Sample	#	Compound	Soil	Air	Marine	Pre-exposure	Retention time (min)	Main ions (*m*/*z*)
**BMF1**	1	Acetone	6 months	6 months	-	3 months pre	4.3	**43**, 58
	2	2-Butanone	6 months	6 months	-	3 months pre	4.9	**43**, 57, 72
	3	3-Buten-1-ol	6 months	6 months	-	3 months pre	6.0	39, **42**, 57, 72
	4	Cyclopropanecarboxaldehyde	6 months	6 months	-	3 months pre	6.4	**39**, 41, 42, 69, 70
	5	2-Cyclopenten-1-one	6 months	6 months	-	3 months pre	9.9	39, 53, 54, **82**
	6	Pentanoic acid	-	12 months	-	3 months pre	10.5	**60**, 73
	7	Styrene	1 month	1 month	1 month	3 months pre	10.6	51, 78, **104**
	8	Benzaldehyde	6 months	6 months	-	3 months pre	11.6	51, 77, 105, **106**
	9	Benzoic acid	1 month	1 month	1 month	3 months pre	13.6	51, 77, **105**, 122
**BMF2**	10	Pyruvic aldehyde	6 months	6 months	-	3 months pre	4.2	**43**, 45, 56, 144
	11	3-Buten-1-ol	6 months	6 months	-	3 months pre	5.9	39, **42**, 57, 72
	12	Cyclopropanecarboxaldehyde	6 months	6 months	-	3 months pre	6.4	**39**, 41, 42, 69, 70
	13	Toluene	6 months	6 months	-	3 months pre	8.5	65, **91**, 92
	14	2-Vinylethyl acetate	6 months	6 months	-	3 months pre	9.1	39, **43**, 54, 73
	15	2-Cyclopenten-1-one	6 months	6 months	-	3 months pre	9.8	39, 53, 54, **82**
	16	Styrene	6 months	6 months	-	3 months pre	10.6	51, 78, **104**
	17	Benzaldehyde	6 months	6 months	-	3 months pre	11.6	51, 77, 105, **106**
	18	3,6-Dimethyl-1,4-dioxane-2,5-dione (*meso* form)	6 months	3 months	1 month	3 months pre	13.0	43, 45, **56**, 144
	19	3,6-Dimethyl-1,4-dioxane-2,5-dione (*DL* form)	3 months	1 month	1 month	3 months pre	13.3	43, 45, **56**, 144
	20	Levoglucosan	6 months	6 months	-	3 months pre	16.2	43, 57, **60**, 73, 98, 144

a The corresponding peaks (#) are shown in Figure 4. Samples pre-exposed for 3 months to air are indicated as “3 months pre”. The base peak in the mass spectrum of each compound is highlighted in bold.

#### 3.5.1 Qualitative Analysis of the Pyrolysis Products

For the samples of BMF1, two peaks associated with the compounds 1,6-dioxacyclohexadecane-7,16-dione and di­(3-butenyl) ester of sebacic acid were clearly detected in the pristine material, whereas these compounds were no longer observed in the pyrograms of the exposed samples at the first time point of exposure, i.e., 1 month for soil, air, and marine samples and 3 months for samples pre-exposed to air (Figure 3). This observation is important because the di­(3-butenyl) ester of sebacic acid is a pyrolysis product of the plasticizer dibutyl sebacate andtherefore, its absence in the Py-GC-MS chromatograms provides evidence of plasticizer leaching into the environment already during the first month of exposure.^54^ In our previous characterization study,^33^ the lactone 1,6-dioxacyclohexadecane-7,16-dione was tentatively assigned as a copyrolysis product of PLA and PBAT. However, it was concurrent with the disappearance of the di­(3-butenyl) ester of sebacic acid, suggesting that it is a pyrolysis product deriving from the plasticizer, rather than from the polymeric matrix.

**Figure 3:**
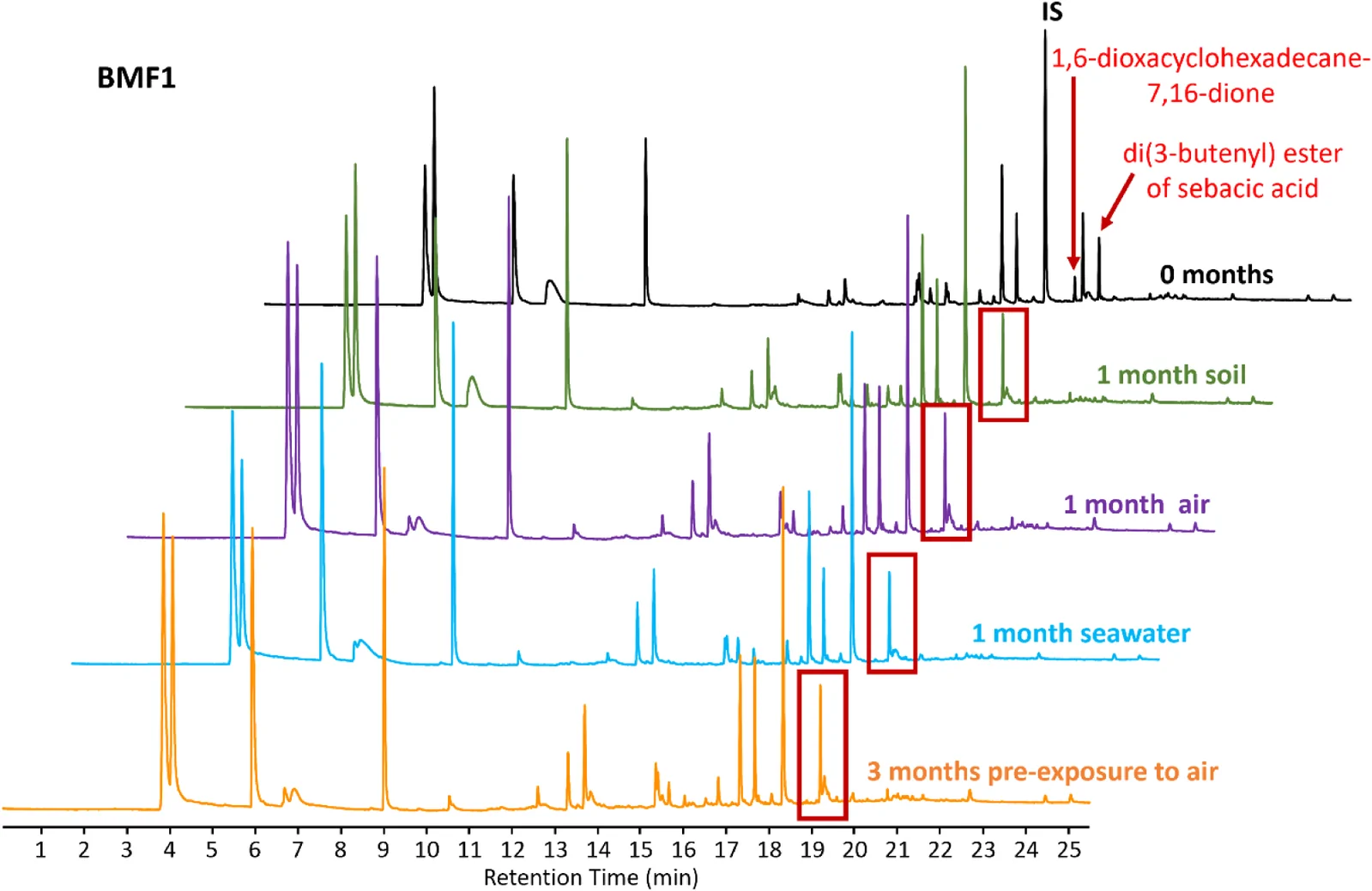
Py-GC-MS chromatograms at 600 °C of BMF1 samples unexposed (0 months), exposed to soil, air, or seawater environments for 1 month, and pre-exposed to air for 3 months. The two pyrolysis products of the plasticizer are highlighted by red arrows in the 0 m chromatogram; the red rectangles in the other chromatograms indicate the time range where the two products were expected to elute but were not detected. The internal standard peak is indicated with the label IS.

The Py-GC-MS chromatograms of the exposed samples also showed the appearance of new peaks that were not present in the Py-GC-MS chromatograms of the unexposed samples (all the Py-GC-MS chromatograms are reported in Figures S22–S25 and peak assignments in Table S8). These peaks correspond to unspecific low-molecular-weight species, and thus, it is not possible to unequivocally correlate them with a particular polymer in the mixture. Nonetheless, the detection of such pyrolysis products can be used to assess the degradation state of the mulch films, as the data clearly show that their presence depends on the exposure time and conditions. Figure 4 reports the Py-GC-MS chromatograms of BMF1 and BMF2 samples at the exposure time points when new pyrolysis products appear, and the list of peaks and identified compounds is reported in Table 2.

**Figure 4:**
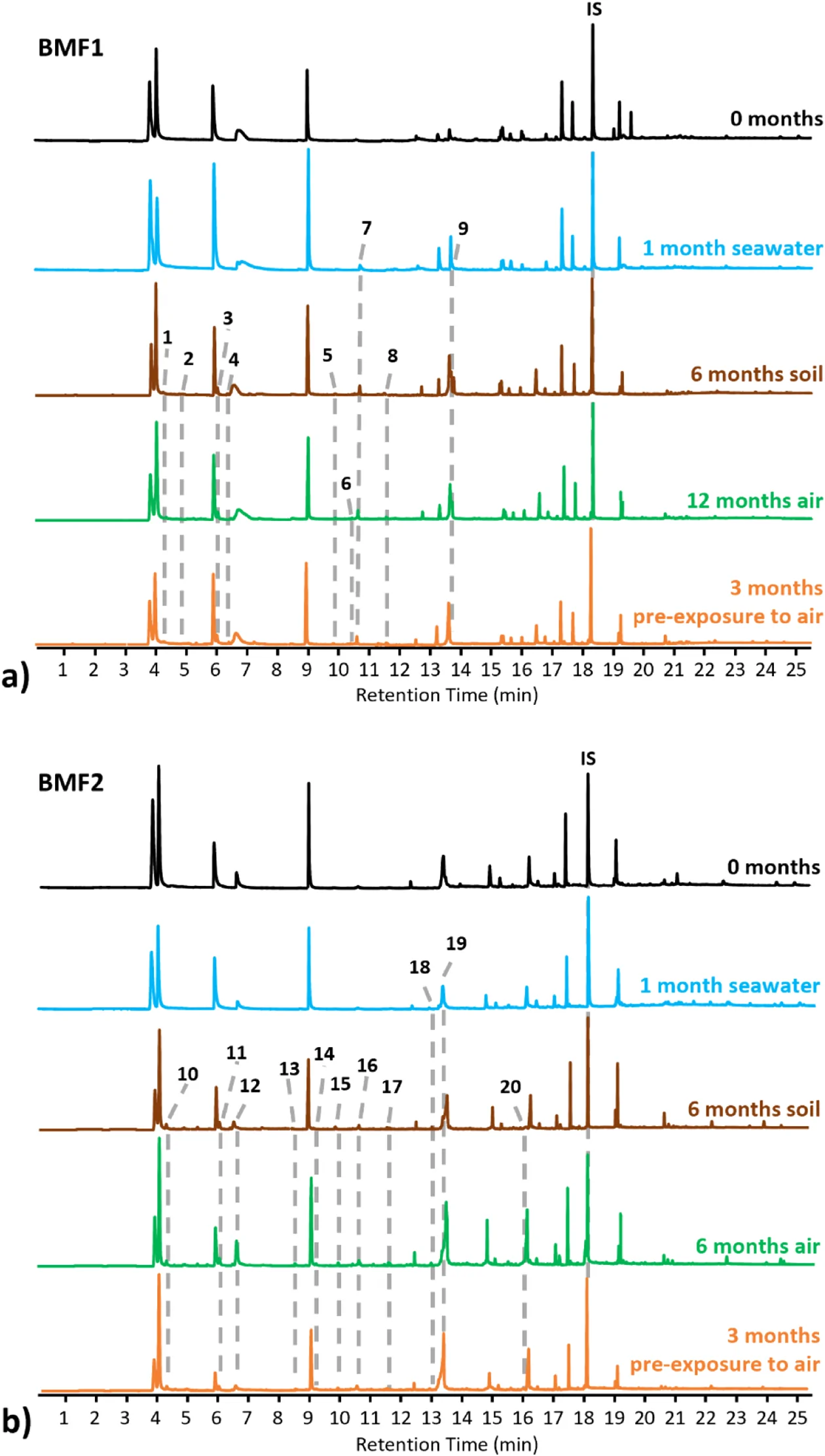
Py-GC-MS chromatograms at 600 °C of **a)** BMF1 and **b)** BMF2 samples unexposed (0 m), exposed to water for 1 month, to soil for 6 months, and to air for 6 months, and pre-exposed to air for 3 months. Degradation compounds (1–20) are reported in Table 2. The internal standard peak is indicated with the label IS.

As for BMF1, styrene, benzaldehyde, benzoic acid, and pentanoic acid likely derive from the degradation of PBAT. Styrene and benzoic acid are detected starting after 1 month of exposure in marine, soil, and air samples and in all pre-exposure ones. Benzaldehyde and other low-molecular-weight compounds such as acetone, 2-butanone, 3-buten-1-ol, cyclopropanecarboxaldehyde, and 2-cyclopenten-1-one were detected in the mulch films that exhibited more pronounced degradation, as the samples buried in soil or exposed to air for 6 and 12 months, as well as in all pre-exposure samples. Pentanoic acid was detected in samples exposed to air after 12 months and in all pre-exposure samples, suggesting that its generation, occurring through decarboxylation of the carboxylic acids (from the adipate moiety) as suggested by Coralli et al.,^35^ could be facilitated by UV irradiation.

As for BMF2 exposed samples, no leaching was observed (all Py-GC-MS chromatograms are reported in Figures S26–S29 and peak assignments in Table S9). However, the enhanced pyrolysis of the polymer caused by the weakening of the polymeric chains enabled the detection of PLA (i.e., *DL*- and *meso*-3,6-dimethyl-1,4-dioxane-2,5-dione) and starch (i.e., pyruvic aldehyde) pyrolysis markers in some of the exposed samples, as shown for BMF2 pre-exposed to air for 3 months in Figure 4b. These species were not detected in the Py-GC-MS chromatogram of the unexposed sample (Figure 4b), even if the presence of PLA was assessed by multishot Py-GC-MS analysis in a previous study,^33^ while levoglucosan reflects the presence of starch in the sample.^55^ In the exposed samples, starch markers were detected after 6 months in soil and air conditions and after 3 months of pre-exposure to air prior to soil burial in pre-exposure samples. PLA markers were observed in the Py-GC-MS chromatograms after 3 (lactide *DL* form) and 6 (lactide *meso* form) months of soil exposure and after 1 (lactide *DL* form) and 3 (lactide *meso* form) months of air exposure. The earlier detection of PLA markers in air samples is possibly due to a difference in the rate of degradation of the material under the two types of environmental conditions. Consistently, SEM and SEC analyses also showed greater surface damage and polymer chain scission for BMF2 samples exposed to air than in soil. PLA markers were also detected in all pre-exposure samples after 3 months of pre-exposure to air. Marine samples showed only the presence of PLA markers after 1 month but no signs of starch markers, possibly indicating complete degradation of the starch component in that time frame. As for the pyrolysis products derived from the degradation of BMF2 (i.e., 3-buten-1-ol, cyclopropanecarboxaldehyde, toluene, 2-vinylethyl acetate, 2-cyclopenten-1-one, styrene, and benzaldehyde), these were detected in samples buried in soil or exposed to air for 6 and 12 months, as well as in all pre-exposed samples. Some of these compounds (i.e., cyclopropanecarboxaldehyde, 2-cyclopenten-1-one, styrene, and benzaldehyde) are also common to the BMF1 exposed mulch films, and the changes in their abundances follow the same trend.

Regarding the conventional PE mulch film CMF, no changes in the chemical composition or in the relative abundance of PE pyrolysis products were detected. All of the pyrolysis profiles showed the typical features of PE, mainly peak triplets comprising the diene, the monoalkene, and the alkane for the relative C_n_ hydrocarbon (Py-GC-MS chromatograms and the corresponding table for peak assignments can be found in the SI, Figure S30 and Table S10, respectively).^56^ This suggests that the polymer did not undergo measurable degradation during 12 months of exposure.

#### 3.5.2 Semiquantitative Analysis of Pyrolysis Markers

In order to assess changes in the relative abundance of the pyrolysis products caused by environmental exposure, the peak areas of the pyrolysis markers of PLA, PBAT, and starch were integrated and normalized by sample weight and internal standard peak area; the resulting data are reported in the SI (BMF1: Figures S31 and S32; BMF2: Figure S33).

For BMF1, the relative intensities of PLA pyrolysis markers (i.e., lactide isomers *meso*- and *DL*-3,6-dimethyl-1,4-dioxane-2,5-dione) are higher in all the exposed samples compared to those unexposed (Figure S31a in the SI). This is consistent with the increase in the relative content of PLA for all the exposed samples observed by NMR, and this phenomenon could be a consequence of the degradation of PBAT, which results in a higher relative amount of the PLA fraction in the samples. This effect was particularly pronounced in the samples exposed to seawater for 1 month, which exhibited the highest relative abundance of PLA markers among all exposure conditions (Figure S31a). Comparison with unexposed samples confirmed a significant increase in both lactide isomers after exposure (Figure 5a), with normalized areas significantly greater than the unexposed material (*meso* form *t*-test: *t*(2.03) = −8.20, p = 0.01; *DL* form *t*-test: *t*(2.01) = −8.96, p = 0.01; Figure 5b). Samples exposed to seawater showed the most notable changes in terms of PBAT pyrolysis markers as well (Figure 5a). The pronounced degradation of PBAT in samples immersed in seawater is further evidenced by the statistically significant reduction in the normalized peaks associated with the PBAT markers di­(3-butenyl) esters of adipic acid (*t*-test: *t*(4)= 3.42, p = 0.03, Figure 5b) and terephthalic acid (*t*-test: *t*(4)= 9.09, p = 0.00, Figure 5b), and a possible explanation is the hydrolysis of the ester bonds leading to depolymerization. This can, in turn, be the reason that the samples could not be recovered after 1 month of seawater exposure. Such a decrease in the PBAT markers was not significant for the samples exposed in the other tested conditions (Figure S31b in the SI), suggesting that hydrolysis may be more efficient in seawater compared to soil and air due to the aqueous environment and/or microbial activity.

**Figure 5:**
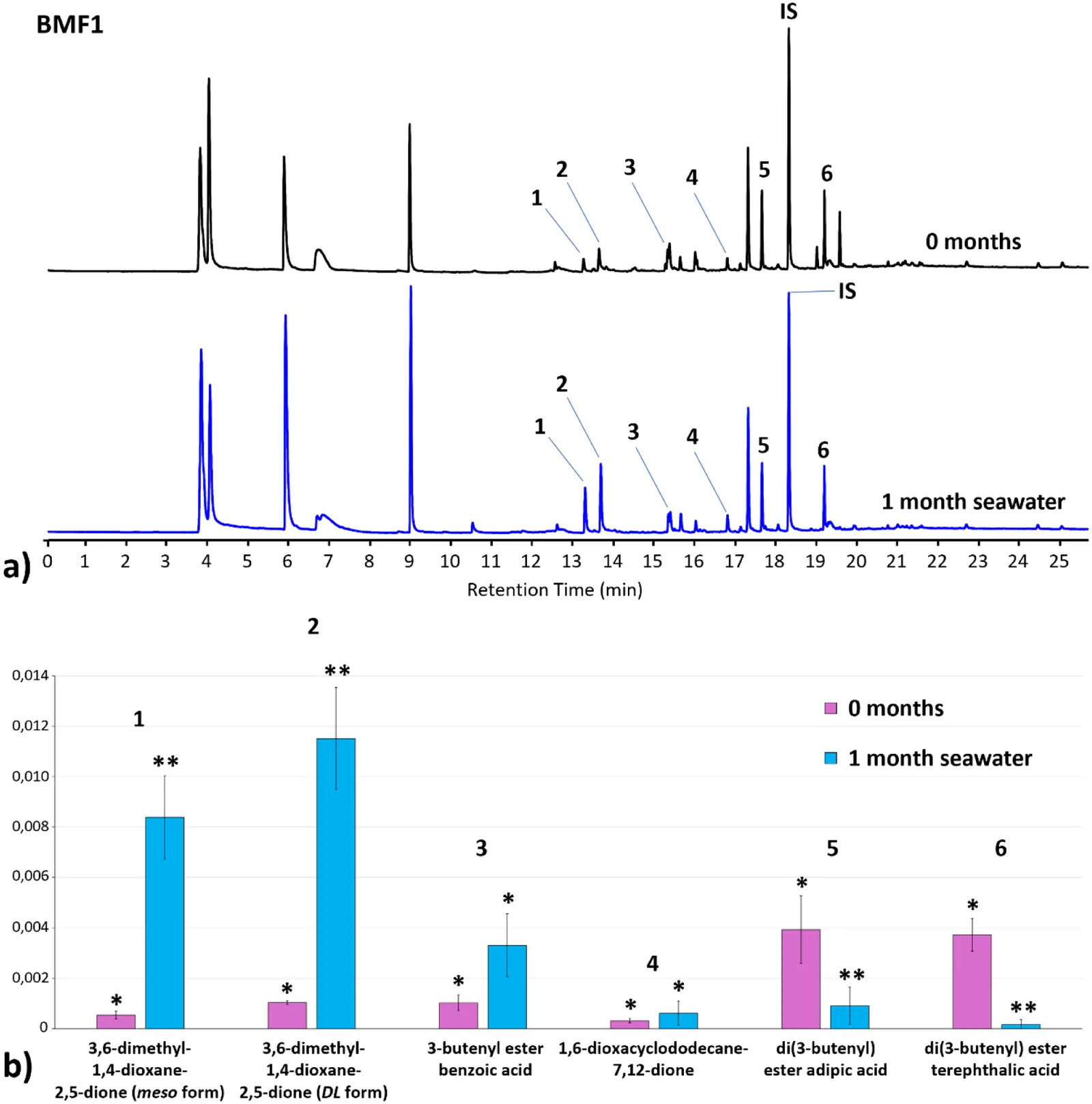
**a)** Py-GC-MS chromatograms at 600 °C of unexposed and seawater-exposed for 1 month samples of BMF1; **b)** peak areas integrated for replicate analyses (*n* = 3) and normalized by sample weight and the peak area of the internal standard, corresponding to the pyrolysis markers for unexposed and seawater-exposed samples of PLA (lactide isomers *meso*- and *DL*-3,6-dimethyl-1,4-dioxane-2,5-dione) and PBAT (3-butenyl ester benzoic acid, 1,6-dioxacyclododecane-7,12-dione, di­(3-butenyl) ester adipic acid, and di­(3-butenyl) ester terephthalic acid). Numbers from 1 to 6 indicate the correspondence between peaks in the Py-GC-MS chromatograms and the related markers. Different symbols (*, **) indicate significant differences between paired samples. The internal standard peak is indicated with the label IS.

For the mulch films exposed to soil and air and pre-exposed, the normalized peak area of the lactide *meso* form after 12 months showed a statistically significant increase with respect to the unexposed sample (Figure S32a and Table S11 in the SI, ANOVA-SNK: F­(3,8) = 12.44, p = 0.00). In the case of the lactide *DL* form as well, the peak areas integrated for all samples exposed for 12 months were significantly higher than the areas obtained for the unexposed samples, and a significant difference was also found between the pre-exposed samples and those exposed to soil and air (Figure S32a and Table S12 in the SI, ANOVA-SNK: F­(3,8) = 12.67, p = 0.00). This indicates a different, possibly faster, degradation pattern occurring under pre-exposure conditions, as already highlighted by complementary techniques as discussed above.

Regarding the PBAT markers, significant differences were only detected for 1,6-dioxacyclododecane-7,12-dione, whose normalized peak areas for all the 12 month exposed samples were significantly higher than the average for the unexposed ones, with no differences across the various exposure conditions (Figure S32b and Table S13 in the SI, ANOVA-SNK: F­(3,8) = 4.65, p = 0.04).

Moving on to BMF2, a similar statistical analysis was performed on the normalized areas of the peaks corresponding to PLA, PBAT, starch, and degradation products (normalized areas reported in Figure S33 in the SI), but no significant differences were identified between exposed and unexposed samples.

### 3.6 Study Implications

To the best of our knowledge, our work provides the first direct examination of the extent of degradation of mulch films across multiple environmental conditions. The only environmental condition in which the samples of the two films labeled as “biodegradable” disappeared from the test rigs was in seawater (and this occurred after as little as 1 month of exposure), although additional seawater experiments showed that this outcome was seasonally dependent. Therefore, this outcome should be interpreted cautiously, as material loss alone does not necessarily demonstrate biodegradation. This latter can only be evaluated by respirometry, measuring the CO_2_ evolution of or the consumption of O_2_ of the material,^57^ while material loss may instead result from fragmentation or dispersion. Since our experiments aimed at collecting environmentally relevant degradation data in the open environment, standard biodegradation test methodologies (i.e., respirometry) were not feasible.

The suite of chemical analyses applied in this study provided a detailed understanding of the physicochemical degradation of the materials, showing that both biodegradable mulch films, BMF1 and BMF2, did not reach 90% mineralization in 24 months in air, soil, and pre-exposure conditions but they showed signs of physical degradation and chemical changes were observed. A limitation of our study was that we could investigate only two commercial mulch films, which cannot be considered representative of the entire market of products labeled as “biodegradable”. However, these films are blends of the main biodegradable polymers currently used in commercial mulch films (i.e., PLA, PBAT, and thermoplastic starch).^13^ Therefore, while our results cannot be used to assess compliance with EN 17033 or to draw general conclusions about all mulch films labeled as “biodegradable” on the market, they raise reasonable concerns regarding the discrepancy between degradation observed under environmental conditions and the predictions of laboratory-based biodegradability tests for mulch films with chemical compositions and characteristics similar to those investigated in this study. Similar concerns about the validity of standard EN 10773 were already raised by Hayes and Flury^18^ including (1) limited transferability of standardized laboratory test results to field conditions, (2) form of the tested material (the standard allowed testing the material while not in its final form, e.g., in powder), and (3) limited representativeness of agricultural soil conditions. Testing the material in its final form is crucial for the purpose of a meaningful assessment of biodegradation. Since the biodegradation rate depends on the surface area accessible to microbial activity,^58^ testing the material in powder form with a greater surface area than in films can result in a faster biodegradation rate. Current biodegradation tests consider the intrinsic biodegradability of the material, testing it in optimal conditions that allow microbial development and maintain a reasonable duration of the laboratory tests. However, the assessment of the fate of biodegradable plastic in the real environment cannot adequately be deduced from laboratory conditions, and other approaches need to be followed, including studying degradation under environmentally realistic and variable conditions to understand the influence of different parameters and environmental factors.^59^

Our study further indicates that any degradation assessment should take into account different ecosystems (soil, water, etc.) in which the specific material can end up. This also underscores the importance of appropriate labeling of products tested for biodegradation, with clear disclosure of the end-of-life conditions required for biodegradation to occur.^60^ This study also represents the first comprehensive application of Py-GC-MS to examine the in situ degradation of mulch films under multiple field conditions. Py-GC-MS is not new to the monitoring of the degradation of biodegradable polymers^25,61–65^ among other techniques based on mass spectrometry.^66^ However, our study provides the first in-depth analysis of the pyrolysis products generated by samples exposed in four different environmental conditions, including a semiquantitative evaluation of the polymer-specific markers following normalization and statistical analysis.

Notably, Py-GC-MS proved to be able to detect the leaching of a plasticizer through comparative analysis before and after environmental exposure, demonstrating its potential for studying chemical leaching withthe advantage of analyzing the material itself and not the surrounding environment. Understanding the identity and rate of chemical leaching, along with any intermediate degradation products, is essential for evaluating the safety of biodegradable materials.

The study of the degradation by Py-GC-MS also provided valuable insights by identifying new products generated from the pyrolysis of the samples exposed to environmental conditions that were absent in the pyrolysis chromatograms of the pristine materials. In agreement with the SEC and SEM results, Py-GC-MS data indicated more pronounced degradation in the combined air pre-exposure and soil burial conditions than under separate air or soil exposure, although this comparison is influenced by the longer total exposure time of the pre-exposed samples. The semiquantitative analysis of the polymer markers revealed how their normalized peak area changes over time, reflecting the progressive alteration of the polymeric material. As a consequence, caution should be applied when using Py-GC-MS for the absolute quantification of biodegradable plastics in environmental samples^67^ since the peak areas of the markers might be influenced not only by the quantity of the polymer present in the sample but also by the degradation state of the material.

## Supplementary Material



## Notes

Full description of field deployment, sample collection, characterization techniques (SEM, ATR-FTIR, SEC, NMR, Py-GC-MS), and statistics performed; weight loss and surface area analyses: graphical and statistical results; additional seawater exposure experiments: full description of the methods and graphical results; SEM analysis: additional SEM micrographs of samples for each time point and exposure condition; SEC analysis: graphical results; ATR-FTIR analysis: ATR-FTIR spectra of samples for each time point and exposure condition and table including the vibrational band assignments; NMR analysis: table with the results and PLA content calculation formula; and Py-GC-MS analysis: Py-GC-MS chromatograms of samples for each time point and exposure condition, tables including the main pyrolysis products detected, plots of the normalized peak areas of pyrolysis markers, and results of the statistical analysis performed on the normalized peak areas of pyrolysis markers (PDF)
