# Supplementary material for: Monitoring the Environmental Degradation of Biodegradable Mulch Films Using Py-GC-MS and a Multitechnique Analytical Approach
Source: Environ Sci Technol. 2026 Oct 21;60(43):30959–71. doi: 10.1021/acs.est.6c11149 (PMC13738730; doi:10.1021/acs.est.6c11149)
Supplement: Supplementary file 1 [file es6c11149_si_001.pdf]

Monitoring the environmental degradation of biodegradable mulch films using Py-GC-MS and a multi-technique analytical approach

Francesca De Falco[1,2*], Tommaso Nacci[3], Fannie Burgevin[4], Winnie Courtene-Jones[1,5], Allicia Fullarton[1], Rachel L. Coppock[6], Ilaria Degano[3], Francesca Modugno[3], Richard C. Thompson[1]

[1] School of Biological and Marine Sciences, University of Plymouth, Drake Circus, PL4 8AA Plymouth, UK
[2] Department of Earth Sciences, University of Bayreuth, Universitätsstraße 30, 95447 Bayreuth, Germany
[3] Department of Chemistry and Industrial Chemistry, University of Pisa, via G. Moruzzi 13, 56124 Pisa, Italy
[4] Institute for Sustainability and Department of Chemistry, University of Bath, BA2 7AY Bath, UK
[5] School of Ocean Science, Bangor University, Menai Bridge, LL59 5AB, UK
[6] Marine Ecology & Society, Plymouth Marine Laboratory, Prospect Place, Plymouth PL1 3DH, UK

Table of Contents

1. Field deployment 2
2. Sample collection 5
3. Characterisation techniques 6
4. Statistics 7
5. Weight loss and surface area analyses 8
6. Additional seawater exposure experiments 22
7. SEM analysis 23
8. SEC analysis 26
9. ATR-FTIR analysis 27
10. NMR analysis 30
11. Py-GC-MS analysis 31

1. Field deployment

a) b)

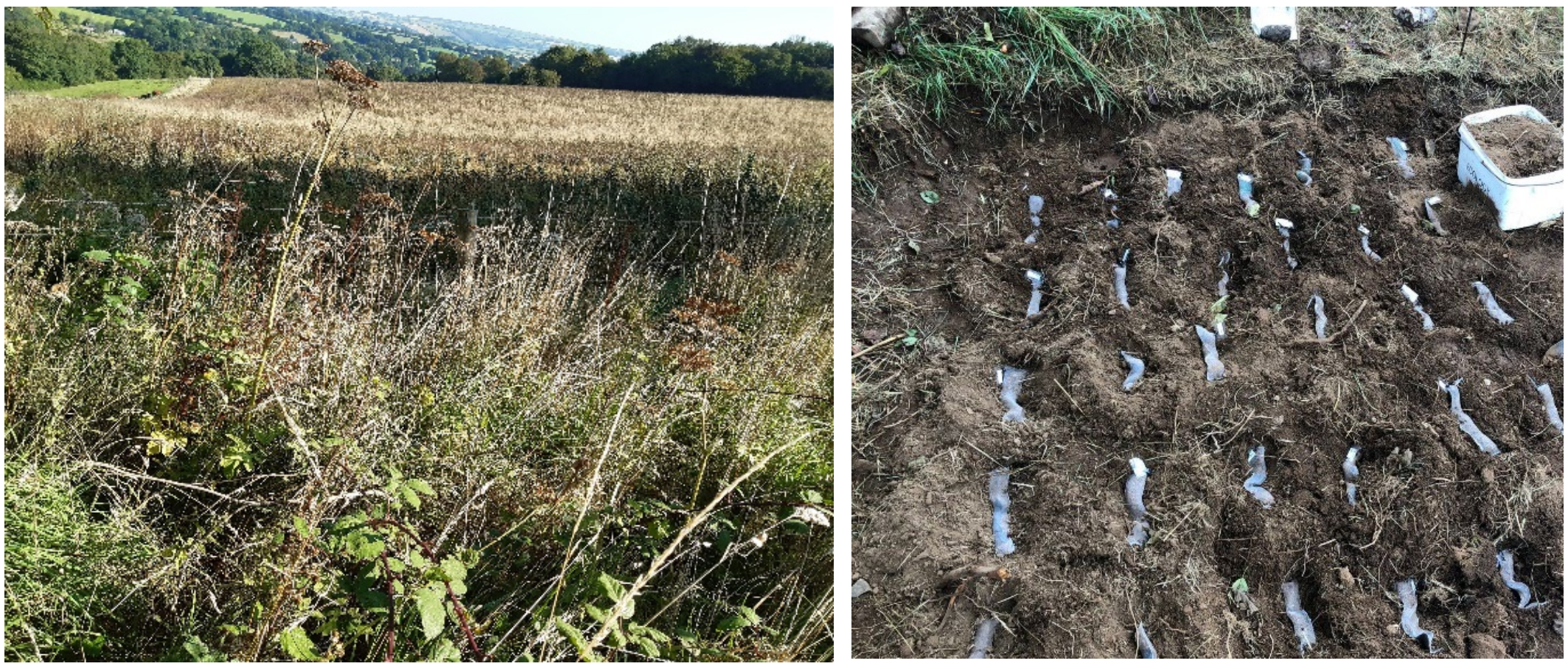

Figure S1. a) uncultivated agricultural field margin; and **b)** samples in mesh pouches prior to soil burial at a depth of 10 cm.

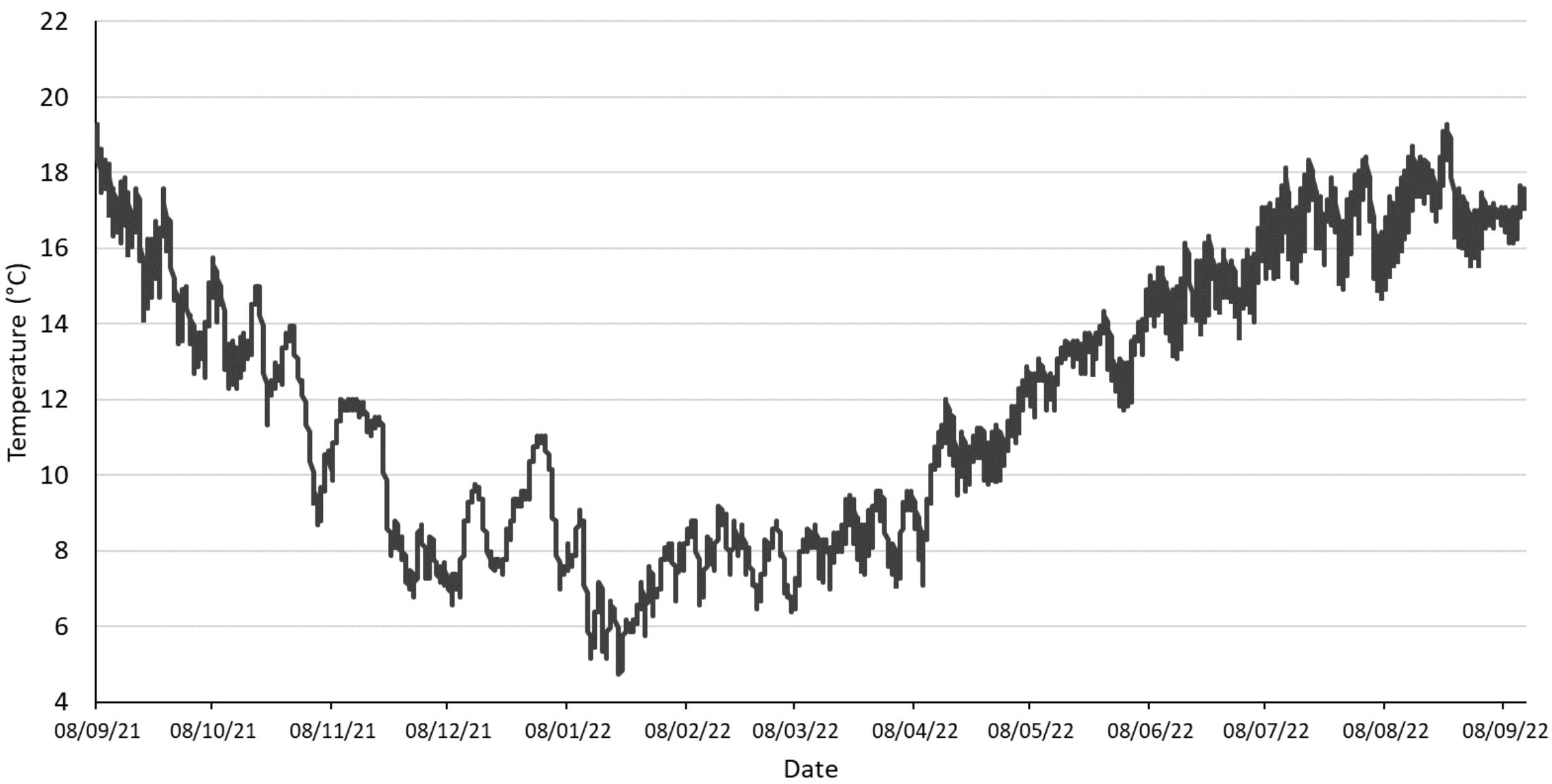

Figure S2. Soil temperature (-10 cm depth) between 9th September 2012 – 13th September 2022. Data were recorded at 2-hour intervals directly within the experimental plot in Cornwall, UK (50.53535 °N; 4.27868 °W).

Soil characteristics (Table S1) were analysed from samples taken from -10 cm depth. Soil pH was measured from a 1:2.5 soil: water slurry, using a glass combination electrode, (Corning 420). To quantify the carbon, nitrogen and C:N ratio, soil was freeze-dried, ground in a mortar with a pestle and sieved to retained particles <180 µm. Samples, in triplicate, were analysed using a CHN analyser (Vario Microcube) against a series of blanks and acetanilide standards. Carbon and nitrogen were normalised against the dry mass of samples, to obtain the values reported in Table S1.

Table S1. Characteristics of the soil of the agricultural site, measured in December 2021.

pH	**6.13**
Carbon (% mass)	4.65 ± 0.07
Carbon g/kg soil	46.54 ± 0.68
Organic Carbon (%)	5.20
Nitrogen (% mass)	0.43 ± 0.02
Nitrogen g/kg soil	4.28 ± 0.22
C:N ratio (average)	11:01

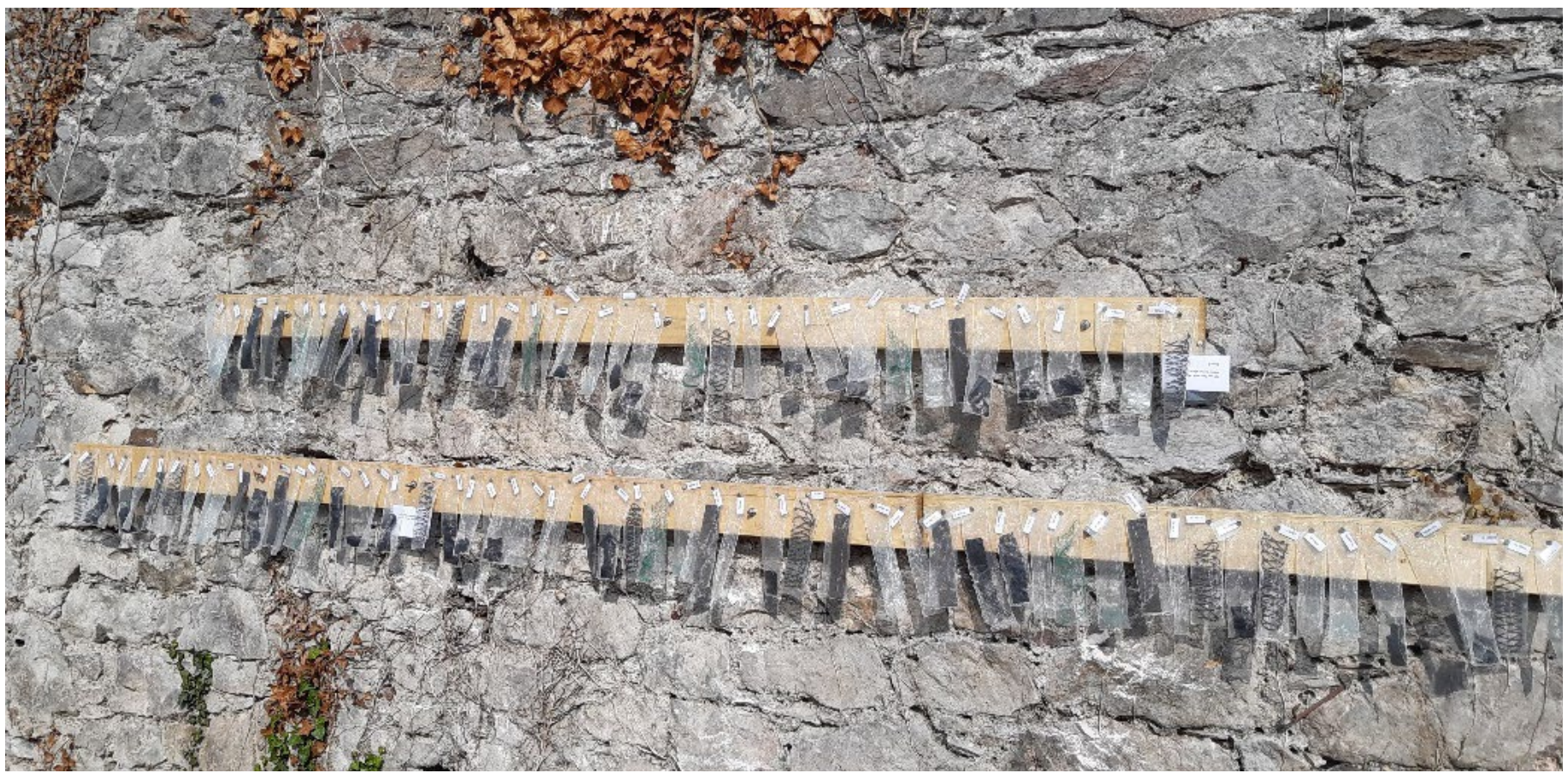

Figure S3. Samples in mesh pouches undergoing air exposure.

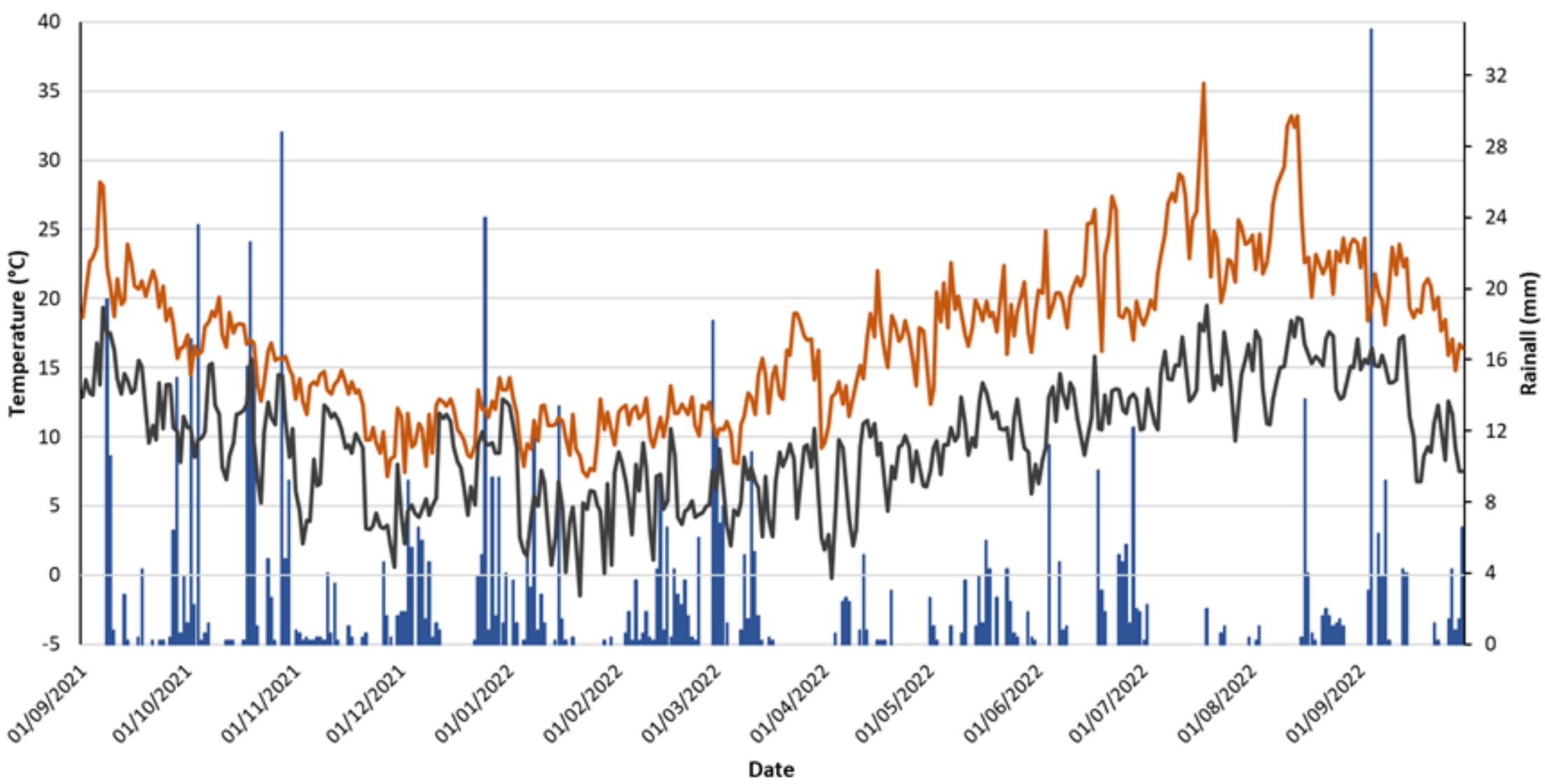

Figure S4. Daily maximum (orange line) and minimum (black line) temperatures (°C) and total rainfall (mm; blue bars) between 1st September 2021 - 30th September 2022. Data taken from weather station located in Plymouth, UK.

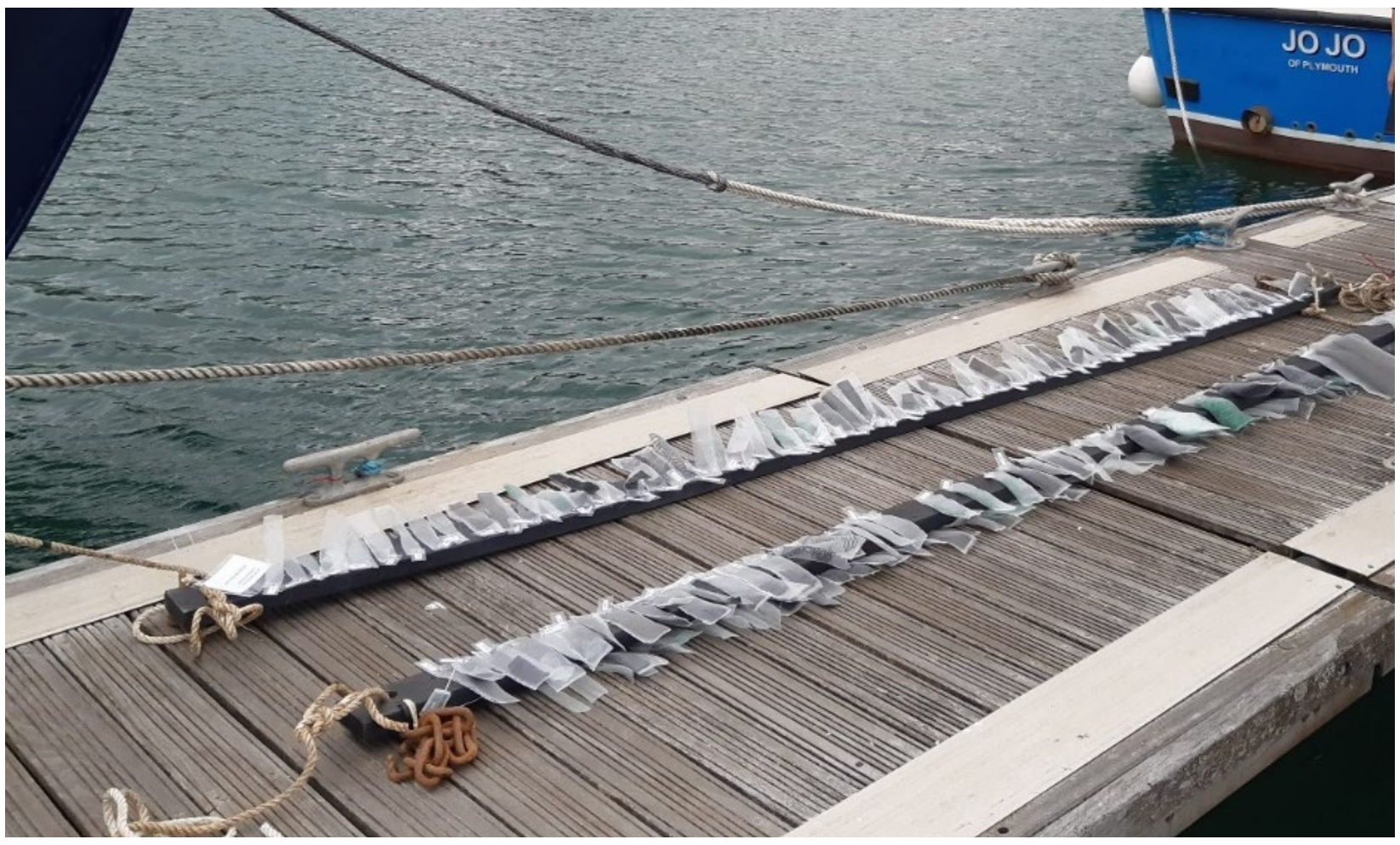

Figure S5. Samples fixed to a beam, deployed in the seawater environment a depth of 1.5 m.

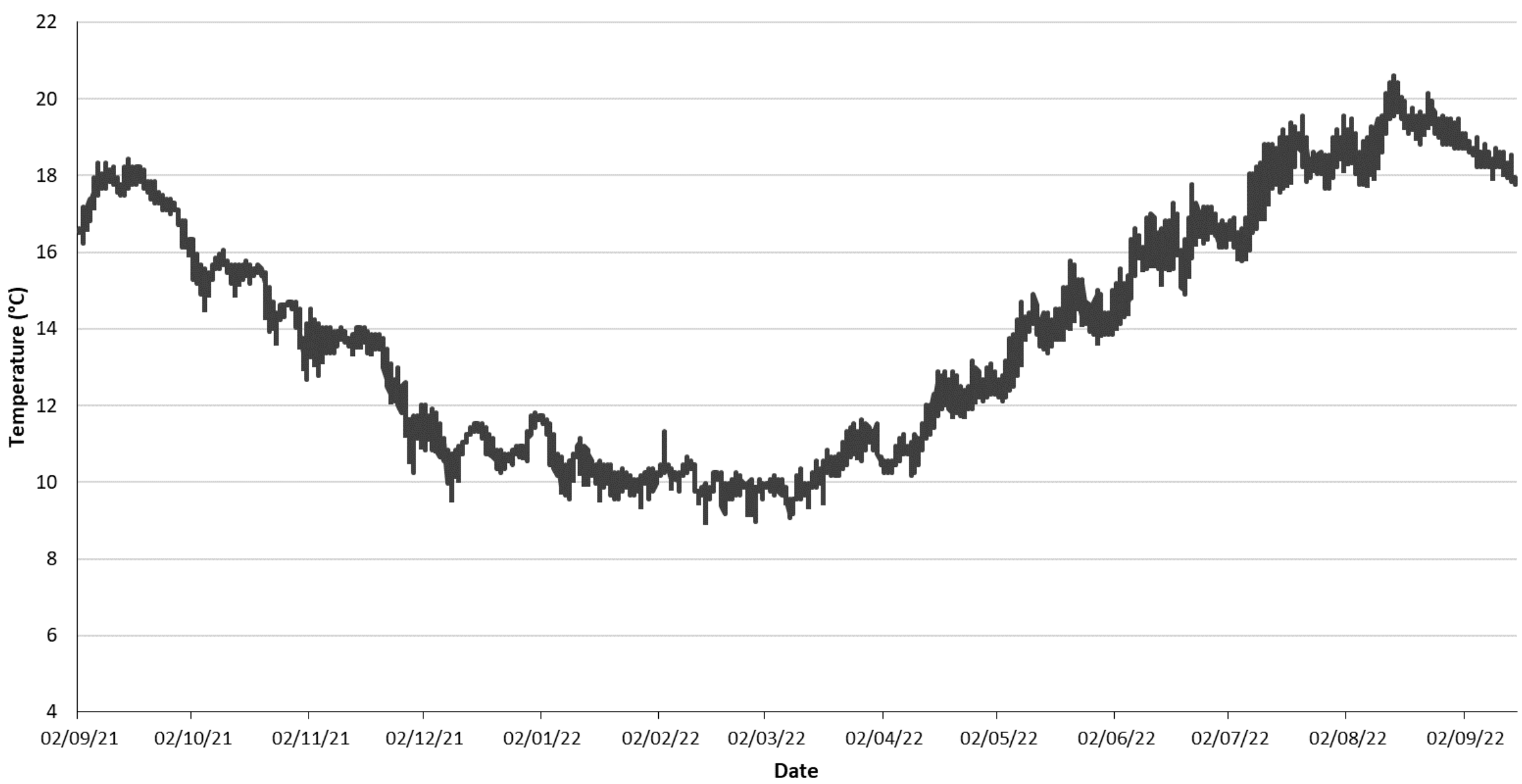

Figure S6. Seawater temperature (-1.5 m depth) between 2nd September 2021 – 15th September 2022. Data were recorded at 2-hour intervals from the experimental test rig on which samples were deployed in Plymouth, UK (50.36587 °N; 4.13164 °W).

Two additional seawater exposure experiments were conducted from 2022-2023, as detailed in Table S2. Both additional experiments were located in the same place, and at the same depth (-1.5 m) as described in the main manuscript.

Table S2. Summary details of the additional marine field experiments carried out across 2022-2023.

Experiment	Exposure Period	Deployment date	Collection dates	Temperature range (Degrees Celsius ± S.D.)
Seawater1	September-October 2022	15/09/2022	1 week (15/09/2022)<br>3 weeks (06/10/2022)<br>4 weeks (13/10/2023)<br>5 weeks (20/10/2022)	15.46 °C (±0.20 S.D.)-17.89C (±0.17 S.D.)
Seawater2	February-March 2023	21/02/2023	1 week (28/02/2023)<br>2 weeks (07/03/2023)<br>3 weeks (14/03/2023)<br>4 weeks (21/03/2023)<br>5 weeks (28/03/2023)	9.00 °C (±0.08 S.D.)-10.58°C (±0.17 S.D.)

2. Sample collection

Samples were collected on each sampling date and were placed into individual zip-lock bags to transport to the laboratory. Mesh pouches were opened by cutting along two sides of the pouch. Mulch film samples were carefully removed and placed on a 100 µm aperture stainless steel sieve. In the case of soil exposure samples, the soil was inspected to ensure all the sample had been transferred to the sieve. Samples were washed under a steady flow of deionised water to remove adhered particles/biofouling, before being dried at 40 °C until a constant weight was achieved. Samples were visually inspected for holes or disintegration, weighed (4 d.p.) and photographed to derive the surface area that was calculated using the software ImageJ (release 1.43u). Finally, samples were individually vacuum sealed in mylar bags over bagged in PE and were stored at 4 °C prior to subsequent chemical analysis.

The weight of the samples before (W_0) and after exposure (W_t) were compared to determine the weight loss (W_L) of the samples according to the equation (1) below:

$$W_L\ (\%) = 100 - \left(\frac{W_t}{W_0} \cdot 100\right) \qquad (1)$$

W_L was averaged over the triplicates of each sample collected.
The surface area of the samples before (S_0) and after exposure (S_t) were compared to determine the surface area loss (S_L) of the samples according to the equation (2) below:

$$S_L\ (\%) = 100 - \left(\frac{S_t}{S_0} \cdot 100\right) \qquad (2)$$

S_L was averaged over the triplicates of each sample collected.
Finally, samples were individually vacuum sealed in mylar bags over bagged in PE and were stored at 4 °C prior to subsequent chemical analysis.

3. Characterisation techniques

All three replicates of each time point sample underwent analysis with the techniques described below. Therefore, each replicate was subsampled according to the sample amount and dimensional requirements of the respective analytical technique. Subsampling was performed trying to select areas that appeared to be in similar degradation conditions, in order to limit possible bias. For highly degraded or fragmented samples, available fragments were selected according to the requirements of each technique.

Scanning Electron microscopy – SEM

SEM analyses were performed using a JEOL 6610 LV SEM (Tokyo, Japan). Samples were placed on stubs with adhesive tape and sputter-coated with gold prior to analysis. SEM observations were performed in high vacuum mode, with an accelerating voltage of 15 kV.

Attenuated Total Reflectance Fourier Transform Infrared spectroscopy – ATR-FTIR

The samples were analysed by ATR-FTIR through a Vertex 70 spectrometer (Bruker, Germany) with an attenuated total reflectance accessory (ATR), using 32 scans and a resolution of 4 cm^{-1}, over the range 4000–400 cm^{-1}. The obtained spectra were compared to multiple spectral databases (i.e. Bruker Optics ATR-Polymer Library, KIMW ATR-IR Polymer Library, ATR-FTIR Library Polymer, etc.).

Size Exclusion Chromatography – SEC

SEC was performed with chloroform as eluent. Samples were dissolved in chloroform (2-4 mg mL^{-1}) and were filtered through a PTFE membrane with 0.45 µm pore size before injection. An Agilent Infinity II equipped with differential refractive index (DRI) was utilised. The system was equipped with 2 x PLgel mixed C columns (300 x 7.5 mm) and a PLgel guard column. Samples were run at 1 mL min^{-1} at 40 °C. Poly(styrene) (PS) standards were used for the calibration between 195, 500 and 370 g mol^{-1}.

Nuclear Magnetic Resonance spectroscopy – NMR

^{1}H Nuclear Magnetic Resonance (NMR) spectra were recorded on a Bruker-400 MHz instrument in $CDCl_3$ at 25 °C. Chemical shift (δ) reported in ppm and the residual proton signal of the solvent was used as internal standard. A selection of samples was analysed in duplicate. To calculate the ratios of PLA/PBAT, the peak of PBAT at 8.1 ppm (that integrates for 4H) and peak at 5.1 ppm for PLA (that integrates for 1H) were used.

Pyrolysis-Gas chromatography-Mass spectrometry – Py-GC-MS

Py-GC-MS instrumentation consisted of a Multi-Shot Pyrolyzer EGA/Py-3030D micro-furnace (Frontier Laboratories Ltd., Japan) coupled to an 8890 Gas Chromatograph and a 5977 Mass Selective Detector single quadrupole mass spectrometer (Agilent Technologies, USA). The GC was equipped with a deactivated silica pre-column (2 m x 0.32 mm i.d., Agilent J&W, USA) and an HP-5MS fused silica capillary column (stationary phase 5% diphenyl-95% dimethyl-polysiloxane, 30 m x 0.25 mm i.d., 0.25 µm, Hewlett Packard, USA). Around 80 µg of each sample were placed into a clean stainless-steel cup and inserted in the microfurnace through an AS-1020E Auto-Shot sampler (Frontier Laboratories Ltd., Japan), where it was flash pyrolysed at 600 °C. Prior to analysis, 5 µL of a 200 ppm solution of

anthracene-d10 in hexane were added in the pyrolysis cup as internal standard. The injection port operated at 280 °C, with 30:1 split ratio, and the interface temperature was 280 °C. The pyrolysis products were eluted in constant flow mode at 1.0 mL min^{-1} (carrier gas He, purity 99.995%); the chromatographic program was: 40 °C isotherm for 6 min, 20 °C min^{-1} up to 310 °C, 310 °C isotherm for 40 min. The detector operated in electron ionisation mode (EI) at 70 eV in positive mode, scanning in the 35-550 *m/z* range. The ion source and quadrupole analyser temperatures were 230 °C and 150 °C, respectively. The collected data were processed by MassHunter Workstation (version 10.0, Agilent Technologies) and comparison with mass spectral databases and in-house databases built by analysing reference materials were used for peak assignment taking into account mass fragmentation and retention times. In particular, AMDIS (Automated Mass Spectral Deconvolution and Identification System) software and the NIST Mass Spectral Search Program (version 2.4) were used for peak deconvolution and integration.

4. Statistics

Statistical analyses were performed using IBM® SPSS® Statistics on weight loss, surface loss, and the normalised peak areas of pyrolysis products. Data were assessed for normality using the Shapiro-Wilk test and for homogeneity of variance using Levene's test. For the analysis of normalised pyrolysis peak areas, independent-samples t-tests were used when comparing two groups, whereas one-way analysis of variance (ANOVA) followed by a Student-Newman-Keuls (SNK) post hoc test was applied when comparing more than two groups.

For weight and surface loss data, differences among exposure times were assessed separately for each environmental exposure condition using one-way ANOVA. The initial time point (0 months) was not included in the ANOVA because weight and surface loss at the initial condition are equal to 0% by definition, as calculated according to equations (1) and (2). Therefore, this time point does not represent an experimentally variable group. Including it in the ANOVA would introduce a group with zero within-group variance, which does not reflect experimental variability and could affect the assumptions of the test. For this reason, ANOVA was performed only on the experimentally measured post-exposure time points, followed by the SNK post hoc test. When the assumption of homogeneity of variance was not met, Welch's ANOVA followed by the Games–Howell post hoc test was used.

The initial condition (0 months) was instead used as the theoretical baseline for separate one-sample t-tests comparing weight and surface loss values at each time point against 0%. When multiple time points were tested within the same environmental exposure condition, p-values were adjusted (reported as p_{adj}) using the Holm-Bonferroni correction. A significance level of 5% was used for all statistical tests; results were considered statistically significant at ($p < 0.05$), or ($p_{adj} < 0.05$) when Holm-Bonferroni correction was applied.

5. Weight loss and surface area analyses

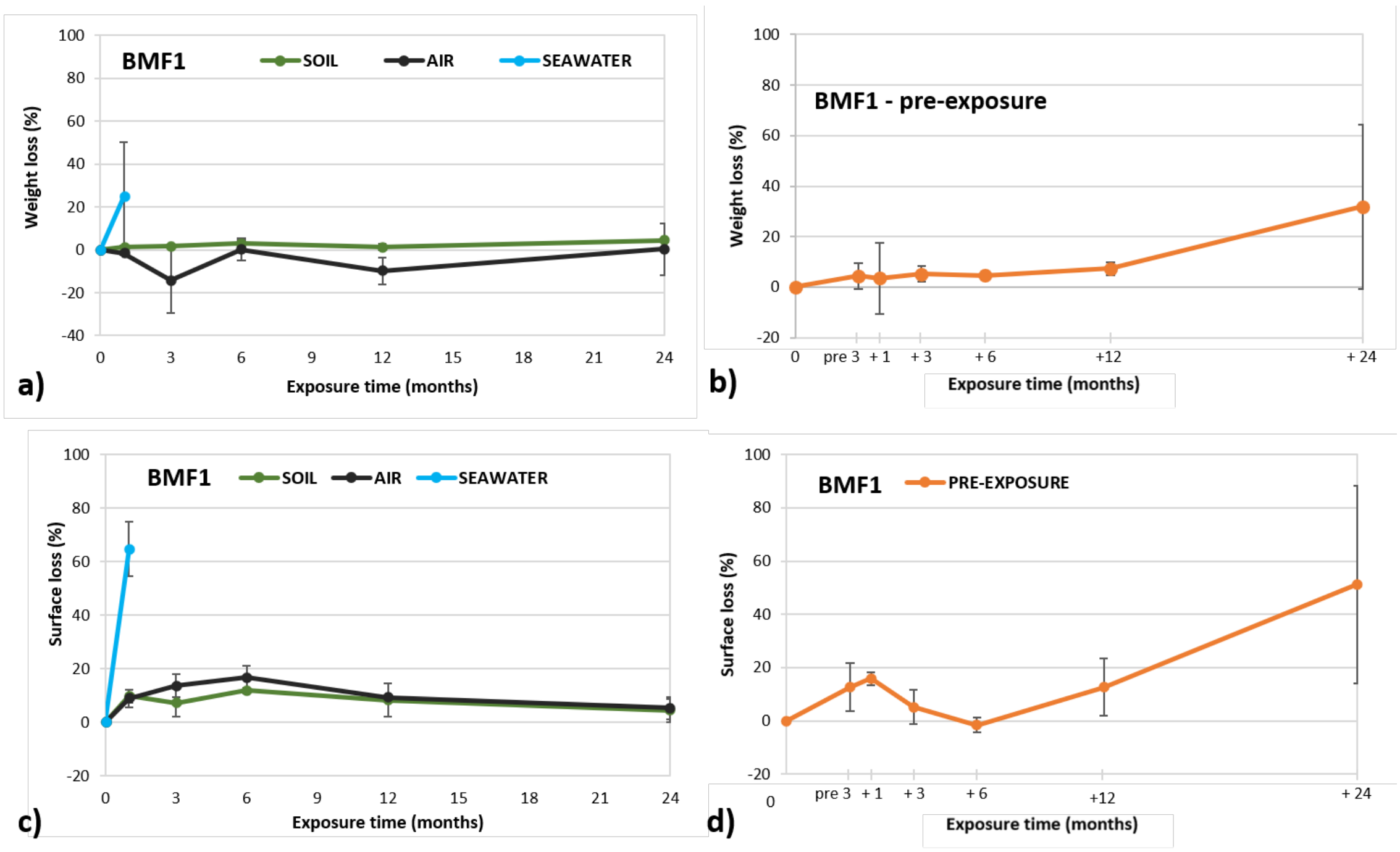

Figure S7. Plots for BMF1 reporting: weight loss of **a)** soil, air, seawater and **b)** pre-exposure samples; surface loss of **c)** soil, air, seawater and **d)** pre-exposure samples (here the exposure time is indicated as 3 months pre-exposure + 1, 3, 6, 12 and 24 months of burial).

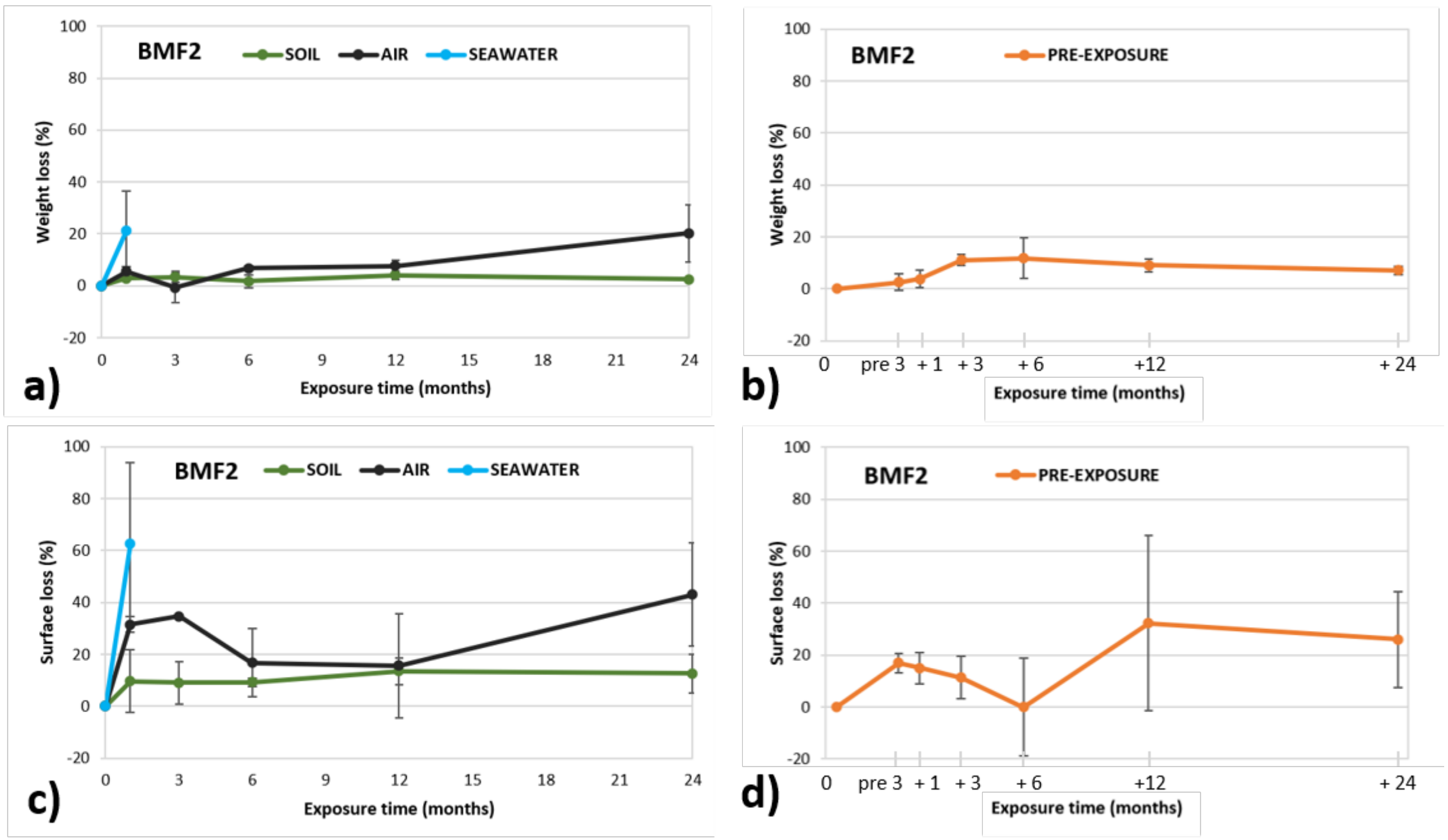

Figure S8. Plots for BMF2 reporting: weight loss of **a)** soil, air, seawater and **b)** pre-exposure samples; surface loss of **c)** soil, air, seawater and **d)** pre-exposure samples (here the exposure time is indicated as 3 months pre-exposure + 1, 3, 6, 12 and 24 months of burial).

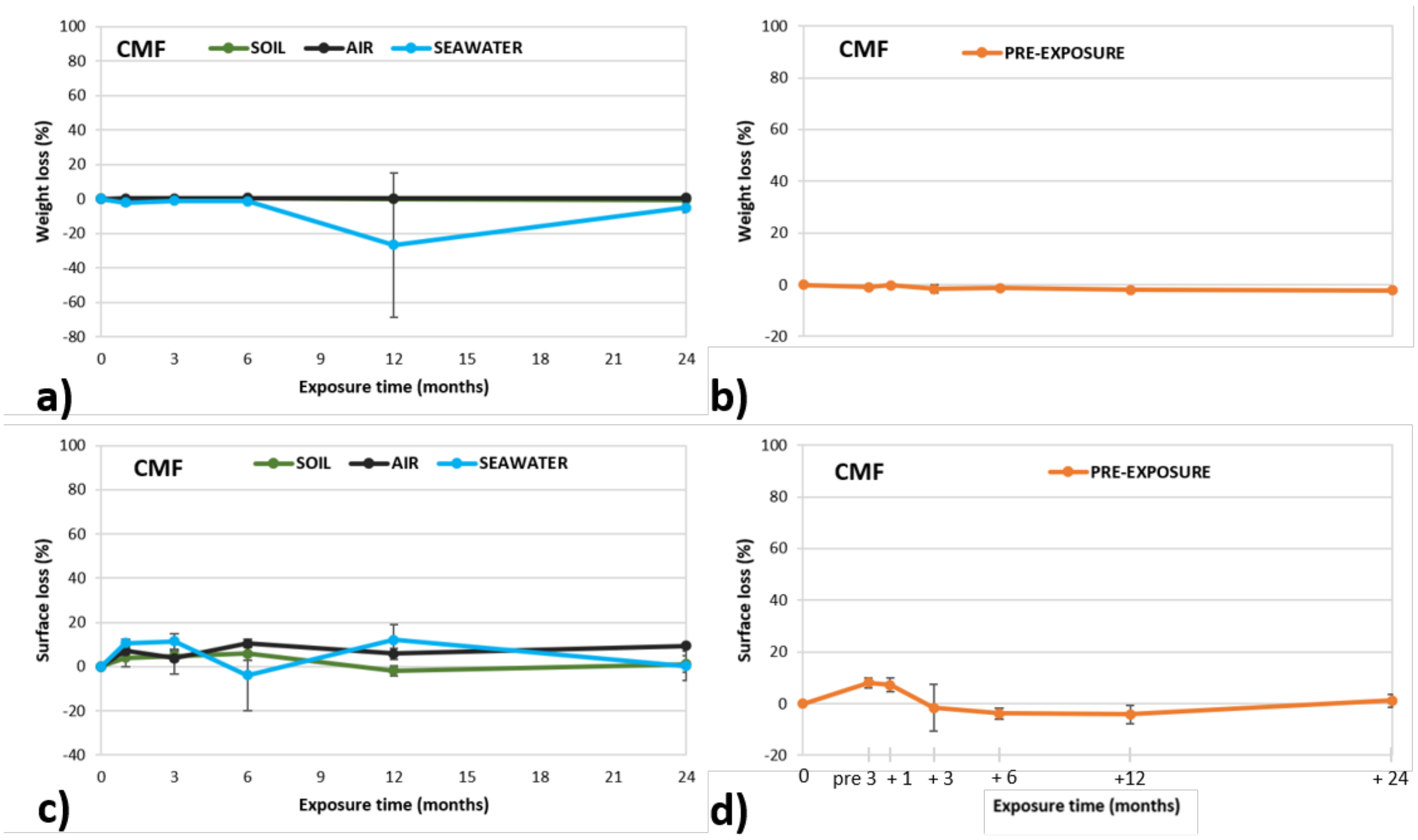

Figure S9. Plots for CMF reporting: weight loss of **a)** soil, air, seawater and **b)** pre-exposure samples; surface loss of **c)** soil, air, seawater and **d)** pre-exposure samples (here the exposure time is indicated as 3 months pre-exposure + 1, 3, 6, 12 and 24 months of burial).

Table S3. Statistics performed on weight and surface loss data for BMF1 in **a)** soil, **b)** air, **c)** seawater, **d)** and **e)** pre-exposure conditions.

ANOVA

		Sum of Squares	df	Mean Square	F	Sig.
Weight loss	Between Groups	24.507	4	6.127	5.345	.014
	Within Groups	11.462	10	1.146		
	Total	35.969	14			
Surface loss	Between Groups	93.850	4	23.463	1.310	.331
	Within Groups	179.079	10	17.908		
	Total	272.929	14			

Weight loss

Student-Newman-Keuls[a]

Exposure time	N	Subset for alpha = 0.05	
		1	2
1 m	3	1.1589333	
12 m	3	1.2322333	
3 m	3	1.7462000	
6 m	3	2.9836667	2.9836667
24 m	3		4.5264333
Sig.		.222	.108

Means for groups in homogeneous subsets are displayed.

a. Uses Harmonic Mean Sample Size = 3.000.

One-Sample Test[a]

Exposure time		Test Value = 0					
		t	df	Sig. (2-tailed)	Mean Difference	95% Confidence Interval of the Difference	
						Lower	Upper
1 m	Weight loss	3.079	2	.091	1.15893333	-.4604822	2.7783489
	Surface loss	7.055	2	.020	9.73874	3.7997	15.6777
3 m	Weight loss	9.551	2	.011	1.74620000	.9595868	2.5328132
	Surface loss	2.500	2	.130	7.30170	-5.2625	19.8659
6 m	Weight loss	3.469	2	.074	2.98366667	-.7173272	6.6846605
	Surface loss	20.292	2	.002	11.93653	9.4055	14.4676
12 m	Weight loss	1.442	2	.286	1.23223333	-2.4451037	4.9095703
	Surface loss	2.282	2	.150	8.19082	-7.2507	23.6324
24 m	Weight loss	8.794	2	.013	4.52643333	2.3117772	6.7410894
	Surface loss	1.781	2	.217	4.42929	-6.2741	15.1327

a. No statistics are computed for one or more split files

Holm-Bonferroni correction

Weight loss		
**Exposure time**	**Raw p value**	**Adjusted p value**
1 m	0.091	0.273
3 m	0.011	0.055
6 m	0.74	0.740
12 m	0.286	0.572
24 m	0.013	0.055

Surface loss		
**Exposur e time**	**Raw p value**	**Adjusted p value**
1 m	0.020	0.080
3 m	0.130	0.390
6 m	0.002	0.010
12 m	0.150	0.390
24 m	0.217	0.390

a)

Test of Homogeneity of Variances

	Levene Statistic	df1	df2	Sig.
Weight loss	4.885	4	10	.019
Surface loss	1.799	4	10	.206

ANOVA

		Sum of Squares	df	Mean Square	F	Sig.
Weight loss	Between Groups	524.353	4	131.088	1.502	.274
	Within Groups	872.777	10	87.278		
	Total	1397.130	14			
Surface loss	Between Groups	237.472	4	59.368	4.537	.024
	Within Groups	130.849	10	13.085		
	Total	368.320	14			

Robust Tests of Equality of Means

		Statistic[a]	df1	df2	Sig.
Weight loss	Welch	1.245	4	4.057	.417
Surface loss	Welch	2.517	4	4.260	.189

a. Asymptotically F distributed.

Surface loss

	Exposure time	N	Subset for alpha = 0.05	
			1	2
Student-Newman-Keuls[a]	24 m	3	5.3333	
	1 m	3	8.9735	8.9735
	12 m	3	9.3764	9.3764
	3 m	3	13.6388	13.6388
	6 m	3		16.7878
	Sig.		.074	.096

Means for groups in homogeneous subsets are displayed.

a. Uses Harmonic Mean Sample Size = 3.000.

One-Sample Test

Exposure time		Test Value = 0					
		t	df	Sig. (2-tailed)	Mean Difference	95% Confidence Interval of the Difference Lower	Upper
1 m	Weight loss	-4.551	2	.045	-1.67100000	-3.2509018	-.0910982
	Surface loss	4.723	2	.042	8.97346	.7987	17.1482
3 m	Weight loss	-1.645	2	.242	-14.26833333	-51.5996258	23.0629591
	Surface loss	5.490	2	.032	13.63879	2.9500	24.3276
6 m	Weight loss	.066	2	.954	.19766667	-12.7641957	13.1595291
	Surface loss	6.891	2	.020	16.78779	6.3052	27.2704
12 m	Weight loss	-2.669	2	.116	-9.78166667	-25.5524509	5.9891176
	Surface loss	21.080	2	.002	9.37641	7.4626	11.2902
24 m	Weight loss	.041	2	.971	.28500000	-29.3811230	29.9511230
	Surface loss	2.197	2	.159	5.33335	-5.1120	15.7786

Holm-Bonferroni correction

Weight loss			Surface loss		
Exposure time	Raw p value	Adjusted p value	Exposure time	Raw p value	Adjusted p value
1 m	0.045	0.225	1 m	0.042	0.096
3 m	0.242	0.726	3 m	0.032	0.096
6 m	0.954	1.000	6 m	0.020	0.080
12 m	0.110	0.440	12 m	0.002	0.010
24 m	0.971	1.000	24 m	0.159	0.159

b)

c)

One-Sample Test

	Test Value = 0					
					95% Confidence Interval of the Difference	
	t	df	Sig. (2-tailed)	Mean Difference	Lower	Upper
Weight loss	1.692	2	.233	24.72820000	-38.1695374	87.6259374
Surface loss	11.067	2	.008	64.66814	39.5265	89.8097

Test of Homogeneity of Variances

	Levene Statistic	df1	df2	Sig.
Weight loss	4.913	5	12	.011
Surface loss	3.251	5	12	.044

Robust Tests of Equality of Means

		Statistic[a]	df1	df2	Sig.
Weight loss	Welch	.629	5	5.325	.688
Surface loss	Welch	9.619	5	5.322	.011

a. Asymptotically F distributed.

Multiple Comparisons

Dependent Variable: Surface loss

Games-Howell

(I) Exposure time	(J) Exposure time	Mean Difference (I-J)	Std. Error	Sig.	95% Confidence Interval Lower Bound	95% Confidence Interval Upper Bound
3 m pre	1 m	-3.16281	5.47221	.984	-42.3190	35.9934
	3 m	7.47243	6.42337	.834	-25.0813	40.0262
	6 m	14.28688	5.51527	.354	-24.0873	52.6611
	12 m	.00910	8.15101	1.000	-39.1817	39.1999
	24 m	-38.56467	22.11036	.606	-200.5628	123.4335
1 m	3 m pre	3.16281	5.47221	.984	-35.9934	42.3190
	3 m	10.63524	3.92920	.314	-14.3767	35.6472
	6 m	17.44969*	2.14408	.008	7.2244	27.6750
	12 m	3.17192	6.37321	.991	-44.1269	50.4707
	24 m	-35.40186	21.51853	.648	-212.1144	141.3107
3 m	3 m pre	-7.47243	6.42337	.834	-40.0262	25.0813
	1 m	-10.63524	3.92920	.314	-35.6472	14.3767
	6 m	6.81445	3.98894	.607	-17.5628	31.1917
	12 m	-7.46332	7.20641	.882	-46.2671	31.3405
	24 m	-46.03709	21.77985	.490	-215.6096	123.5355
6 m	3 m pre	-14.28688	5.51527	.354	-52.6611	24.0873
	1 m	-17.44969*	2.14408	.008	-27.6750	-7.2244
	3 m	-6.81445	3.98894	.607	-31.1917	17.5628
	12 m	-14.27777	6.41022	.450	-60.8042	32.2487
	24 m	-52.85154	21.52952	.408	-229.2406	123.5375
12 m	3 m pre	-.00910	8.15101	1.000	-39.1999	39.1817
	1 m	-3.17192	6.37321	.991	-50.4707	44.1269
	3 m	7.46332	7.20641	.882	-31.3405	46.2671
	6 m	14.27777	6.41022	.450	-32.2487	60.8042
	24 m	-38.57377	22.35041	.610	-195.9265	118.7790
24 m	3 m pre	38.56467	22.11036	.606	-123.4335	200.5628
	1 m	35.40186	21.51853	.648	-141.3107	212.1144
	3 m	46.03709	21.77985	.490	-123.5355	215.6096
	6 m	52.85154	21.52952	.408	-123.5375	229.2406
	12 m	38.57377	22.35041	.610	-118.7790	195.9265

d)

*. The mean difference is significant at the 0.05 level.

One-Sample Test

Exposure time		Test Value = 0					
		t	df	Sig. (2-tailed)	Mean Difference	95% Confidence Interval of the Difference	
						Lower	Upper
3 m pre	Weight loss	1.500	2	.272	4.36210333	-8.1495254	16.8737320
	Surface loss	2.399	2	.139	12.66829	-10.0516	35.3882
1 m	Weight loss	.428	2	.711	3.43866333	-31.1703901	38.0477167
	Surface loss	11.025	2	.008	15.83110	9.6527	22.0095
3 m	Weight loss	2.860	2	.104	5.22344667	-2.6338645	13.0807578
	Surface loss	1.421	2	.291	5.19586	-10.5407	20.9324
6 m	Weight loss	4.891	2	.039	4.59081333	.5519999	8.6296268
	Surface loss	-1.017	2	.416	-1.61858	-8.4692	5.2321
12 m	Weight loss	5.185	2	.035	7.29085333	1.2409495	13.3407572
	Surface loss	2.039	2	.178	12.65919	-14.0574	39.3758
24 m	Weight loss	1.693	2	.233	31.85855000	-49.1172356	112.8343356
	Surface loss	2.386	2	.140	51.23296	-41.1474	143.6133

Holm-Bonferroni correction

e)

Weight loss		
**Exposure time**	**Raw p value**	**Adjusted p value**
3 m pre	0.272	0.699
1 m	0.711	0.711
3 m	0.104	0.416
6 m	0.039	0.195
12 m	0.035	0.210
24 m	0.233	0.699

Surface loss		
**Exposure time**	**Raw p value**	**Adjusted p value**
3 m pre	0.139	0.695
1 m	0.008	0.048
3 m	0.291	0.695
6 m	0.416	0.695
12 m	0.178	0.695
24 m	0.140	0.695

Table S4. Statistics performed on weight and surface loss data for BMF2 in **a)** soil, **b)** air, **c)** seawater, **d)** pre-exposure conditions.

ANOVA

		Sum of Squares	df	Mean Square	F	Sig.
Weight loss	Between Groups	9.557	4	2.389	.816	.543
	Within Groups	29.265	10	2.926		
	Total	38.822	14			
Surface loss	Between Groups	53.275	4	13.319	.226	.918
	Within Groups	590.308	10	59.031		
	Total	643.583	14			

One-Sample Test

		Test Value = 0					
						95% Confidence Interval of the Difference	
Exposure time		t	df	Sig. (2-tailed)	Mean Difference	Lower	Upper
1 m	Weight loss	34.516	2	.001	2.86796667	2.5104571	3.2254762
	Surface loss	1.393	2	.298	9.63443	-20.1190	39.3879
3 m	Weight loss	2.700	2	.114	3.37783333	-2.0046895	8.7603561
	Surface loss	1.904	2	.197	9.01353	-11.3580	29.3851
6 m	Weight loss	1.240	2	.341	1.74216667	-4.3009991	7.7853324
	Surface loss	9.497	2	.011	9.17434	5.0180	13.3307
12 m	Weight loss	4.300	2	.050	4.11483333	-.0020638	8.2317304
	Surface loss	4.492	2	.046	13.52164	.5695	26.4738
24 m	Weight loss	3.903	2	.060	2.52166667	-.2580454	5.3013787
	Surface loss	2.952	2	.098	12.57750	-5.7548	30.9098

Holm-Bonferroni correction

Weight loss			Surface loss		
Exposure time	Raw p value	Adjusted p value	Exposure time	Raw p value	Adjusted p value
1 m	0.001	0.005	1 m	0.298	0.394
3 m	0.114	0.228	3 m	0.197	0.394
6 m	0.341	0.341	6 m	0.011	0.055
12 m	0.050	0.200	12 m	0.046	0.184
24 m	0.060	0.200	24 m	0.098	0.294

a)

Test of Homogeneity of Variances

	Levene Statistic	df1	df2	Sig.
Weight loss	4.375	4	10	.027
Surface loss	4.976	4	10	.018

Robust Tests of Equality of Means

		Statistic[a]	df1	df2	Sig.
Weight loss	Welch	2.069	4	4.500	.235
Surface loss	Welch	1.972	4	4.069	.261

a. Asymptotically F distributed.

One-Sample Test

		Test Value = 0					
						95% Confidence Interval of the Difference	
Exposure time		t	df	Sig. (2-tailed)	Mean Difference	Lower	Upper
1 m	Weight loss	5.241	2	.035	5.44920000	.9755444	9.9228556
	Surface loss	18.556	2	.003	31.52304	24.2138	38.8322
3 m	Weight loss	-.239	2	.833	-.77770000	-14.7878688	13.2324688
	Surface loss	111.760	2	.000	34.60085	33.2687	35.9330
6 m	Weight loss	14.299	2	.005	6.83160000	4.7759379	8.8872621
	Surface loss	2.189	2	.160	16.71507	-16.1396	49.5697
12 m	Weight loss	6.157	2	.025	7.64783333	2.3036183	12.9920484
	Surface loss	1.337	2	.313	15.59238	-34.5781	65.7629
24 m	Weight loss	3.199	2	.085	20.31283333	-7.0097335	47.6354002
	Surface loss	3.723	2	.065	43.05951	-6.7104	92.8294

Holm-Bonferroni correction

b)

Weight loss			Surface loss		
**Exposure time**	**Raw p value**	**Adjusted p value**	**Exposure time**	**Raw p value**	**Adjusted p value**
1 m	0.035	0.105	1 m	0.003	0.012
3 m	0.833	0.833	3 m	0.000	0.000
6 m	0.005	0.025	6 m	0.160	0.320
12 m	0.025	0.100	12 m	0.313	0.320
24 m	0.085	0.170	24 m	0.065	0.195

c)

One-Sample Test

	Test Value = 0					
					95% Confidence Interval of the Difference	
	t	df	Sig. (2-tailed)	Mean Difference	Lower	Upper
Weight loss	2.443	2	.135	21.27103333	-16.1974473	58.7395139
Surface loss	3.444	2	.075	62.52016	-15.5953	140.6356

Test of Homogeneity of Variances

	Levene Statistic	df1	df2	Sig.
Weight loss	4.072	5	12	.022
Surface loss	3.062	5	12	.052

ANOVA

		Sum of Squares	df	Mean Square	F	Sig.
Weight loss	Between Groups	215.270	5	43.054	2.618	.080
	Within Groups	197.329	12	16.444		
	Total	412.598	17			
Surface loss	Between Groups	1078.658	5	215.732	.664	.658
	Within Groups	3897.206	12	324.767		
	Total	4975.864	17			

Robust Tests of Equality of Means

		Statistic[a]	df1	df2	Sig.
Weight loss	Welch	2.776	5	5.470	.133
Surface loss	Welch	.391	5	5.295	.838

a. Asymptotically F distributed.

One-Sample Test

		Test Value = 0					
						95% Confidence Interval of the Difference	
Exposure time		t	df	Sig. (2-tailed)	Mean Difference	Lower	Upper
3 m pre	Weight loss	1.344	2	.311	2.52963333	-5.5689083	10.6281750
	Surface loss	7.553	2	.017	16.91065	7.2771	26.5442
1 m	Weight loss	1.888	2	.200	3.79303333	-4.8488183	12.4348850
	Surface loss	4.345	2	.049	14.99807	.1470	29.8492
3 m	Weight loss	8.867	2	.012	11.08286667	5.7047583	16.4609750
	Surface loss	2.368	2	.141	11.35556	-9.2735	31.9846
6 m	Weight loss	2.583	2	.123	11.74330000	-7.8171133	31.3037133
	Surface loss	1.069	2	.397	11.64585	-35.2104	58.5021
12 m	Weight loss	6.028	2	.026	9.02263333	2.5822288	15.4630379
	Surface loss	1.664	2	.238	32.30159	-51.2218	115.8250
24 m	Weight loss	7.740	2	.016	7.09803333	3.1519972	11.0440695
	Surface loss	2.438	2	.135	26.05745	-19.9222	72.0371

Holm-Bonferroni correction

Weight loss		
**Exposure time**	**Raw p value**	**Adjusted p value**
3 m pre	0.311	0.400
1 m	0.200	0.400
3 m	0.012	0.072
6 m	0.123	0.369
12 m	0.026	0.104
24 m	0.016	0.080

Surface loss		
**Exposure time**	**Raw p value**	**Adjusted p value**
3 m pre	0.200	0.800
1 m	0.212	0.636
3 m	0.002	0.012
6 m	0.273	0.546
12 m	0.109	0.545
24 m	0.309	0.309

d)

Table S5. Statistics performed on weight and surface loss data for CMF in **a)** soil, **b)** air, **c)** seawater, **d)** and **e)** pre-exposure conditions.

Test of Homogeneity of Variances

	Levene Statistic	df1	df2	Sig.
Weight loss	8.465	4	9	.004
Surface loss	.952	4	9	.478

ANOVA

		Sum of Squares	df	Mean Square	F	Sig.
Weight loss	Between Groups	2.201	4	.550	1.570	.263
	Within Groups	3.156	9	.351		
	Total	5.357	13			
Surface loss	Between Groups	108.479	4	27.120	2.737	.097
	Within Groups	89.186	9	9.910		
	Total	197.665	13			

Robust Tests of Equality of Means

		Statistic[a]	df1	df2	Sig.
Weight loss	Welch	4.358	4	3.363	.113
Surface loss	Welch	2.750	4	4.000	.175

a. Asymptotically F distributed.

One-Sample Test[a]

		Test Value = 0					
						95% Confidence Interval of the Difference	
Exposure time		t	df	Sig. (2-tailed)	Mean Difference	Lower	Upper
1 m	Weight loss	-2.450	2	.134	-.88233289	-2.4320755	.6674097
	Surface loss	1.768	2	.219	3.94930	-5.6642	13.5628
3 m	Weight loss	-5.717	2	.029	-.64495051	-1.1303444	-.1595566
	Surface loss	3.593	2	.069	4.79625	-.9478	10.5403
6 m	Weight loss	.334	1	.795	.30813465	-11.4311051	12.0473744
	Surface loss	2.760	1	.221	5.81209	-20.9449	32.5691
12 m	Weight loss	-1.979	2	.186	-.07886494	-.2503642	.0926343
	Surface loss	-1.392	2	.299	-1.91760	-7.8455	4.0103
24 m	Weight loss	-1.477	2	.278	-.46106384	-1.8037470	.8816194
	Surface loss	.506	2	.663	1.09937	-8.2421	10.4409

a. No statistics are computed for one or more split files

Holm-Bonferroni correction

Weight loss			Surface loss		
Exposure time	Raw p value	Adjusted p value	Exposure time	Raw p value	Adjusted p value
1 m	0.134	0.536	1 m	0.219	0.876
3 m	0.029	0.145	3 m	0.069	0.345
6 m	0.795	0.795	6 m	0.221	0.876
12 m	0.186	0.558	12 m	0.299	0.876
24 m	0.278	0.558	24 m	0.663	0.876

a)

Test of Homogeneity of Variances

	Levene Statistic	df1	df2	Sig.
Weight loss	1.971	4	10	.175
Surface loss	2.862	4	10	.081

ANOVA

		Sum of Squares	df	Mean Square	F	Sig.
Weight loss	Between Groups	.405	4	.101	.385	.815
	Within Groups	2.633	10	.263		
	Total	3.038	14			
Surface loss	Between Groups	87.969	4	21.992	1.761	.213
	Within Groups	124.893	10	12.489		
	Total	212.862	14			

One-Sample Test

		Test Value = 0					
						95% Confidence Interval of the Difference	
Exposure time		t	df	Sig. (2-tailed)	Mean Difference	Lower	Upper
1 m	Weight loss	.499	2	.667	.10079297	-.7675472	.9691332
	Surface loss	24.485	2	.002	7.13672	5.8826	8.3908
3 m	Weight loss	.556	2	.634	.29009696	-1.9551544	2.5353483
	Surface loss	.901	2	.463	3.68890	-13.9277	21.3055
6 m	Weight loss	1.616	2	.247	.50666059	-.8422289	1.8555501
	Surface loss	9.138	2	.012	10.41579	5.5112	15.3203
12 m	Weight loss	1.928	2	.194	.28617442	-.3523989	.9247477
	Surface loss	3.737	2	.065	5.71798	-.8664	12.3023
24 m	Weight loss	7.482	2	.017	.55191399	.2345122	.8693158
	Surface loss	16.320	2	.004	9.31384	6.8583	11.7694

Holm-Bonferroni correction

b)

Weight loss		
**Exposure time**	**Raw p value**	**Adjusted p value**
1 m	0.667	1.000
3 m	0.634	1.000
6 m	0.247	0.776
12 m	0.194	0.776
24 m	0.017	0.085

Surface loss		
**Exposure time**	**Raw p value**	**Adjusted p value**
1 m	0.002	0.010
3 m	0.463	0.463
6 m	0.012	0.036
12 m	0.065	0.130
24 m	0.004	0.016

Test of Homogeneity of Variances

	Levene Statistic	df1	df2	Sig.
Weight loss	14.805	4	10	.000
Surface loss	5.599	4	10	.012

Robust Tests of Equality of Means

		Statistic[a]	df1	df2	Sig.
Weight loss	Welch	1.431	4	4.611	.354
Surface loss	Welch	1.669	4	4.473	.303

a. Asymptotically F distributed.

One-Sample Test

		Test Value = 0					
						95% Confidence Interval of the Difference	
Exposure time		t	df	Sig. (2-tailed)	Mean Difference	Lower	Upper
1 m	Weight loss	-2.155	2	.164	-2.11341933	-6.3336781	2.1068395
	Surface loss	11.148	2	.008	10.59881	6.5081	14.6895
3 m	Weight loss	-4.079	2	.055	-1.07116635	-2.2011972	.0588645
	Surface loss	5.700	2	.029	11.39758	2.7940	20.0012
6 m	Weight loss	-3.995	2	.057	-1.33076106	-2.7639550	.1024329
	Surface loss	-.418	2	.717	-3.86610	-43.6744	35.9422
12 m	Weight loss	-1.111	2	.382	-26.79450758	-130.5441290	76.9551138
	Surface loss	3.008	2	.095	11.97751	-5.1534	29.1084
24 m	Weight loss	-3.195	2	.086	-5.08027331	-11.9218079	1.7612613
	Surface loss	.059	2	.958	.23035	-16.4468	16.9075

Holm-Bonferroni correction

c)

Weight loss		
Exposure time	Raw p value	Adjusted p value
1 m	0.164	0.328
3 m	0.055	0.275
6 m	0.057	0.275
12 m	0.382	0.382
24 m	0.086	0.275

Surface loss		
Exposure time	Raw p value	Adjusted p value
1 m	0.008	0.040
3 m	0.029	0.116
6 m	0.717	1.000
12 m	0.095	0.285
24 m	0.958	1.000

Test of Homogeneity of Variances

	Levene Statistic	df1	df2	Sig.
Weight loss	5.760	5	12	.006
Surface loss	1.955	5	12	.158

ANOVA

		Sum of Squares	df	Mean Square	F	Sig.
Weight loss	Between Groups	8.700	5	1.740	2.671	.076
	Within Groups	7.816	12	.651		
	Total	16.516	17			
Surface loss	Between Groups	436.701	5	87.340	4.640	.014
	Within Groups	225.863	12	18.822		
	Total	662.563	17			

Robust Tests of Equality of Means *

		Statistic[a]	df1	df2	Sig.
Weight loss	Welch	9.707	5	5.187	.012
Surface loss	Welch	10.291	5	5.522	.009

a. Asymptotically F distributed.

Surface loss

	Exposure time	N	Subset for alpha = 0.05 1	Subset for alpha = 0.05 2
Student-Newman-Keuls[a]	12 m	3	-4.1804	
	6 m	3	-3.8669	
	3 m	3	-1.6594	-1.6594
	24 m	3	1.1586	1.1586
	1 m	3		7.1786
	3 m pre	3		8.0604
	Sig.		.463	.073

Means for groups in homogeneous subsets are displayed.

a. Uses Harmonic Mean Sample Size = 3,000.

d) *For the surface loss, Games-Howell post-hoc test detected no significant differences.

One-Sample Test

Exposure time		Test Value = 0					
		t	df	Sig. (2-tailed)	Mean Difference	95% Confidence Interval of the Difference	
						Lower	Upper
3 m pre	Weight loss	-5.061	2	.037	-.90945530	-1.6825892	-.1363214
	Surface loss	7.618	2	.017	8.06045	3.5079	12.6130
1 m	Weight loss	-2.506	2	.129	-.20018178	-.5438681	.1435045
	Surface loss	4.642	2	.043	7.17862	.5254	13.8318
3 m	Weight loss	-1.727	2	.226	-1.64476346	-5.7426312	2.4531043
	Surface loss	-.321	2	.779	-1.65939	-23.9106	20.5918
6 m	Weight loss	-6.775	2	.021	-1.26661377	-2.0710640	-.4621636
	Surface loss	-3.140	2	.088	-3.86693	-9.1657	1.4319
12 m	Weight loss	-4.559	2	.045	-2.00624149	-3.8996222	-.1128608
	Surface loss	-2.096	2	.171	-4.18041	-12.7639	4.4030
24 m	Weight loss	-6.366	2	.024	-2.28094298	-3.8224969	-.7393890
	Surface loss	.842	2	.488	1.15863	-4.7604	7.0776

Holm-Bonferroni correction

e)

Weight loss		
Exposure time	Raw p value	Adjusted p value
3 m pre	0.037	0.148
1 m	0.129	0.258
3 m	0.226	0.258
6 m	0.021	0.126
12 m	0.045	0.148
24 m	0.024	0.126

Surface loss		
Exposure time	Raw p value	Adjusted p value
3 m pre	0.017	0.102
1 m	0.043	0.215
3 m	0.779	0.976
6 m	0.088	0.352
12 m	0.171	0.513
24 m	0.488	0.976

6. Additional seawater exposure experiments

The first seawater redeployment of the mulch films samples was performed in September-October 2022, the sample seasonal period of the original deployment. After 4 weeks, BMF2 lost 18% in weight, similar to what was observed after 1 month of exposure in the original marine deployment (21% weight loss) and reached 30% after 5 weeks. BMF1 showed a faster degradation/fragmentation rate, losing 89% of its weight after 4 weeks while only 25% was lost in the original deployment. SEC analyses were also performed on BMF1 and BMF2 samples to better understand the degradation occurring in marine environment over 1 month of exposure (Figure S10). As observed in the original seawater experiments, a decrease in molar mass was observed over the course of 4 weeks (Figure S10). The molar mass started to decrease after 1 week of exposure for BMF2, whereas for BMF1 it already started before 1 week and the decrease rate was (about 40% of the initial molar mass after 3 weeks) than BMF2 (about 90% of the initial molar mass after 3 weeks).

The second seawater redeployment was performed in a different season, February-March 2023 (Figure S11) and had a very different outcome from the first one. There were no signs of degradation/fragmentation for either BMF1 and BMF2, with weight losses of 5 and 4% respectively after 5 weeks.

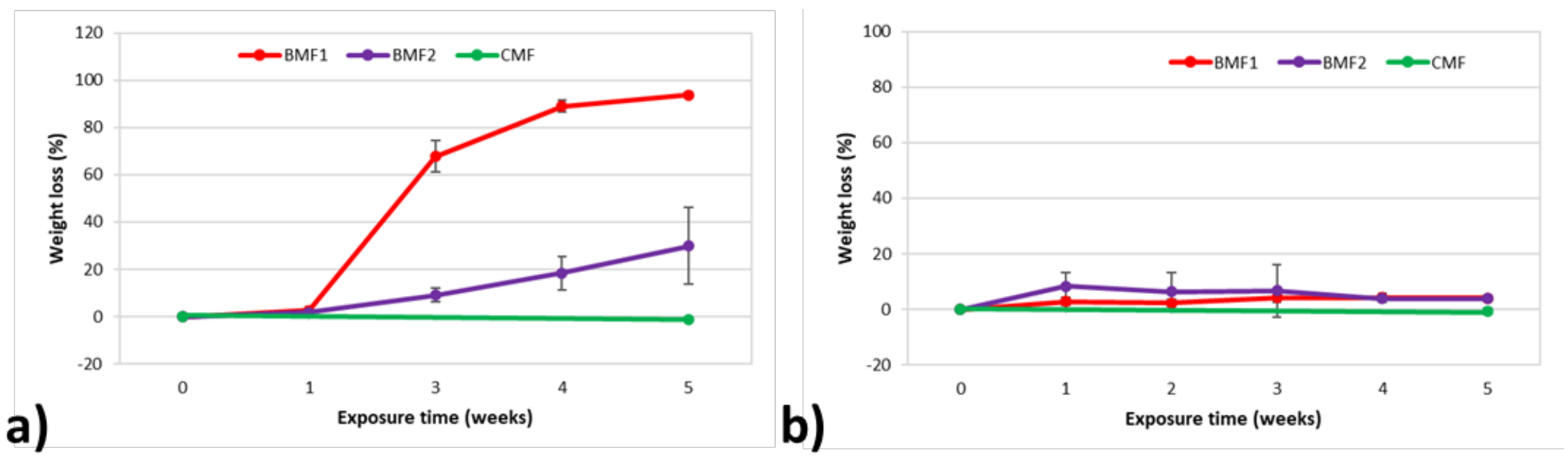

Figure S10. The average (± SD) weight loss (%) for BMF1, BMF2 and CMF samples reported in the additional seawater experiments **a)** marine1 (September-October 2022) and **b)** marine2 (February-March 2023).

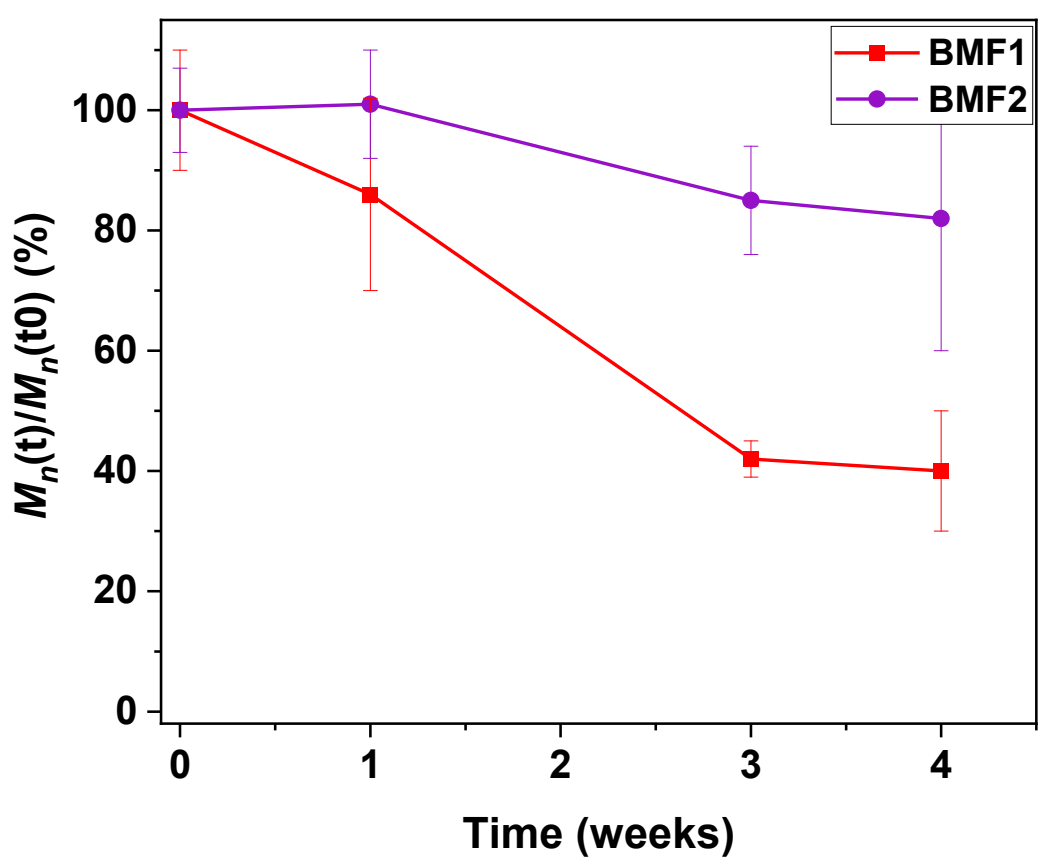

Figure S11. The evolution of molar mass relative to the average initial molar mass of the material before exposure measured by SEC in chloroform, over time for BMF1 and BMF2 marine1 samples (September-October 2022).

7. SEM analysis

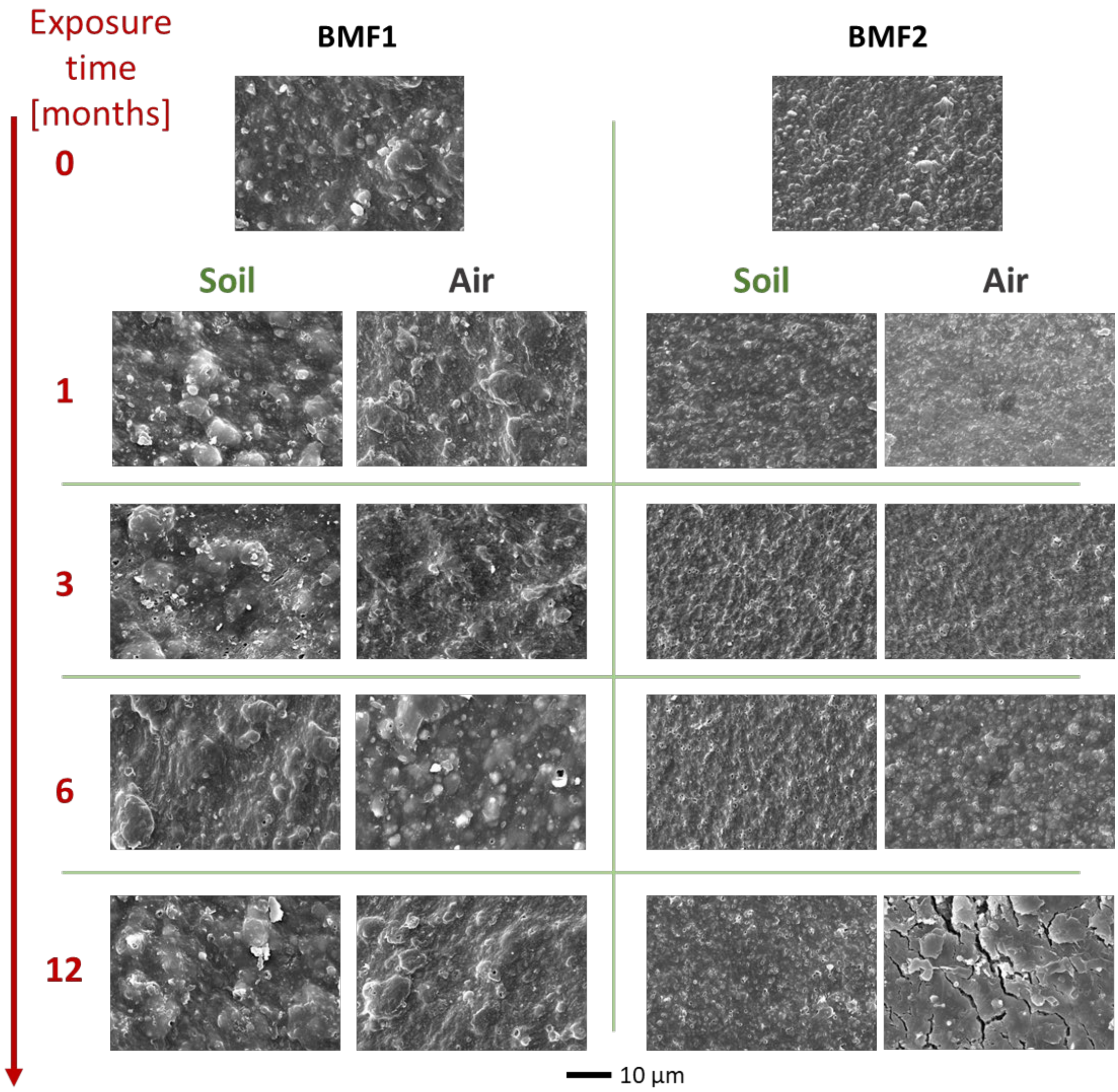

Figure S12. SEM micrographs of BMF1 and BMF2 samples unexposed (0) and exposed to soil and air for 1, 3, 6 and 12 months.

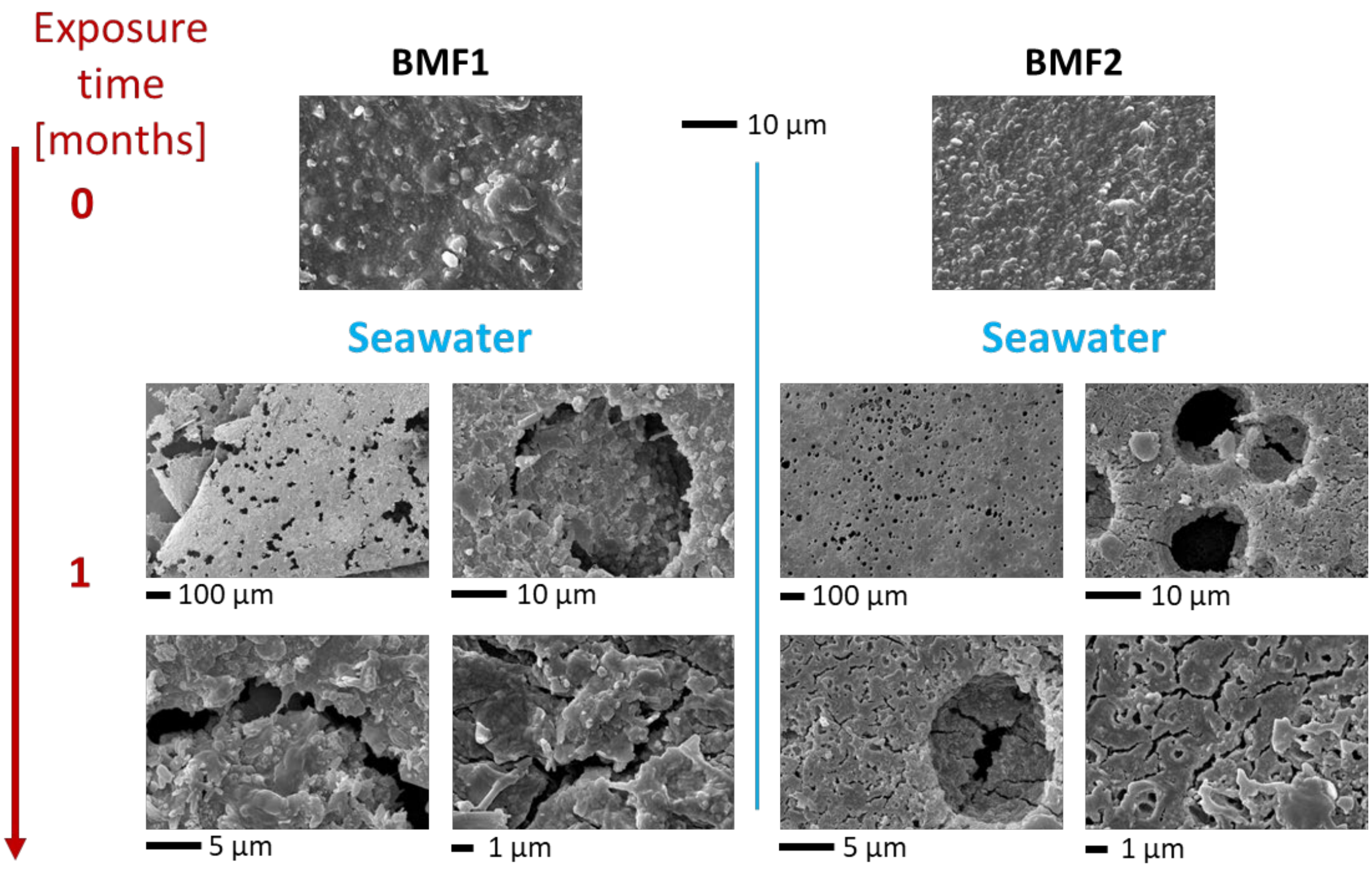

Figure S13. SEM micrographs of BMF1 and BMF2 samples unexposed (0) and seawater exposed for 1 month.

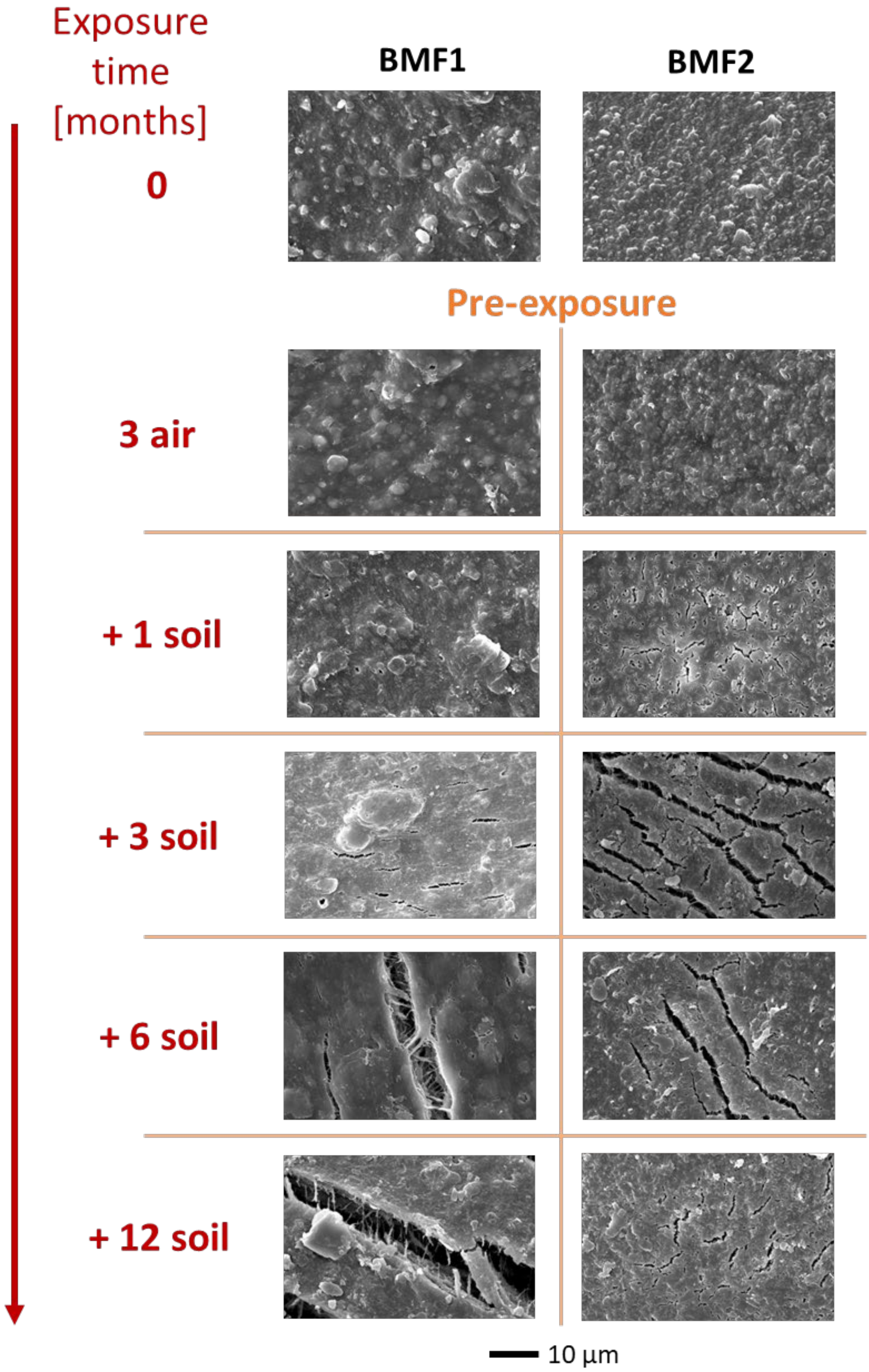

Figure S14. SEM micrographs of BMF1 and BMF2 samples unexposed, pre-exposed to air for 3 months and then buried in soil for 1, 3, 6 and 12 months.

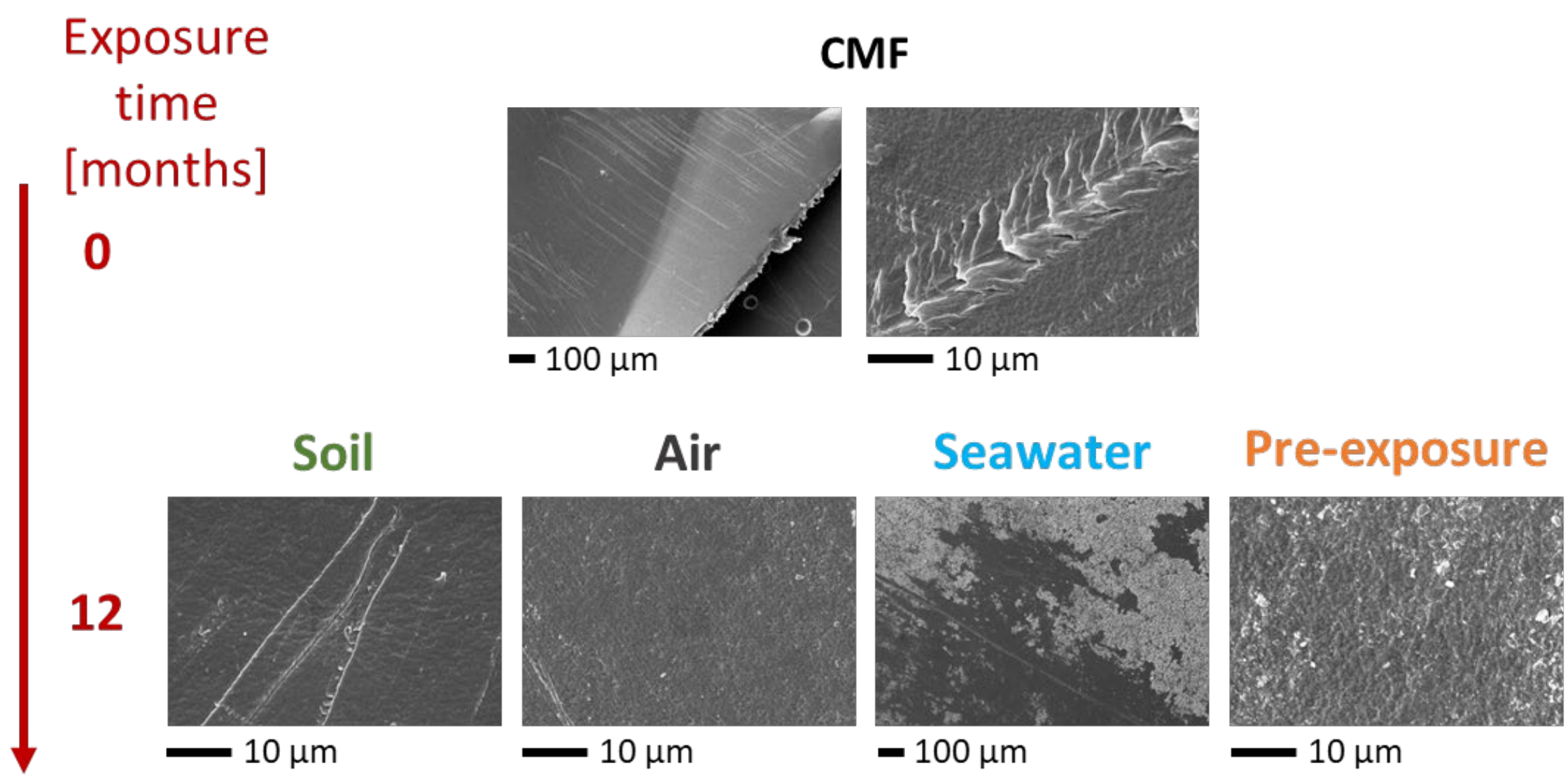

Figure S15. SEM micrographs of CMF samples unexposed and exposed for 12 months to soil, air, seawater and pre-exposure conditions.

8. SEC analysis

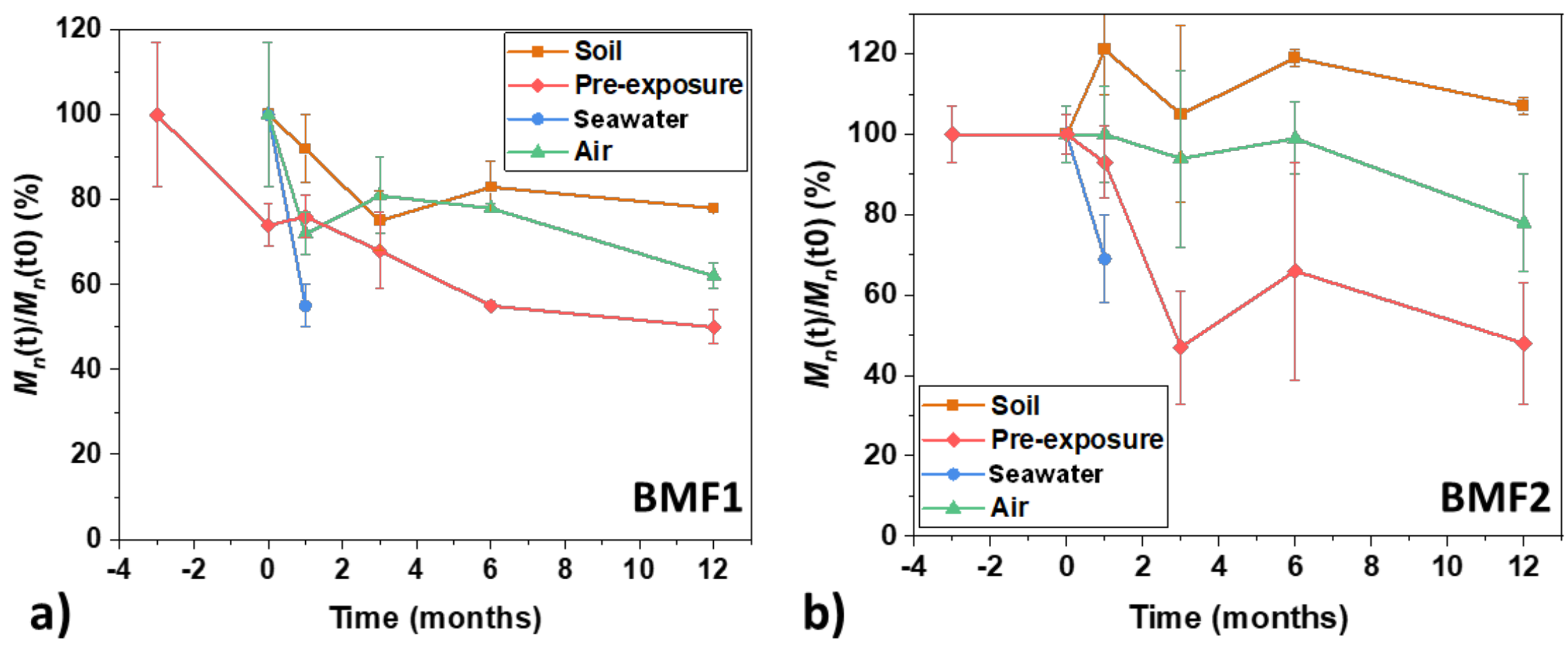

Figure S16. Evolution of molar mass relative to the average initial molar mass of the material before exposure measured by SEC in chloroform, over time for **a)** BMF1 and **b)** BMF2 soil, air, seawater and pre-exposure samples (n=3). Bars indicate standard deviation. For the pre-exposure samples, the point at-3 months corresponds to the actual 0 months (unexposed samples).

9. ATR-FTIR analysis

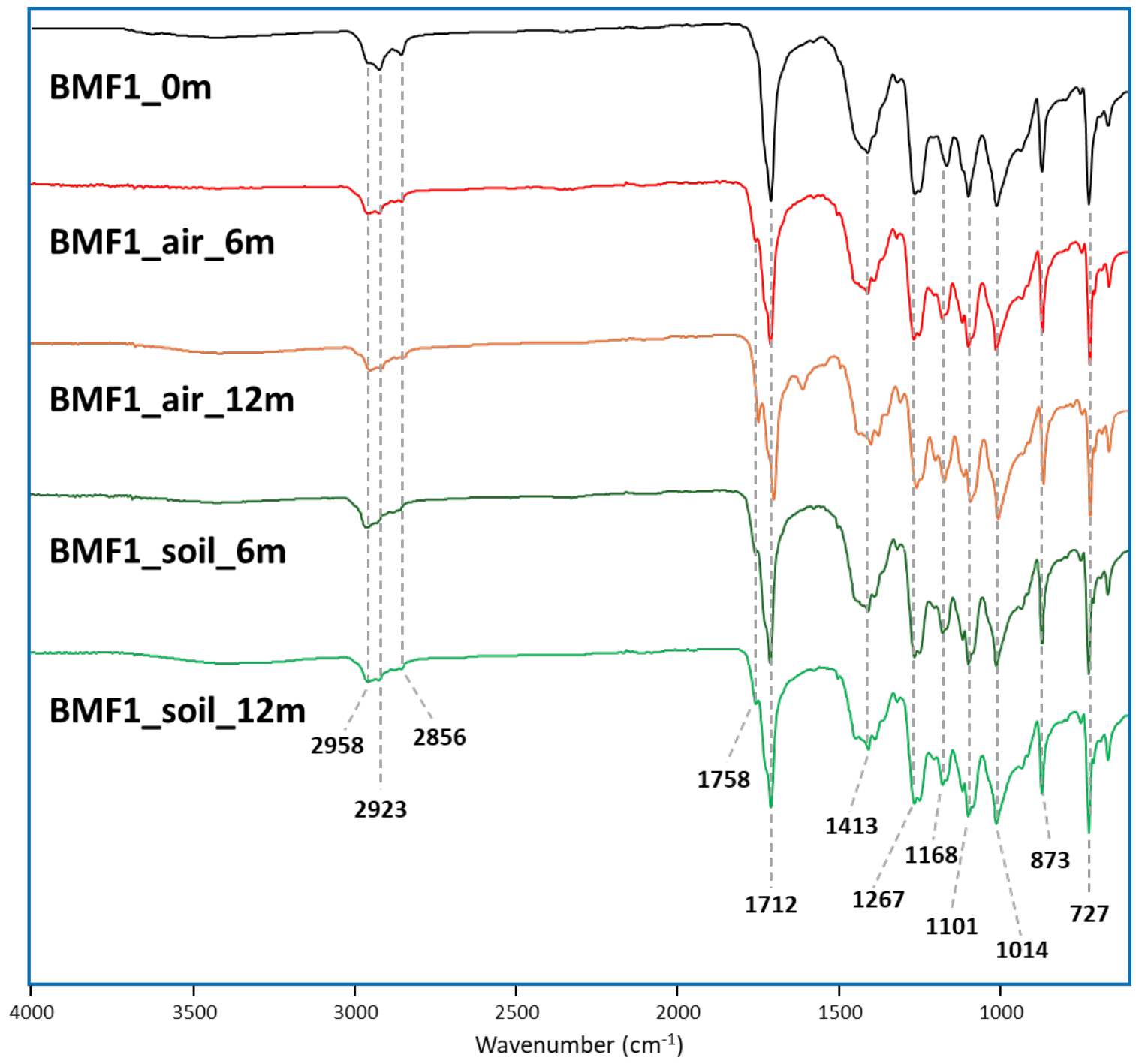

Figure S17. ATR-FTIR spectra of BMF1 samples unexposed (0m) and exposed to air or soil for 6 and 12 months.

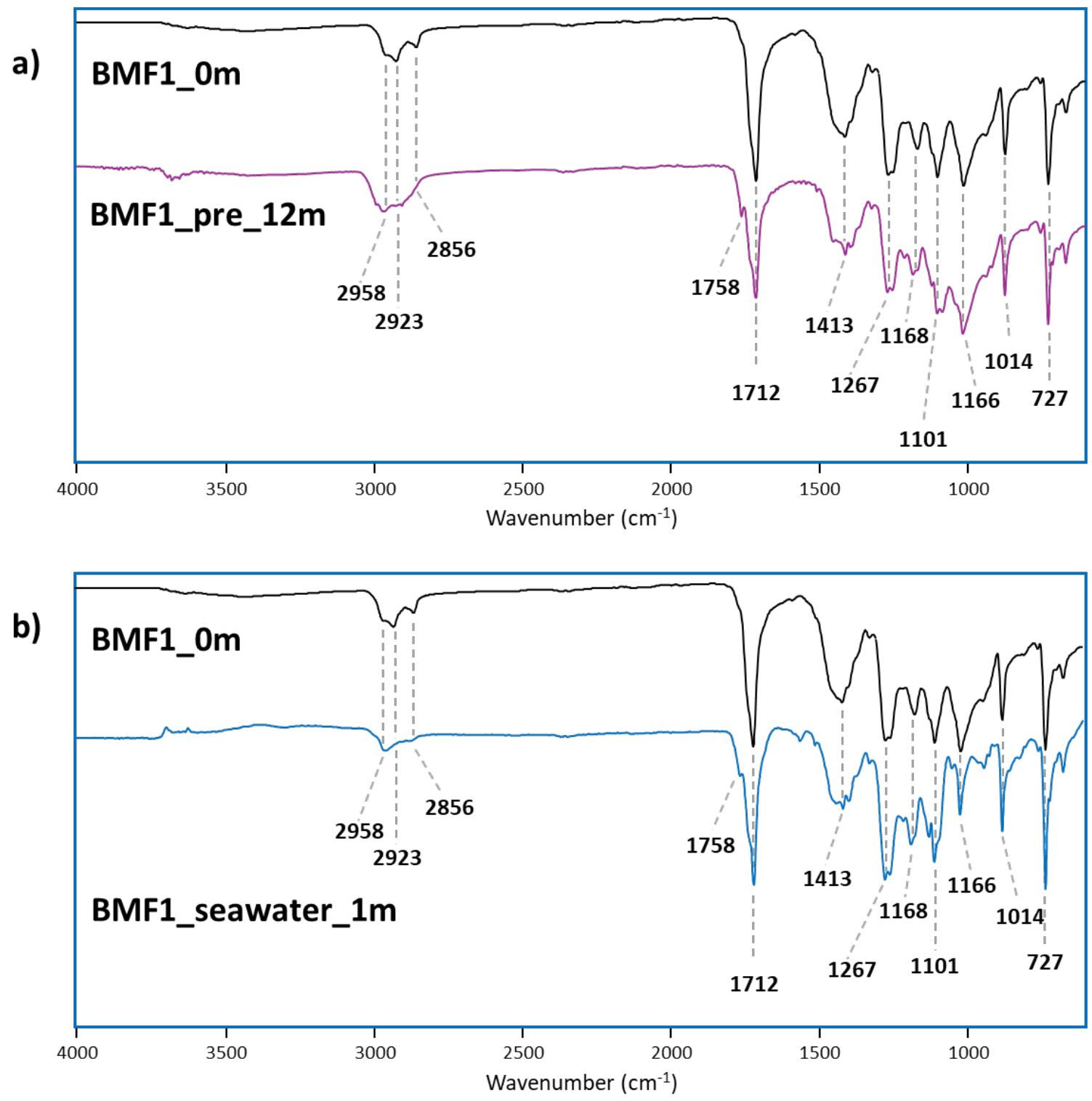

Figure S18. ATR-FTIR spectra of BMF1 samples **a)** unexposed (0m) and pre-exposed to air for 3 months and then buried in soil for 12 months; **b)** unexposed exposed to seawater for 1 month.

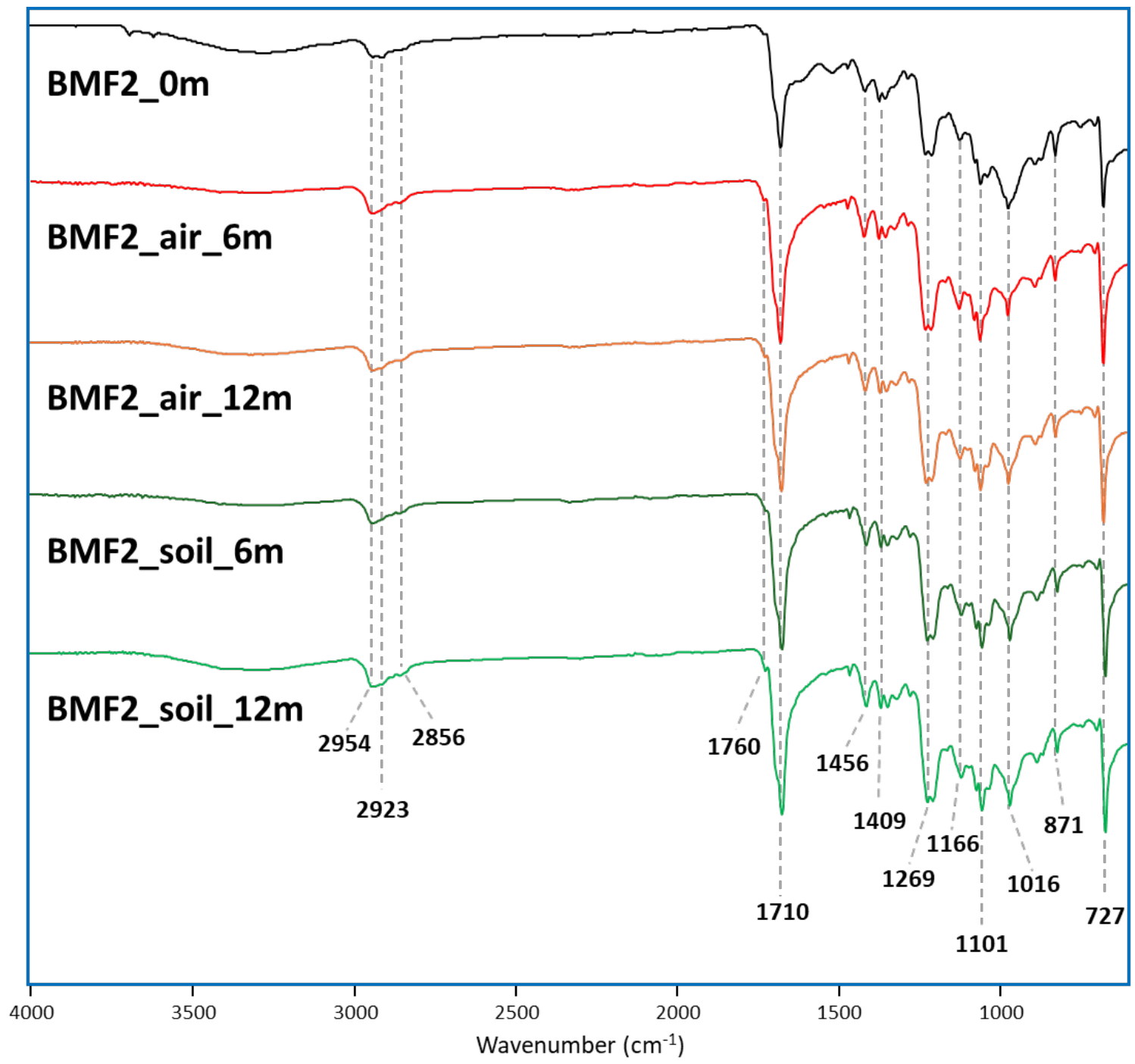

Figure S19. ATR-FTIR spectra of BMF2 samples unexposed (0m) and exposed to air or soil for 6 and 12 months.

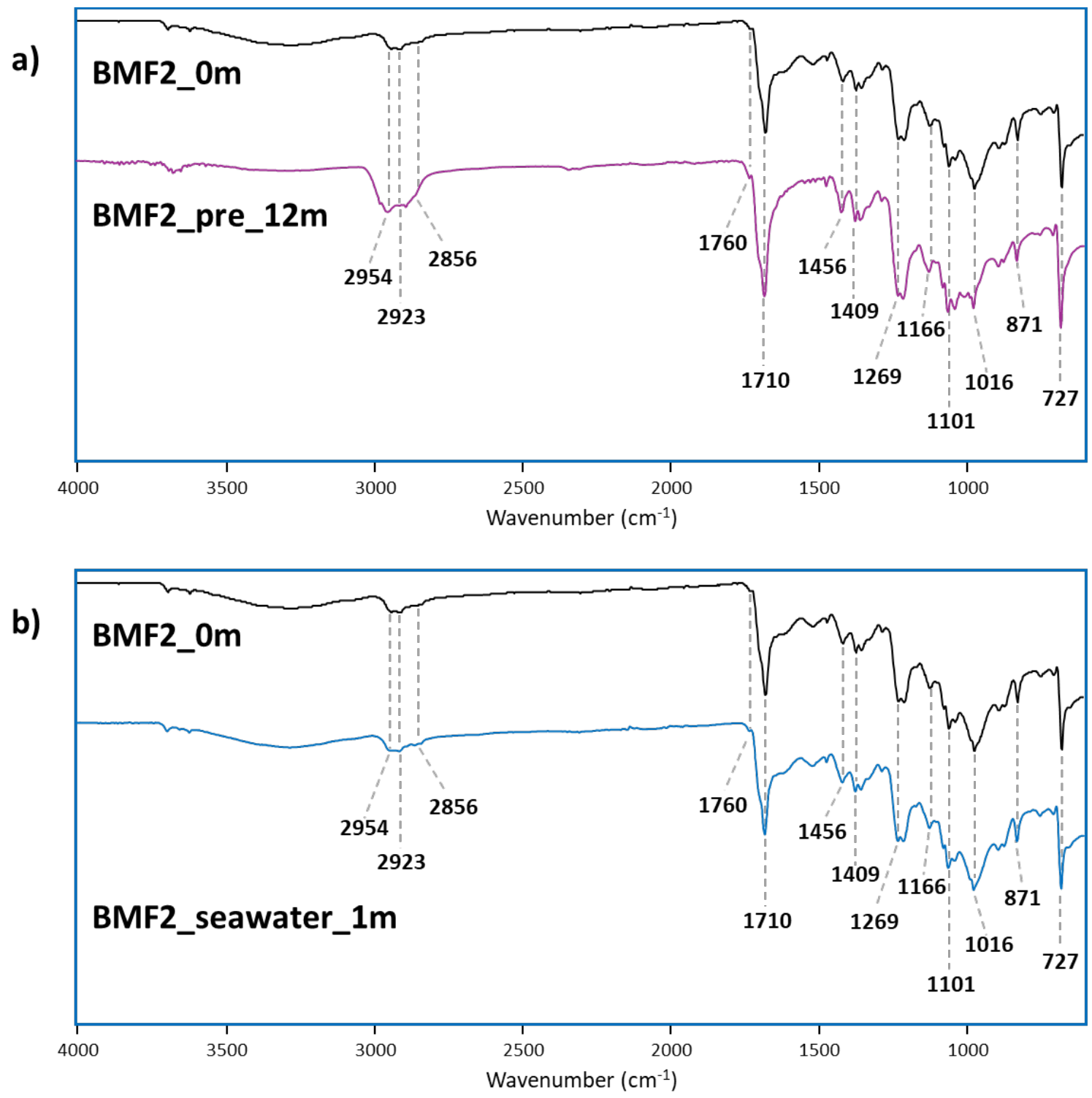

Figure S20. ATR-FTIR spectra of BMF1 samples **a)** unexposed (0m) and pre-exposed to air for 3 months and then buried in soil for 12 months; **b)** unexposed exposed to seawater for 1 month.

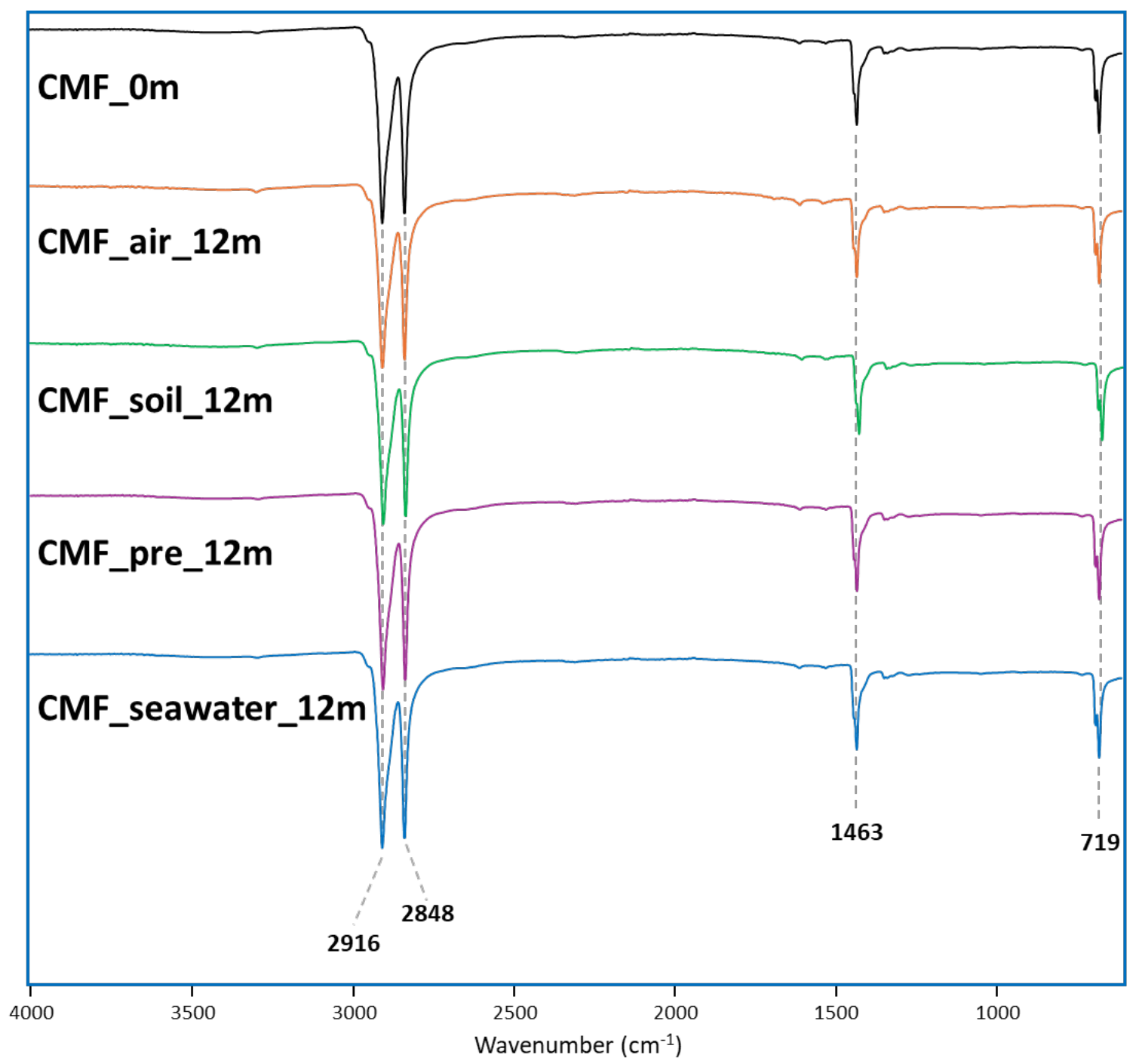

Figure S21. ATR-FTIR spectra of CMF samples unexposed (0m), exposed to air for 12 months, to soil for 12 months, pre-exposed to air for 3 months and then buried in soil for 12 months, exposed to seawater for 1 month.

Table S6. Vibrational band assignment for the ATR-FTIR spectra of BMF1, BMF2 and CMF samples shown in Figures S17-S21.

Sample	Wavenumber (cm^{-1})	Vibrational assignment
BMF1/BMF2	2954	C-H_3 asymmetrical stretching
	2923	C-H_2 asymmetrical stretching
	2856	C-H_2 symmetrical stretching
	1760	C=O stretching (ester group)
	1710	C=O stretching
	1456	C-H_2 bending
	1409	C-H_2 bending
	1269	C-O symmetric stretching
	1166	C-O stretching
	1101	C-O left-right symmetric stretching
	1016	C-H bending (aromatic ring)
	871	C-O symmetric stretching
	727	C-H bending (aromatic ring)
CMF	2916	C-H_2 asymmetrical stretching
	2848	C-H_2 symmetrical stretching
	1463	C-H_2 bending
	719	C-H_2 rocking

10. NMR analysis

Table S7. NMR results for the measurement of the PLA content in the samples.

Sample	Environment	Time point (months)	PBAT	PLA	PLA part (%)	Average (%)
BMF1	Unexposed	0	4	0.43	10%	10%
		0	4	0.46	10%	
	Soil	12	4	1.43	26%	26%
		12	4	1.43	26%	
	Pre-exposure	3+12	4	1.53	28%	26%
		3+12	4	1.35	25%	
	Air	12	4	1.46	27%	28%
		12	4	1.61	29%	
BMF2	Unexposed	0	4	0.33	8%	8%
		0	4	0.36	8%	
	Soil	12	4	0.52	12%	12%
		12	4	0.59	13%	
	Pre-exposure*	3+12	4	0.39	9%	9%
	Air	12	4	0.36	8%	8%
		12	4	0.33	8%	

*only one replicate was analysed for this sample due to technical reasons.

The PLA part was calculated as follows:

$$\%_{PLA} = \frac{A_{PLA}}{A_{PBAT} + A_{PLA}}$$

The singlet corresponding to 4 H at 8.1 ppm was used for a unit of PBAT and the quadruplet at 4.2 ppm was used for a unit of PLA.

11. Py-GC-MS analysis

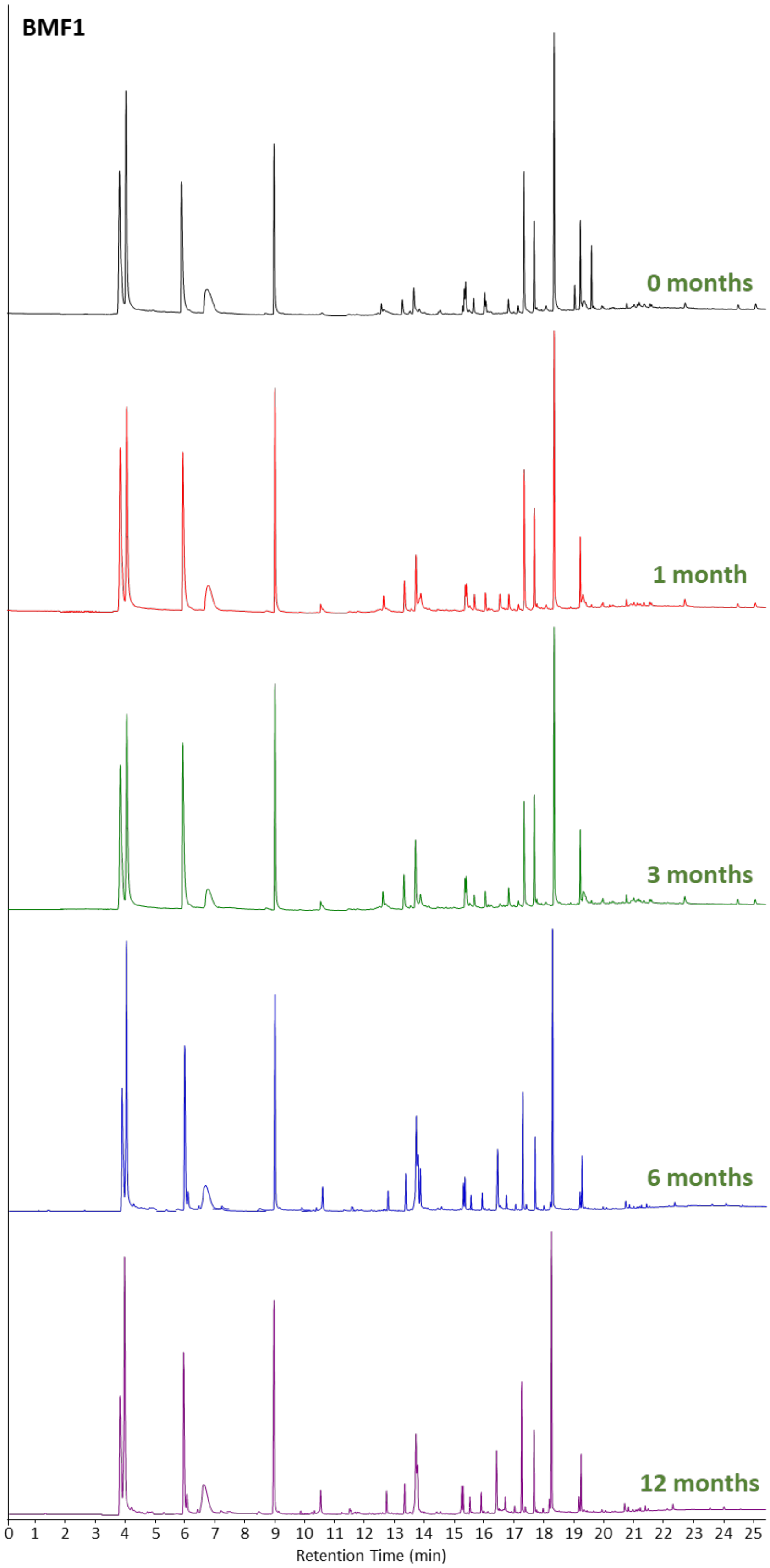

Figure S22. Py-GC-MS chromatograms at 600 °C of BMF1 samples unexposed (0 months) and exposed to soil for 1, 3, 6 and 12 months.

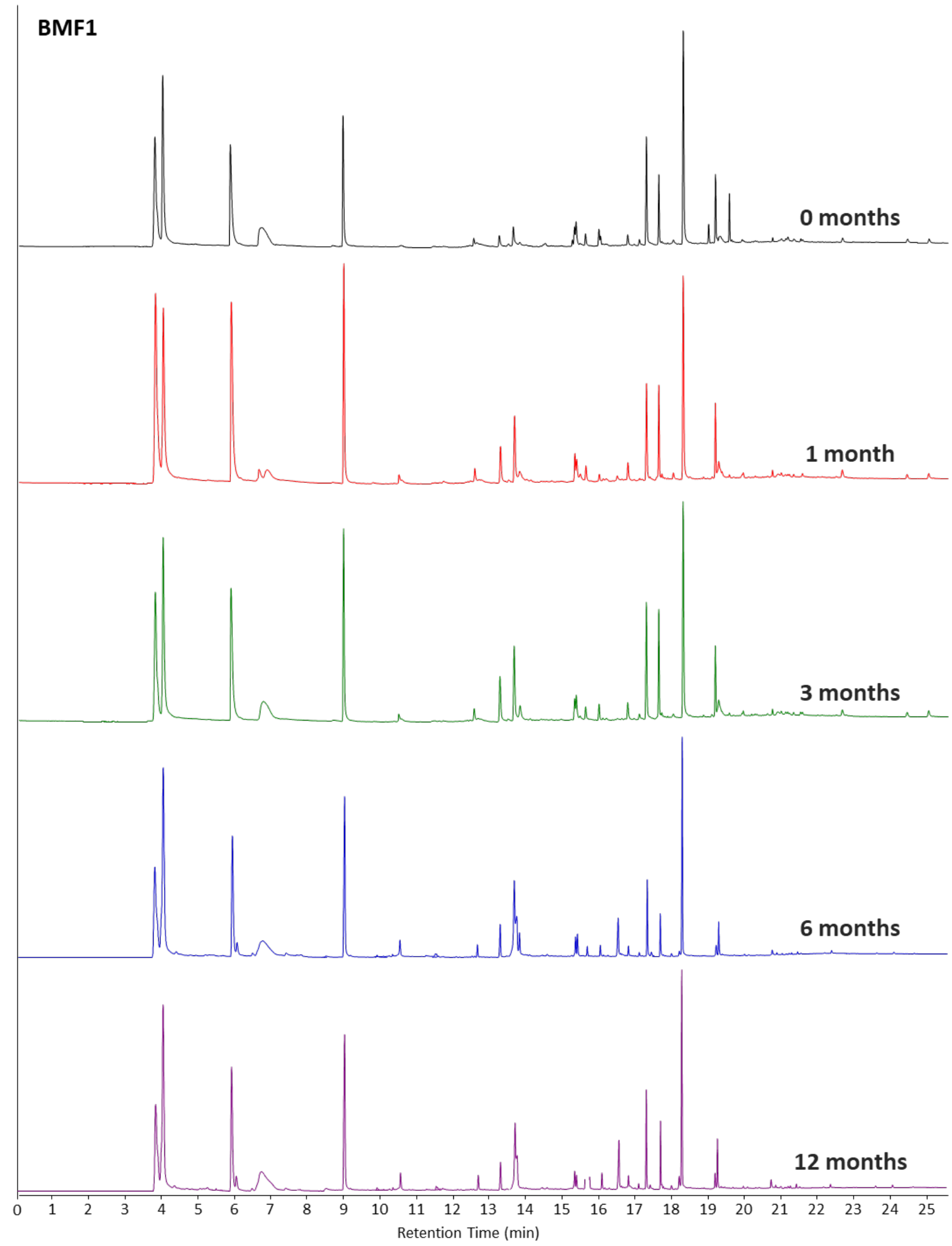

Figure S23. Py-GC-MS chromatograms at 600 °C of BMF1 samples unexposed (0 months) and exposed to air for 1, 3, 6 and 12 months.

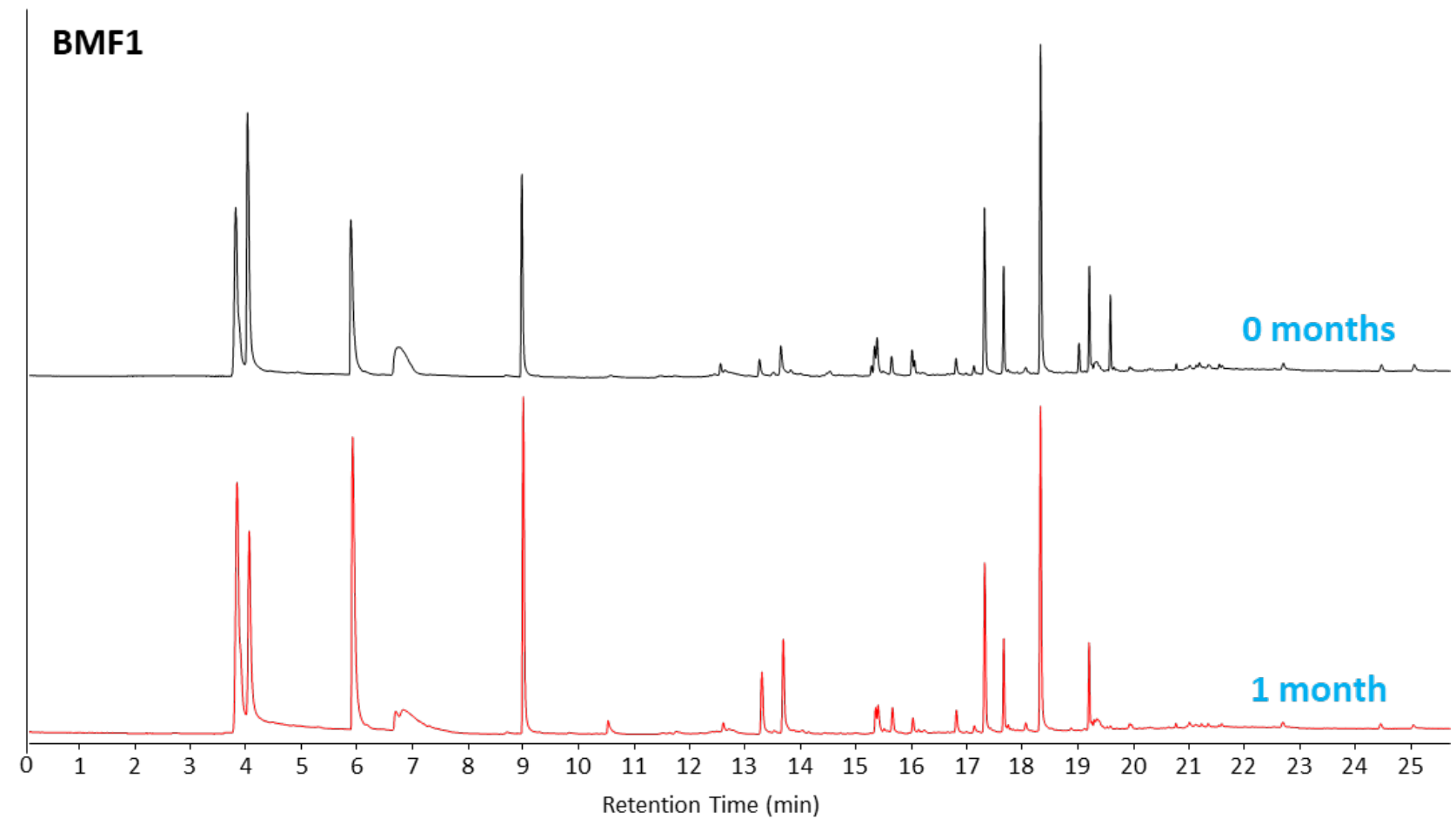

Figure S24. Py-GC-MS chromatograms at 600 °C of BMF1 samples unexposed (0 months) and exposed to seawater for 1 month.

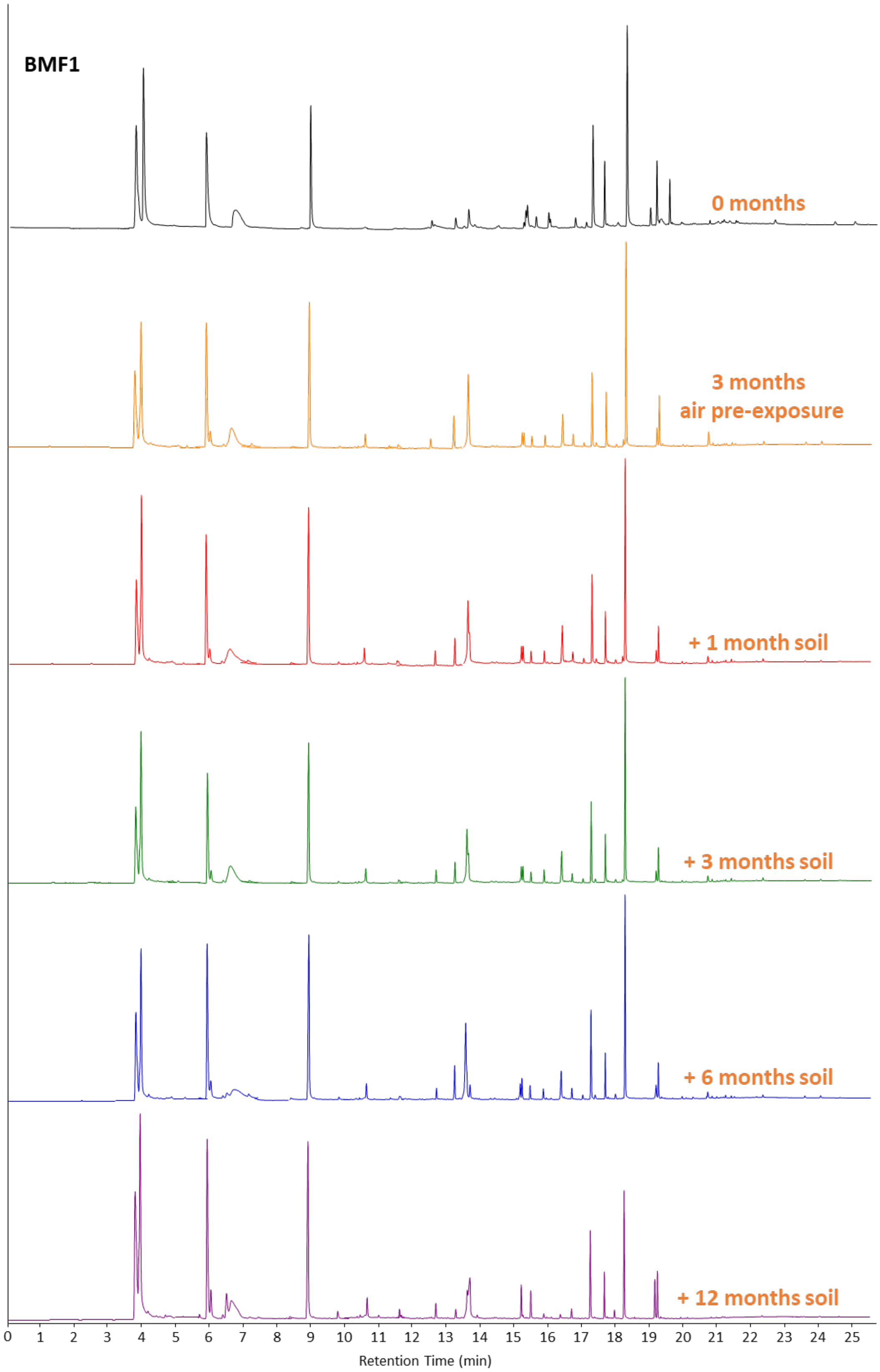

Figure S25. Py-GC-MS chromatograms at 600 °C of BMF1 samples unexposed (0 months), pre-exposed to air for 3 months and then buried in soil for 1, 3, 6 and 12 months.

Table S8. Main pyrolysis products detected in the Py-GC-MS chromatograms of BMF1 unexposed samples. The base peak in the mass spectrum of each compound is highlighted in bold.

t (min)	Compound	Main ions (m/z)
3.8	Carbon dioxide	**44**
4.0	1,3-butadiene	39, 50, 51, 53, **54**
5.9	Tetrahydrofuran	39, 41, **42**, 71, 72
9.0	Cyclopentanone	39, 41, 42, **55**, 56, 84
12.6	Pentanoic acid, 3-butenyl ester	39, 41, 54, 55, 57, **85**, 115
13.3	3,6-dimethyl-1,4-dioxane-2,5-dione (*meso* form)	43, 45, **56**, 144
13.6	3,6-dimethyl-1,4-dioxane-2,5-dione (*DL* form)	43, 45, **56**, 144
15.3	Benzoic acid, 3-butenyl ester	41, 54, 77, **105**, 122, 176
15.4	1,6-diisocyanato-hexane	41, 43, **56**, 69, 85, 99, 126
15.6	Biphenyl	51, 63, 76, 89, 115, **154**
16.0	Diphenylmethane	51, 65, 83, 91, 115, 152, **167**
16.8	1,6-dioxacyclododecane-7,12-dione	41, 55, 71, 84, 100, 129, **172**
17.1	Fluorene	63, 70, 83, 98, 139, 165, **166**
17.3	Benzophenone	51, 77, **105**, 152, 182
17.7	Adipic acid, di(3-butenyl) ester	39, **55**, 83, 101, 111, 129
18.3	Anthracene-d10	**188**
19.0	1,6-dioxacyclohexadecane-7,16-dione	41, **55**, 71, 84, 98, 114, 166, 185
19.2	Terephthalic acid, di(but-3-enyl) ester	54, 76, 104, 121, 149, **203**, 274
19.2	Triphenylmethane	51, 77, 115, 152, **165**, 167, 244
19.6	Sebacic acid, di(but-3-enyl) ester	41, **55**, 97, 139, 185, 239, 267

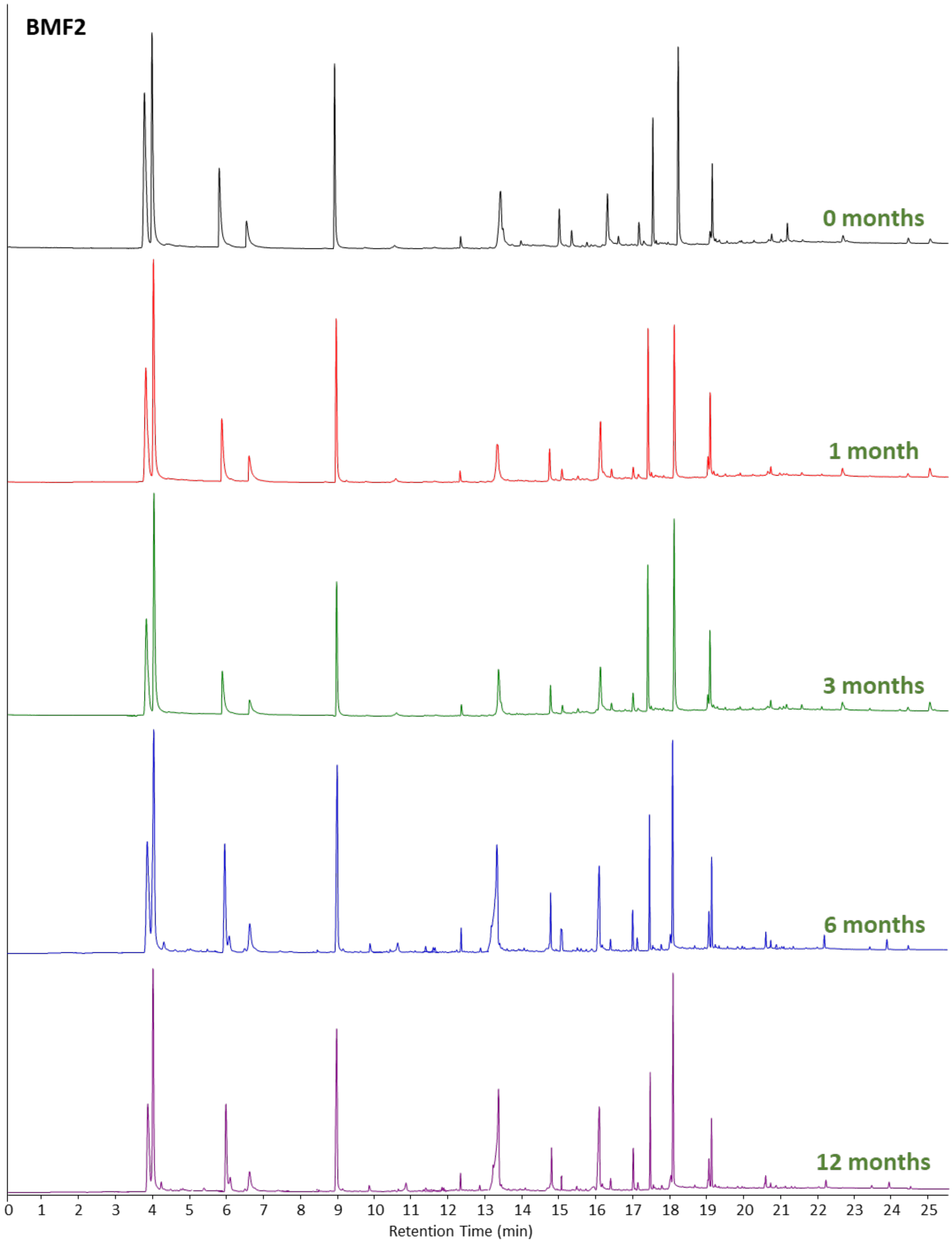

Figure S26. Py-GC-MS chromatograms at 600 °C of BMF2 samples unexposed (0 months) and exposed to soil for 1, 3, 6 and 12 months.

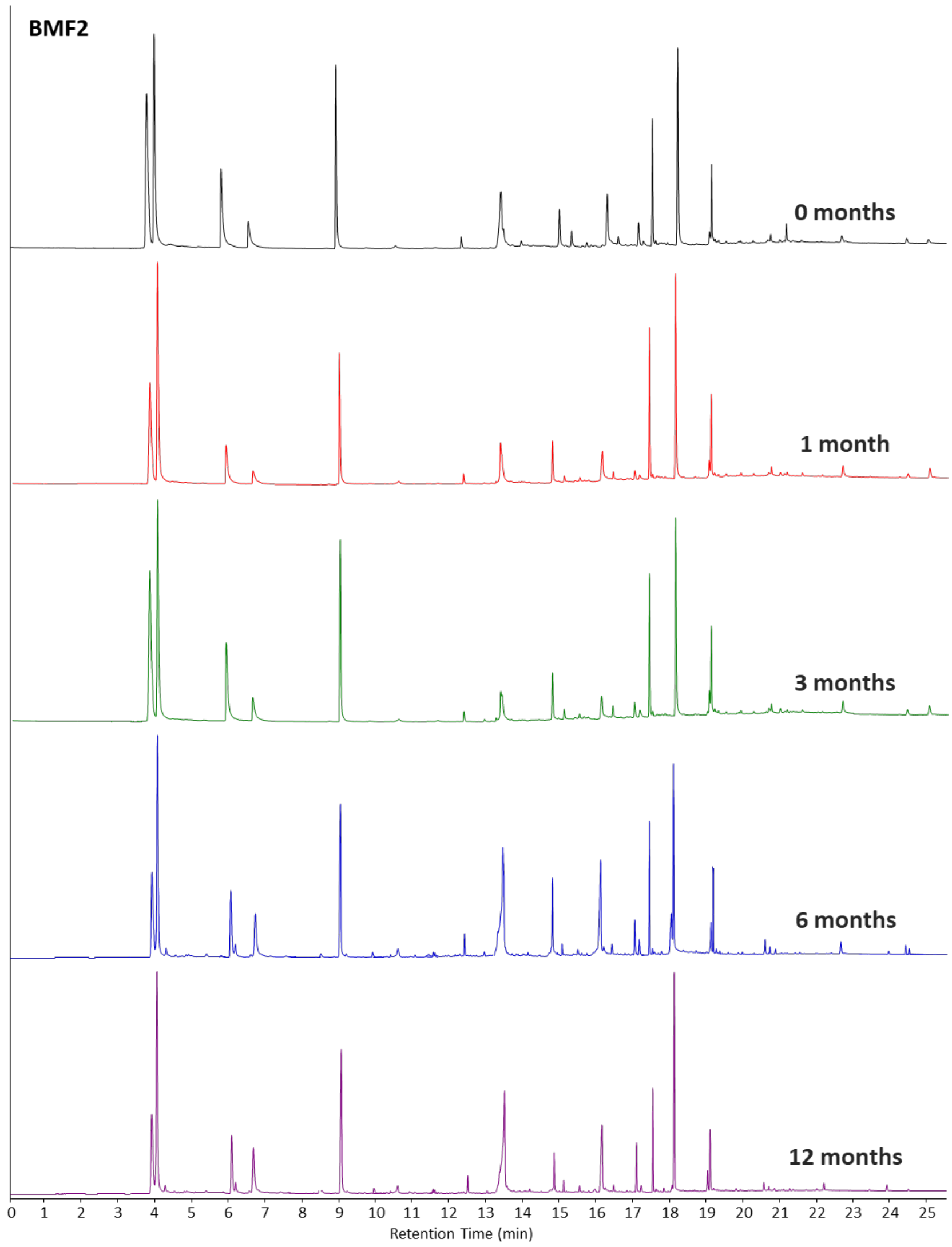

Figure S27. Py-GC-MS chromatograms at 600 °C of BMF2 samples unexposed (0 months) and exposed to air for 1, 3, 6 and 12 months.

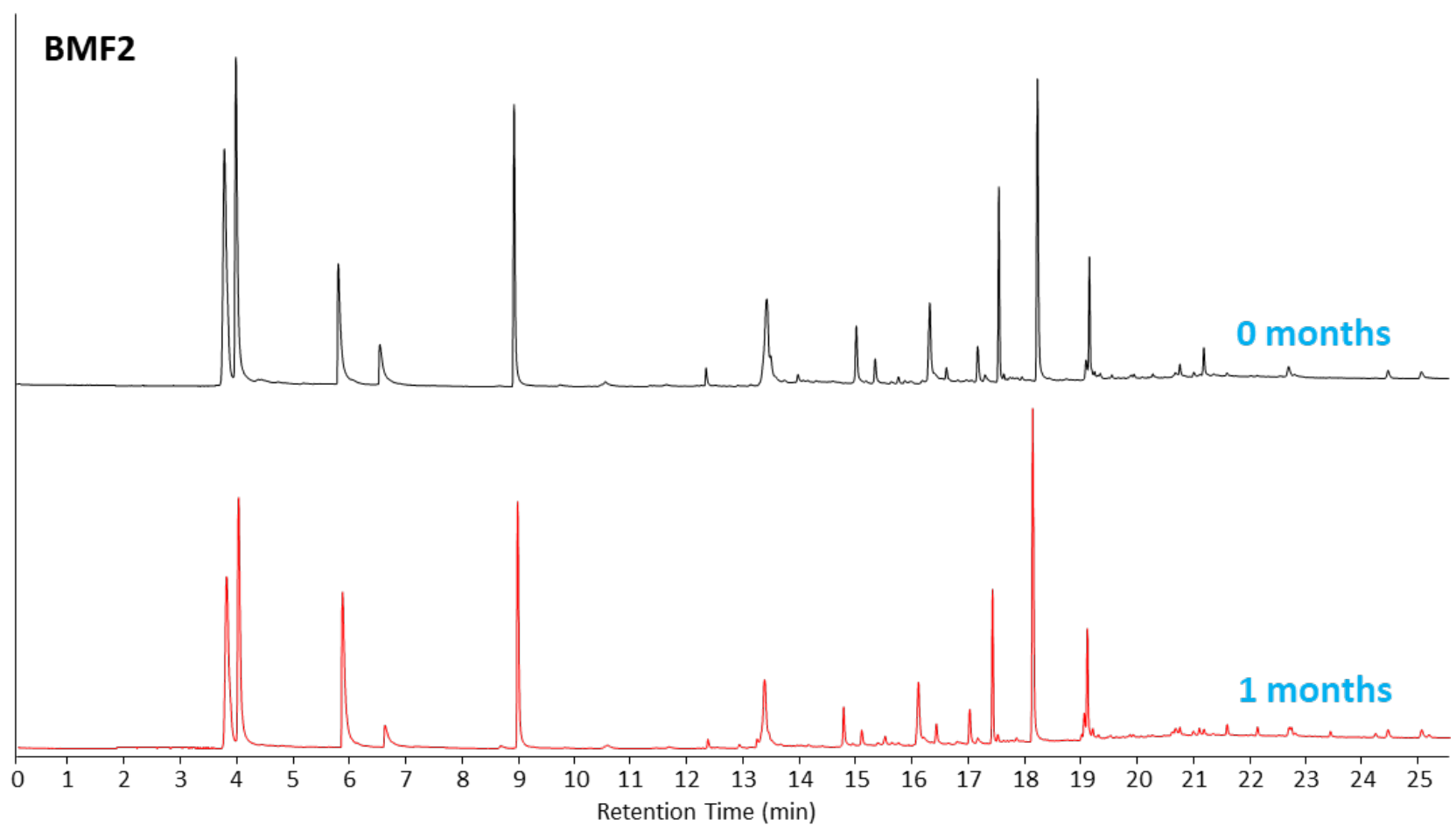

Figure S28. Py-GC-MS chromatograms at 600 °C of BMF2 samples unexposed (0 months) and exposed to seawater for 1 month.

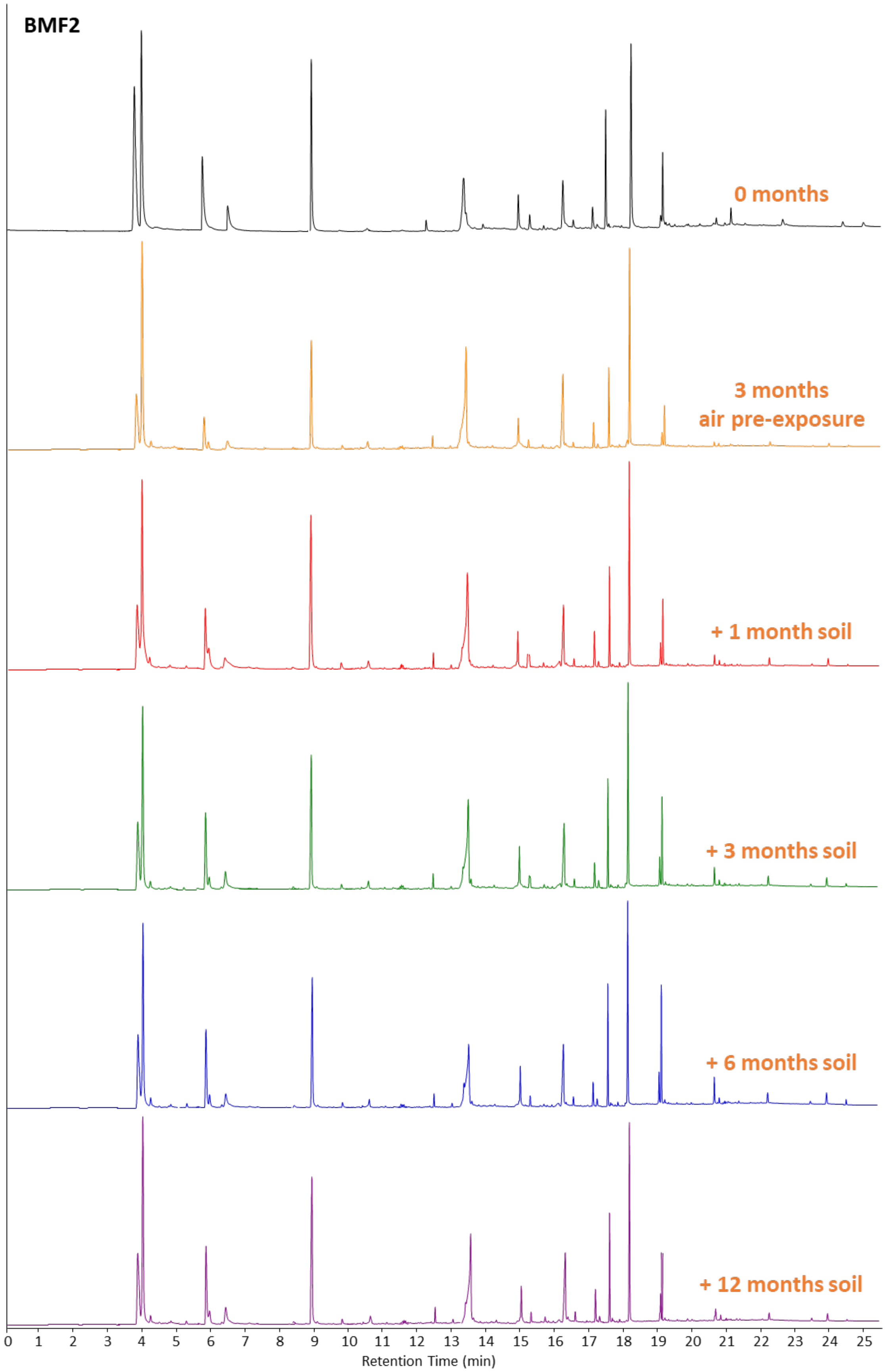

Figure S29. Py-GC-MS chromatograms at 600 °C of BMF2 samples unexposed (0 months), pre-exposed to air for 3 months and then buried in soil for 1, 3, 6 and 12 months.

Table S9. Main pyrolysis products detected in the Py-GC-MS chromatograms of BMF2 unexposed samples. The base peak in the mass spectrum of each compound is highlighted in bold.

t (min)	Compound	Main ions (m/z)
3.8	Carbon dioxide	**44**
4.0	1,3-butadiene	39, 50, 51, 53, **54**
5.8	Tetrahydrofuran	39, 41, **42**, 71, 72
8.9	Cyclopentanone	39, 41, 42, **55**, 56, 84
12.4	Pentanoic acid, 3-butenyl ester	39, 41, 54, 55, 57, **85**, 115
13.5	Benzoic acid	39, 51, 77, **105**, 122
15.1	Benzoic acid, 3-butenyl ester	41, 54, 77, **105**, 122, 176
15.4	Biphenyl	51, 63, 76, 89, 115, **154**
15.8	Diphenylmethane	51, 65, 83, 91, 115, 152, **167**
16.3	Adipic acid, 3-butenyl ester	54, 55, 83, 101, 111, **129**
16.6	1,6-dioxacyclododecane-7,12-dione	41, 55, 71, 84, 100, 129, **172**
17.2	Benzophenone	51, 77, **105**, 152, 182
17.6	Adipic acid, di(3-butenyl) ester	39, **55**, 83, 101, 111, 129
18.3	Anthracene-d10	**188**
19.2	Terephthalic acid, di(but-3-enyl) ester	54, 76, 104, 121, 149, **203**, 274

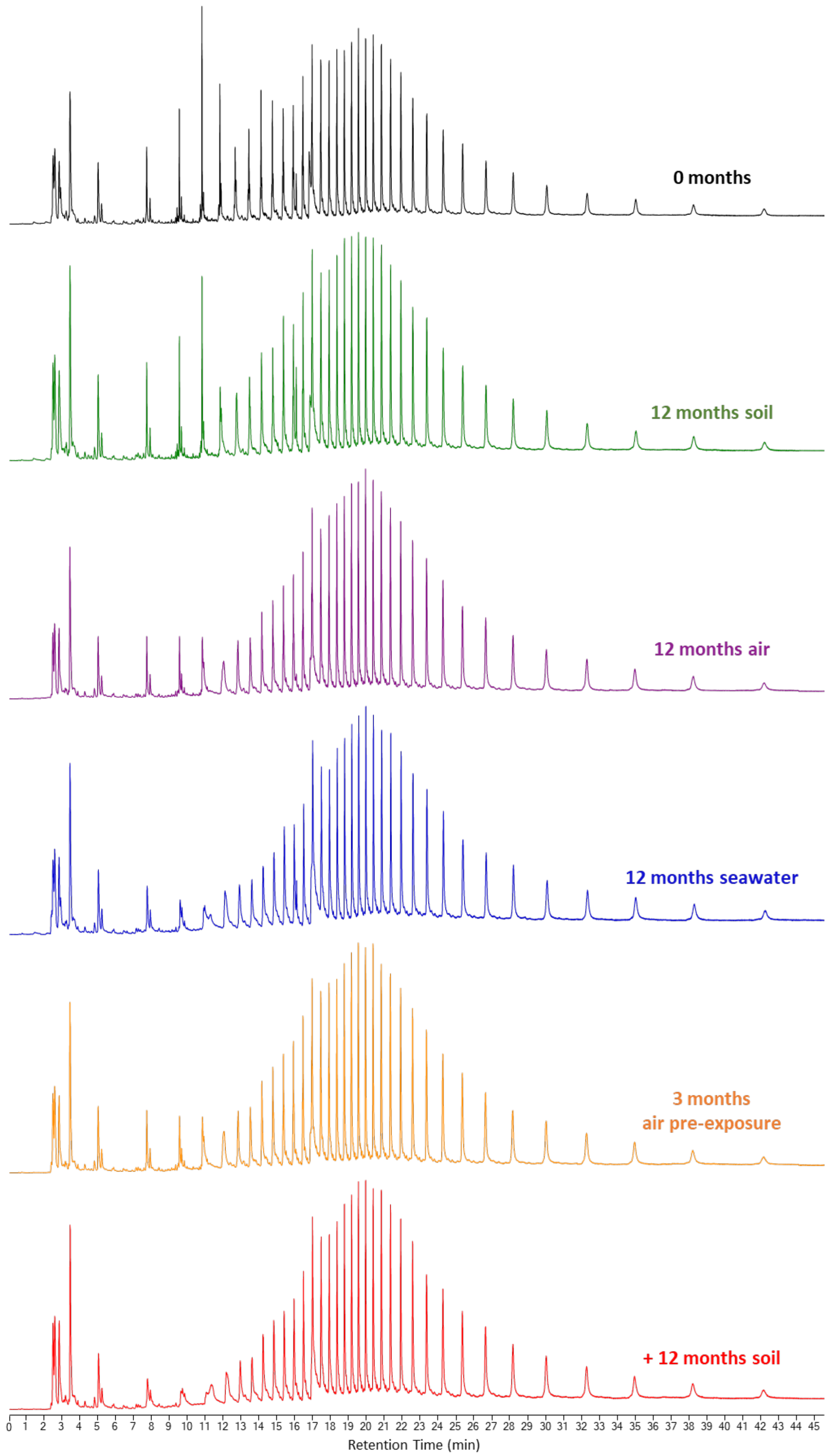

Figure S30. Py-GC-MS chromatograms at 600 °C of CMF samples unexposed (0 months), exposed to soil for 12 months, to air for 12 months, to seawater conditions for 12 months and pre-exposed to air for 3 months and then buried in soil for 12 months.

Table S10. Main pyrolysis products detected in the Py-GC-MS chromatograms of CMF unexposed samples. The base peak in the mass spectrum of each compound is highlighted in bold.

t (min)	Compound	Main ions (m/z)
2.4	Carbon dioxide	**44**
2.5	Propylene	39, 40, **41**, 42
2.6	2-butene	39, **41**, 50, 55, 56
2.9	1-pentene	39, 41, **42**, 55, 70
3.5	1-hexene	41, **56**, 69, 84
3.6	n-hexane	41, 43, **57**, 86
5.0	1-heptene	41, **56**, 70, 83, 98
5.2	n-heptane	**43**, 57, 71, 85, 100
7.7	1-octene	43, **55**, 70, 83, 97, 112
7.9	n-octane	**43**, 57, 71, 85, 114
9.6	1-nonene	43, **56**, 69, 83, 97, 126
9.7	Nonane	**43**, 57, 71, 85, 99, 128
10.8	1,9-decadiene	41, **55**, 67, 81, 95, 110
10.8	1-decene	41, **56**, 70, 83, 97, 111, 140
10.9	n-Decane	43, **57**, 71, 85, 99,113, 142
11.8	1,10-undecadiene	41, **55**, 67, 82, 95, 109, 124
11.8	1-undecene	43, 55, 70, 83, 97, 111, 126, 154
11.9	Undecane	43, **57**, 71, 85, 98, 113, 127, 156
12.6	1,11-dodecadiene	41, **55**, 67, 81, 96, 109, 124, 138
12.7	1-dodecene	43, **55**, 69, 83, 97, 111, 125, 140, 168
12.7	Dodecane	43, **57**, 71, 85, 98, 112, 127, 170
13.4	1,12-tridecadiene	41, **55**, 67, 81, 95, 109, 152, 180
13.5	1-tridecene	41, **55**, 69, 83, 97, 111, 125, 139, 154, 182
13.5	Tridecane	43, **57**, 71, 85, 97, 112, 127, 184
14.1	1,13-tetradecadiene	41, **55**, 67,81, 96, 109, 166
14.1	1-tetradecene	41, **55**, 69, 93, 97, 111, 125, 140, 168, 196
14.2	Tetradecane	43, **57**, 71, 85, 99, 113, 127, 141, 198
14.7	1,14-pentadecadiene	41, **55**, 69, 82, 96, 109, 123
14.8	1-pentadecene	41, **55**, 69, 83, 97, 111, 125, 210
14.8	Pentadecane	43, **57**, 71, 85, 99, 113, 212
15.3	1,15-hexadecadiene	41, **55**, 69, 82, 96, 109, 123
15.4	1-hexadecene	41, **55**, 69, 83, 97, 111, 125, 224
15.4	Hexadecane	43, **57**, 71, 85, 99, 226
15.9	1,16-heptadecadiene	41, 55, 69, 82, 96, 109, 123, 137
15.9	1-heptadecene	43, **57**, 69, 83, 97, 111, 125, 140, 238
15.9	Heptadecane	43, **57**, 71, 85, 99, 113, 240
16.9	1,17-octadecadiene	41, **55**, 69, 82, 96, 109, 123
17.0	1-octadecene	43, **57**, 69, 83, 97, 111, 125, 139, 252
17.0	Octadecane	43, 57, 71, 85, 97, 113, 127, 254
17.4	1,18-nonadecadiene	41, **55**, 69, 82, 96, 109, 123, 137
17.4	1-nonadecene	43, 55, 69, **83**, 97, 111, 125, 139
17.5	Nonadecane	43, **57**, 71, 85, 97, 111, 127, 268

17.9	1,19-eicosadiene	41, **55**, 69, 82, 96, 109, 123, 137
17.9	1-eicosene	43, **57**, 71, 83, 97, 111, 125, 139, 280
17.9	Eicosane	43, **57**, 71, 85, 97, 113, 127, 141, 282
18.3	Anthracene-d10	**188**
18.3	1,20-heneicosadiene	41, **55**, 69, 82, 96, 109, 123, 137
18.3	1-heneicosene	43, **57**, 69, 83, 97, 111, 125, 139, 294
18.4	Heneicosane	43, **57**, 71, 85, 97, 113, 296
18.8	1,21-docosadiene	41, **55**, 69, 82, 96, 109, 123, 137, 151
18.8	1-docosene	43, **55**, 69, 83, 97, 111, 125, 139, 308
18.8	Docosane	43, **57**, 71, 85, 99, 113, 127, 141, 310
19.1	1-tricosene	43, **57**, 69, 83, 97, 111, 125, 322
19.6	1-tetracosene	43, **57**, 69, 83, 97, 111, 125, 139, 336
20.0	1-pentacosene	43, **57**, 69, 83, 97, 111, 125, 139, 350
20.4	1-hexacosene	43, **57**, 69, 83, 97, 111, 125, 139, 153, 364
20.9	1-heptacosene	43, **57**, 69, 83, 97, 111, 125, 139, 153, 378
21.4	1-octacosene	43, **57**, 69, 83, 97, 111, 125, 139, 153, 390
21.9	1-nonacosene	43, **57**, 69, 83, 97, 111, 125, 139, 153, 406
22.6	1-triacontene	43, 57, 69, 83, 97, 111, 125, 139, 153, 167, 418
23.4	1-hentriacontene	43, **57**, 69, 83, 97, 111, 125, 139, 153, 167, 432
24.3	1-dotriacontene	43, 57, 69, 83, 97, 111, 125, 139, 153, 167, 446
25.4	1-tritriacontene	43, **57**, 69, 83, 97, 111, 125, 139, 153, 167
26.7	1-tetratriacontene	43, **57**, 71, 83, 97, 111, 125, 139, 153, 167, 181, 474
28.2	17-pentatriacontene	43, **57**, 69, 83, 97, 111, 125, 139, 153, 167, 181, 490
30.1	1-hexatriacontene	43, **57**, 71, 83, 97, 111, 125, 139, 153, 167, 181, 502
32.3	1-heptatriacontene	43, **57**, 71, 83, 97, 111, 125, 139, 153, 167, 181, 195, 516
34.9	1-octatriacontene	43, **57**, 71, 83, 97, 111, 125, 139, 153, 167, 181, 195, 530
38.3	1-nonatriacontene	43, **57**, 71, 83, 97, 111, 125, 139, 153, 167, 181, 195, 209, 544
42.2	1-tetracontene	43, **57**, 71, 83, 97, 111, 125, 139, 153, 167, 181, 195, 209

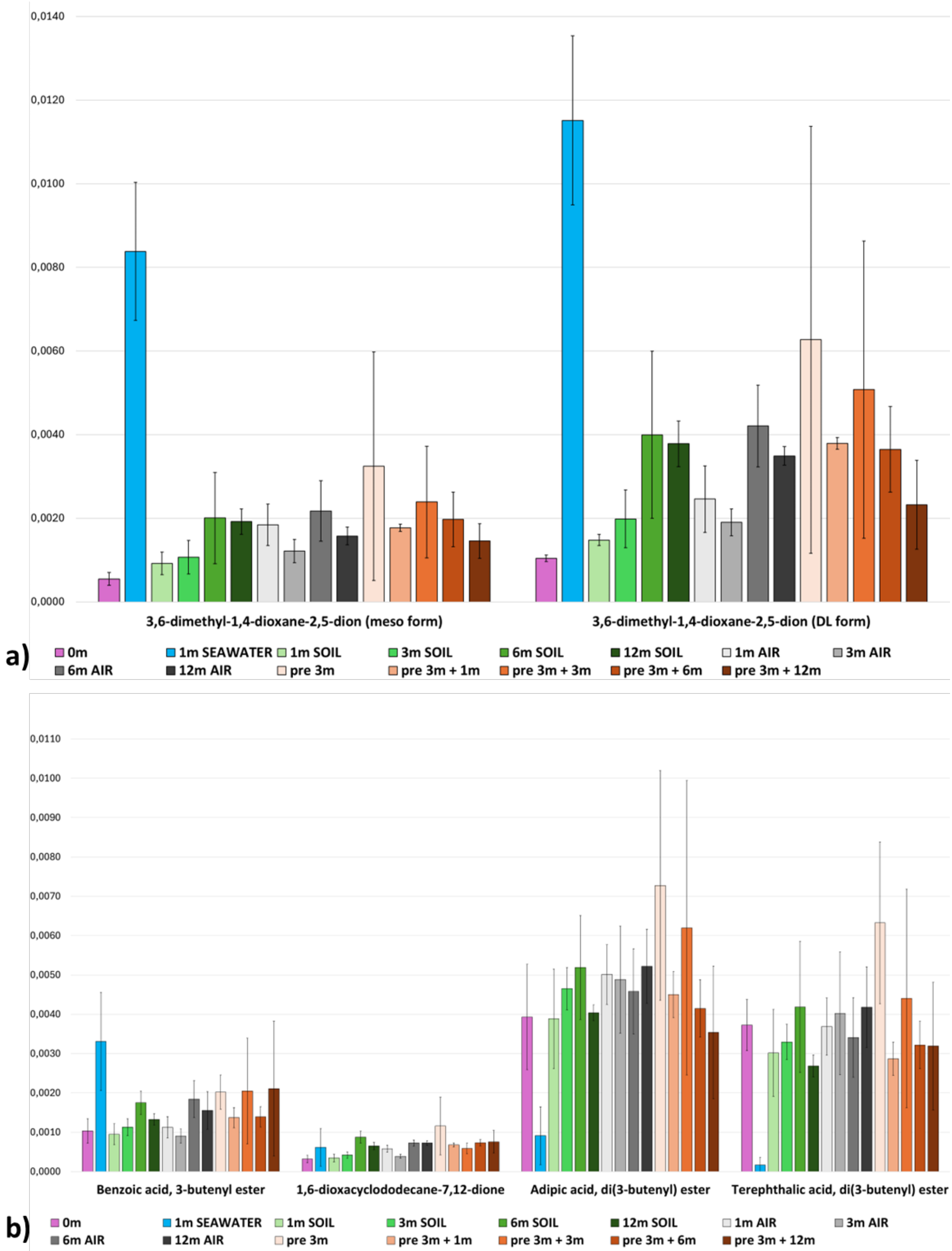

Figure S31. Peak areas integrated for replicate analyses (n=3) of BMF1 and normalized by sample weight and the peak area of the internal standard, corresponding to **a)** PLA markers and **b)** PBAT markers. “Month/s” is abbreviated as “m”.

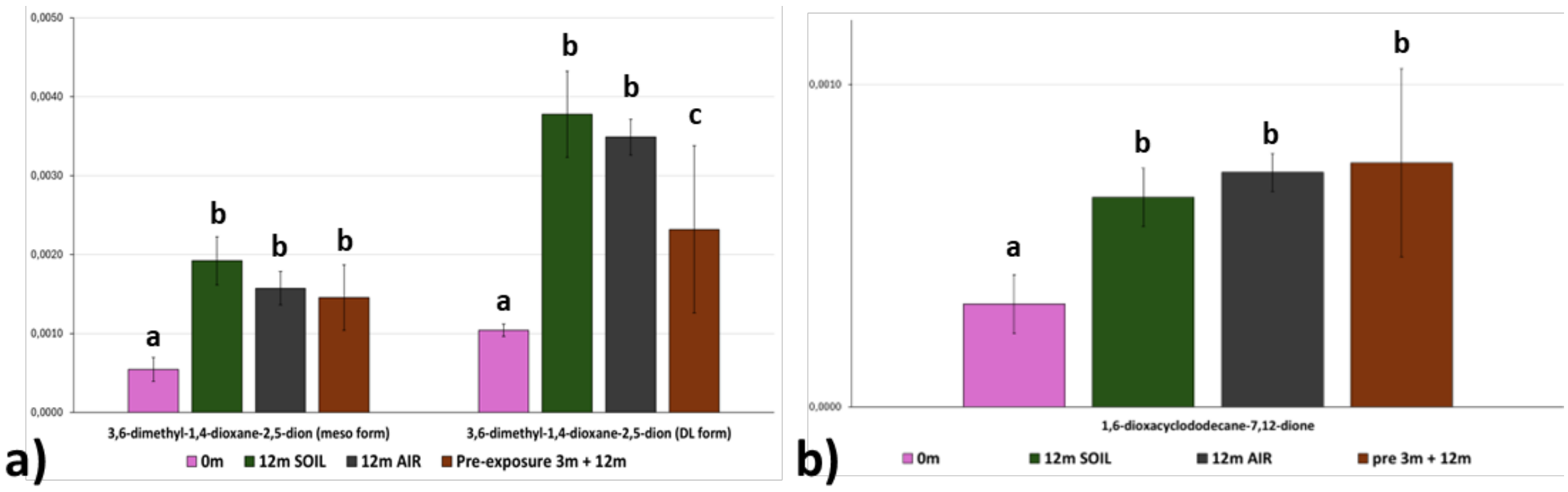

Figure S32. Peak areas integrated for replicate analyses (n=3) of BMF1 and normalised by sample weight and the peak area of the internal standards, corresponding to **a)** PLA markers of soil, air and pre-exposure samples at 12 months of exposure; **b)** PBAT marker 1,6-dioxacyclododecane-7,12-dione of soil, air and pre-exposure samples at 12 months of exposure; (n=3). Different letters indicate significant difference between the reference samples and the exposed samples detected by the SNK post hoc test. “Month/s” is abbreviated as “m”.

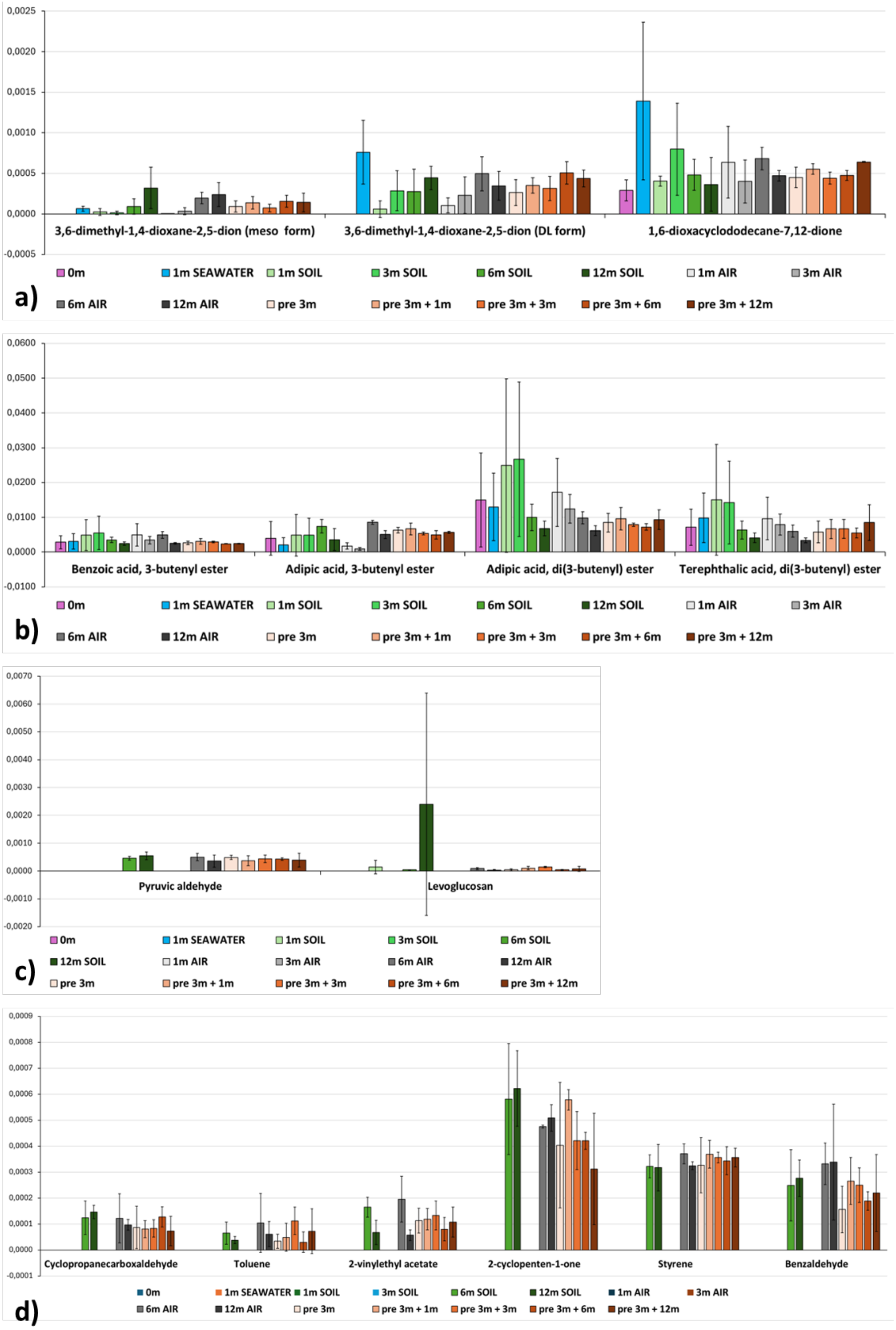

Figure S33. Peak areas integrated for replicate analyses (n=3) of BMF2 and normalised by sample weight and the peak area of the internal standard, corresponding to **a)** PLA markers and 1,6-dioxacyclododecane-7,12-dione (PBAT marker), **b)** other PBAT markers, **c)** starch markers, **d)** degradation products.

Table S11. ANOVA with SNK post hoc test on normalised peak areas of the lactide *meso* form in samples unexposed and exposed for 12 months to soil, air and pre-exposed.

ANOVA

Lmesoform

	Sum of Squares	df	Mean Square	F	Sig.
Between Groups	.000	3	.000	12.439	.002
Within Groups	.000	8	.000		
Total	.000	11			

	exposure	N	Subset for alpha = 0.05	
			1	2
Student-Newman-Keuls[a]	0m	3	.000545963	
	pre 3m+12m	3		.001457246
	12m air	3		.001573745
	12m soil	3		.001921445
	Sig.		1.000	.181

Table S12. ANOVA with SNK post hoc test on normalised peak areas of the lactide *DL* form in samples unexposed and exposed for 12 months to soil, air and pre-exposed.

ANOVA

DLform

	Sum of Squares	df	Mean Square	F	Sig.
Between Groups	.000	3	.000	12.673	.002
Within Groups	.000	8	.000		
Total	.000	11			

	exposure	N	Subset for alpha = 0.05		
			1	2	3
Student-Newman-Keuls[a]	0m	3	.001040912		
	pre 3m+12m	3		.002319225	
	12m air	3			.003490567
	12m soil	3			.003779137
	Sig.		1.000	1.000	.577

Table S13. ANOVA with SNK post hoc test on normalised peak areas of 1,6-dioxacyclododecane-7,12-dione in samples unexposed and exposed for 12 months to soil, air and pre-exposed.

ANOVA

Dion

	Sum of Squares	df	Mean Square	F	Sig.
Between Groups	.000	3	.000	4.647	.037
Within Groups	.000	8	.000		
Total	.000	11			

	exposure	N	Subset for alpha = 0.05	
			1	2
Student-Newman-Keuls[a]	0m	3	.000319341	
	12m soil	3		.000649951
	12m air	3		.000727471
	pre 3m+12m	3		.000757618
	Sig.		1.000	.705
